# Fluorinated HIV-1 protease inhibitors containing chiral hydroxyethylbenzene and indanol as P2′ ligands with potent activity against drug-resistant variants

**DOI:** 10.1016/j.ejmech.2025.118510

**Published:** 2025-12-19

**Authors:** Jagroop Kaur, Ean Spielvogel, Desaboini Nageswara Rao, Linah N. Rusere, Ala M. Shaqra, Gordon J. Lockbaum, Arooma Maryam, Nese Kurt Yilmaz, Ronald Swanstrom, Celia A. Schiffer, Akbar Ali

**Affiliations:** a Department of Biochemistry and Molecular Biotechnology, University of Massachusetts Chan Medical School, Worcester, MA, 01605, United States; b Lineberger Comprehensive Cancer Center, University of North Carolina at Chapel Hill, Chapel Hill, NC, 27599, United States; c Department of Biochemistry and Biophysics, University of North Carolina at Chapel Hill, Chapel Hill, NC, 27599, United States

**Keywords:** HIV-1 protease inhibitors, Fluorination, Drug resistance, Antiviral activity, Structure-activity relationship, X-ray structure

## Abstract

HIV-1 protease inhibitors are potent antiretroviral drugs, but their efficacy is often undermined by poor pharmacokinetics and drug resistance. Here, we employed a structure-guided design strategy to improve the potency and resistance profile of HIV-1 protease inhibitors by optimizing hydrogen bonding and van der Waals interactions within the protease substrate envelope. A series of darunavir analogs were designed by incorporating chiral 4-(1-hydroxyethyl)benzene and 1-indanol moieties as P2′ ligands, in combination with P1 fluorination. The resulting compounds showed distinct potency profiles depending on the conformational flexibility of the P2′ hydroxyl group. Notably, the P1 fluorinated compounds exhibited excellent antiviral potency against highly drug-resistant HIV-1 variants. Analysis of the protease-inhibitor cocrystal structures revealed that, similar to the 4-(1-hydroxyethyl)benzene moiety, both stereoisomers of the 1-indanol moiety make direct hydrogen bonding interactions with the backbone NH of Asp30′. To maintain polar interactions in the S2′ subsite of HIV-1 protease, the orientation of the (*R*)-indanol moiety was flipped relative to the (*S*)-1-indanol moiety. The SAR data and structural analysis offer insights for further optimization to improve potency against drug-resistant HIV-1 variants.

## 1. Introduction

Acquired Immunodeficiency Syndrome (AIDS), caused by the human immunodeficiency virus 1 (HIV-1) infection, remains one of the most significant global health issues. According to the latest Joint United Nations Program on HIV/AIDS (UNAIDS) report on Global HIV/AIDS, an estimated 40.8 million people are currently living with HIV-1 infection and 44.1 million people have died from AIDS-related causes since the start of the epidemic [1]. These numbers continue to rise at an alarming rate [2]. Despite substantial advances, individuals with HIV/AIDS still require lifelong treatment with antiretroviral therapy (ART) to suppress the viral load [3–5]. Currently, there is no cure or treatment to completely eradicate the virus from an infected person. However, the advancements in antiviral drugs targeting various steps in the HIV-1 life cycle, such as inhibitors of HIV-1 reverse transcriptase, integrase, and protease, have transformed the once fatal disease into a manageable chronic condition, significantly reducing associated morbidity and mortality [3].

HIV-1 protease inhibitors (PIs) are an important component of current ART [4,5]. To date, ten HIV-1 PIs have been approved for clinical use, with atazanavir and darunavir being the most recent [6–8]. HIV-1 PIs have several limitations, including suboptimal pharmacokinetic (PK) properties that require frequent dosing, and significant side effects such as lipid abnormalities and gastrointestinal disorders [4,9]. While most earlier PIs are no longer in clinical use, atazanavir and darunavir are key components of many current ART regimens, typically co-administered with a pharmacokinetic (PK) enhancer to allow once daily dosing [4,5]. Another major challenge is the emergence of drug resistance, which reduces the effectiveness of selected treatment over time [3–5,10]. Furthermore, central nervous system (CNS) complications remain a concern, with nearly half of patients experiencing HIV-1-associated neurocognitive disorders, partly due to inadequate CNS penetration of antiviral drugs [11–13]. These challenges highlight the need for novel PIs with improved PK and safety profiles, enhanced CNS bioavailability, and a higher barrier to resistance.

Current strategies to combat drug resistance against HIV-1 PIs primarily focus on maximizing hydrogen bonding interactions with the backbone atoms of the enzyme as well as enhancing hydrophobic interactions, particularly with residues in the S1 and S1′ subsites [14,15]. The substrate envelope model provides a rational framework for proactively avoiding drug resistance by optimizing inhibitor interactions within the substrate binding region of HIV-1 protease [15,16]. These approaches have successfully led to the development of multiple darunavir analogs with exceptional potency against drug-resistant HIV-1 variants, including DRV-resistant strains [17–20]. Analysis of structure-activity relationship (SAR) data for these DRV analogs indicates that improving potency against highly resistant HIV-1 variants requires optimization of inhibitor physicochemical properties through concurrent modifications of chemical moieties.

**Fig. 1.**
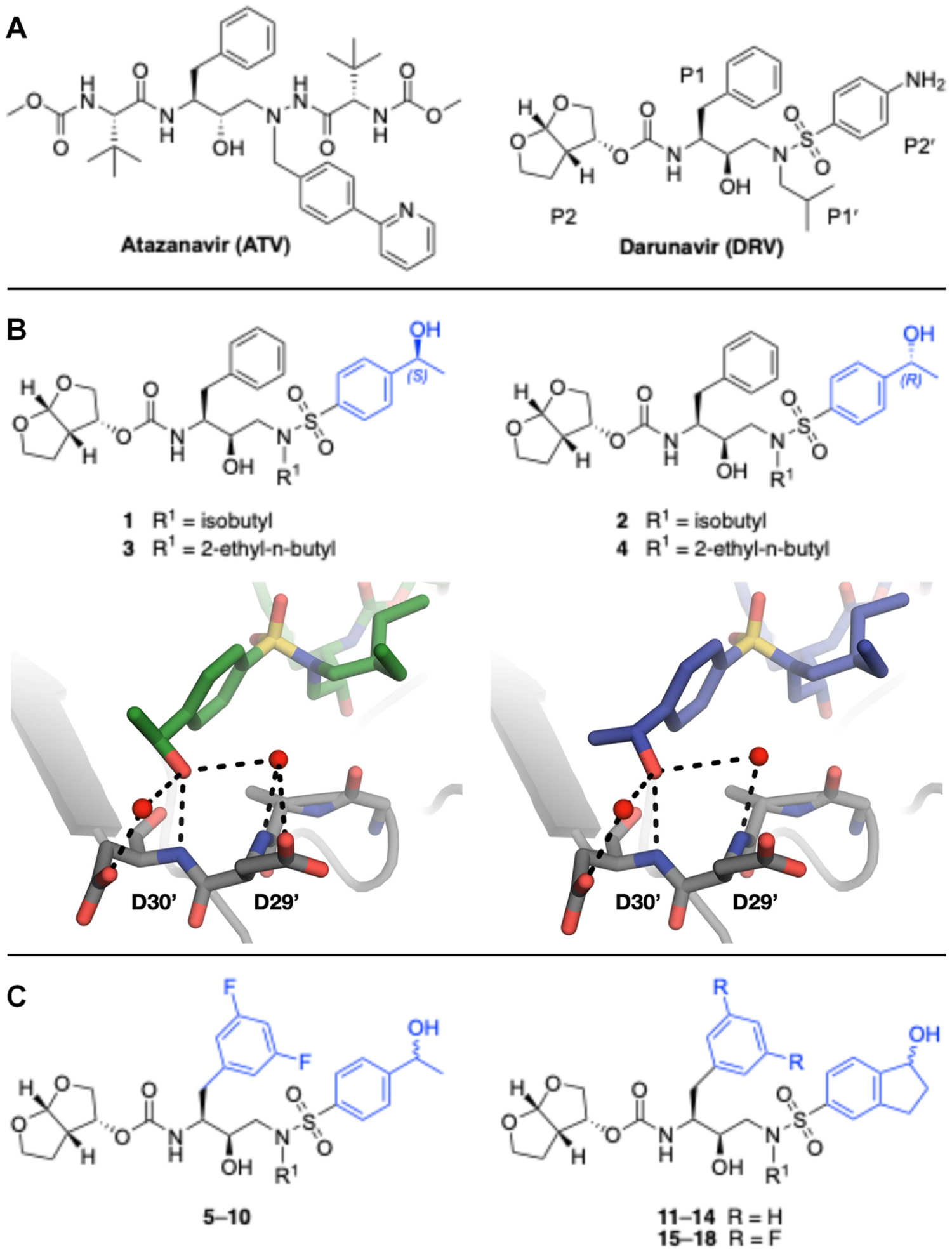
(A) Structures of FDA approved HIV-1 protease inhibitors atazanavir and darunavir. (B) Compounds **1**–**4** and the binding interactions of their P2′ moieties. X-ray cocrystal structures of compound **3** (green, PDB 6OXV) and **4** (blue, PDB 6OXW) bound to wild-type HIV-1 protease illustrating the binding interactions of P2′ moieties in the S2′ subsite of HIV-1 protease. (B) Structures of target compounds **5**–**18** with modifications at various positions evaluated in this study.

In a previous study, we employed a structure-guided design strategy to optimize hydrogen bonding and hydrophobic interactions within the substrate envelope and identified several DRV analogs with improved potency and resistance profiles [21]. Among these PIs, compounds **1**–**4** (Fig. 1A), featuring a stereochemically defined P2′ hydroxyl group designed to make enhanced polar interactions in the S2′ subsite, exhibited excellent antiviral potency against highly drug-resistant HIV-1 strains. The cocrystal structures of compounds **1**–**4** bound to wild-type HIV-1 protease revealed that the P2′ hydroxyl group is involved in a unique pattern of polar interactions in the S2′ subsite, including a direct hydrogen bond with the backbone NH of Asp30′ and water-mediated interactions with the side-chain carboxylate groups of Asp29′ and Asp30′ (Fig. 1A). Notably, in both the (*S*)- and (*R*)-stereoisomers of the P2′ 4-(1-hydroxyethyl)benzene moieties the secondary hydroxyl group was orientated toward the backbone of Asp29′ and Asp30′ despite the difference in configuration. The similar hydrogen bonding interactions observed for the P2′ epimeric compounds indicate that the conformational flexibility of the P2′ moiety, particularly the hydroxyethyl group, allowed the hydroxyl group to adopt a similar orientation in the S2′ subsite of HIV-1 protease, forcing the methyl group to adopt different orientations. The improved potency profiles of compounds **1**–**4** are likely due to their enhanced polar and hydrophobic interactions within the S2′ subsite of HIV-1 protease. Further optimization of these compounds through combinations of modifications that enhance hydrophobic interactions in the S1 and S1′ subsites can improve physicochemical properties and potentially provide compounds with a higher barrier to resistance.

In this study, a structure-guided drug design approach was employed to further optimize potency against highly drug-resistant HIV-1 variants. Based on the structural insights and established structure-activity relationship (SAR) data, analogs of compounds **1**–**4** were designed and synthesized with modifications at the P1 and P2′ positions (Fig. 1B). Specifically, compounds incorporating a (3,5-difluorophenyl)methyl moiety at the P1 position were explored to achieve enhanced hydrophobic interactions in the S1 binding pocket and potentially improve CNS penetration. In addition, compounds featuring a 1-indanol moiety at the P2′ position were investigated to assess the effect of restricting conformational flexibility of the P2′ hydroxyl group. The enzyme inhibition data revealed that the (3,5-difluorophenyl)methyl moiety at the P1 position significantly enhanced potency against a panel of drug-resistant HIV-1 protease variants. These P1 fluorinated compounds also showed improved antiviral potency against highly drug-resistant HIV-1 variants. The high-resolution cocrystal structures of compounds bound to wild-type HIV-1 protease revealed that the P2′ 4-(1-hydroxyethyl)benzene and 1-indanol moieties make similar polar interactions in the S2′ subsite, and the P1 (3,5-difluorophenyl)methyl moiety forms a network of fluorine-mediated interactions that bridge the active side residues in the S1 subsite and the flap region. The SAR data further support previous observations [14,19,22,23] indicating that an optimal combination of moieties at various inhibitor positions is critical for maintaining potency against highly drug-resistant HIV-1 variants.

## 2. Results and discussion

### 2.1. Inhibitor design

Our design strategy focused on enhancing polar interactions with the protease backbone and optimally filling the hydrophobic S1 and S1′ subsites to establish more favorable van der Waals interactions with the active site residues. Previous SAR studies of DRV analogs indicate that modifications at the P1 position can substantially improve potency against drug-resistant HIV-1 variants, especially when combined with suitable modifications at other inhibitor positions [17,19,22,24]. Among these, ether-linked phosphonate and thiazole modifications provided clinical candidates GS-8874 and brecanavir, respectively [17, 18]. Recent SAR studies of P1 phosphonate modified HIV-1 PIs indicated that the polar phosphonate moiety is beneficial for maintaining potency against drug-resistant variants, but only when combined with relatively hydrophobic moieties at the P1′ and P2′ positions [22]. A more lipophilic (3,5-difluorophenyl)methyl moiety was also investigated at the P1 position, and, when combined with modifications at the P2 and P2′ positions, resulted in exceptionally potent HIV-1 PIs [19,25]. Together, the SAR data highlight the need to balance inhibitor physicochemical properties by simultaneous modifications of chemical groups to improve potency against drug-resistant variants.

With the goal to further optimize potency, we designed analogs of compounds **1**–**4** by incorporating a P1′ (3,5-difluorophenyl)methyl moiety to increase compound lipophilicity, as well as potentially enhance CNS bioavailability (Fig. 1B). A more lipophilic P1 moiety was expected to not only enhance van der Waals interactions in the S1 subsite but also improve the overall physicochemical properties, especially when combined with a polar hydroxyl group of the P2′ moiety, which is critical for making extensive polar interactions in the S2′ subsite of HIV-1 protease. The preference for fluorine modification was due to its well-established role in enhancing lipophilicity and CNS penetration of drugs [26]. Recent studies demonstrate that P1 fluorinated darunavir analogs maintain excellent potency against highly drug-resistant HIV-1 protease variants [19,27]. We explored compounds **5**–**10** containing both stereoisomers of the P2′ 4-(1-hydroxyethyl)benzene moiety, considering the conformational flexibility of the P2′ hydroxyethyl group, in order to fully assess the impact of P1 fluorination on potency against drug-resistant variants.

In addition, we explored compounds **11**–**18**, featuring a chiral 1-indanol moiety at the P2′ position with and without a P1 (3,5-difluorophenyl)methyl modification. The stereoisomers of the 1-indanol moiety were expected to make distinct interactions with the active site residues due to restricted rotation of the hydroxyl group when compared to a more flexible hydroxyl group in the parent compounds. Moreover, we hypothesized that conformationally restricted hydroxyl group in one of the 1-indanol stereoisomers might be appropriately positioned to make direct hydrogen bonding interactions with the Asp29′ and Asp30′ residues in S2′ subsite, thereby further enhancing polar interactions in the active site of HIV-1 protease.

### 2.2. Chemistry

**Scheme 1.**
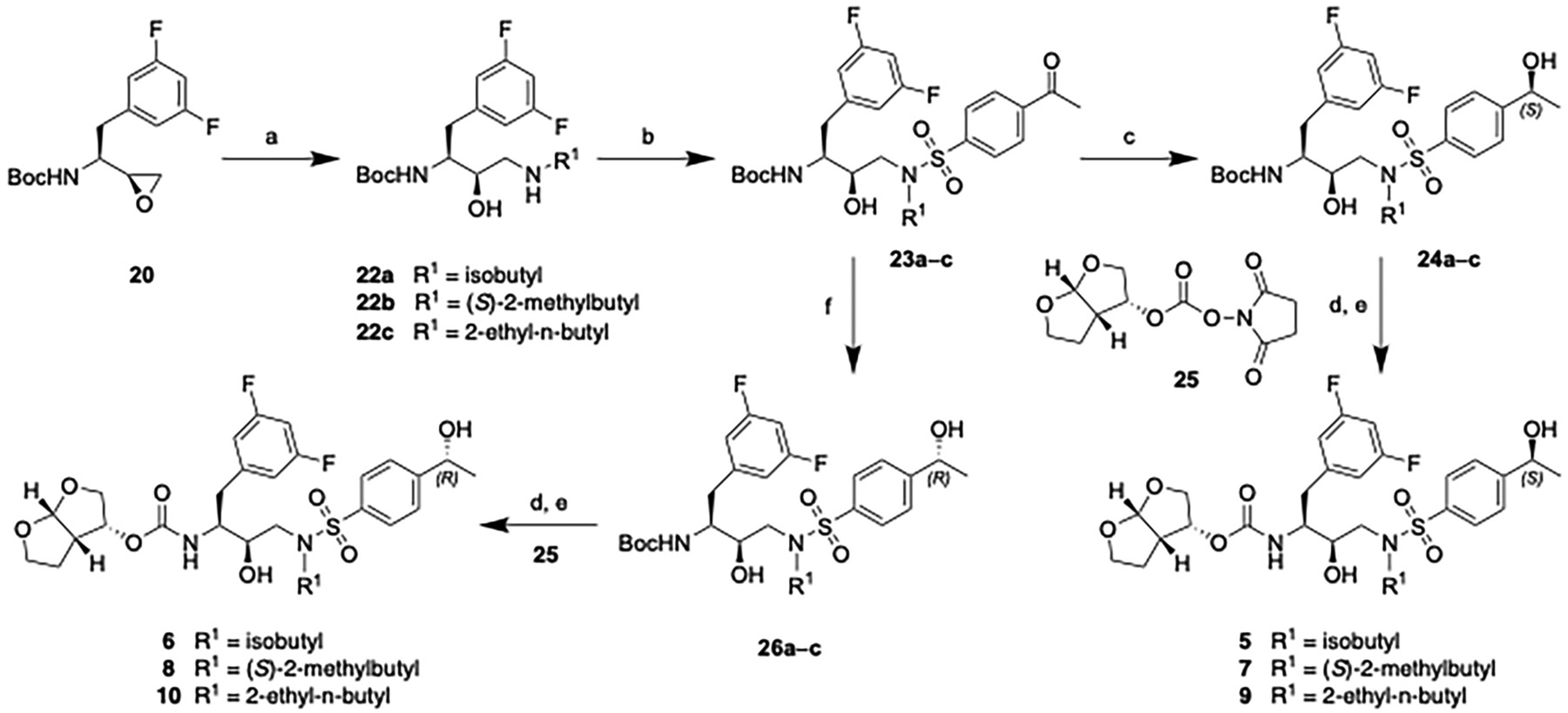
Synthesis of target compounds **5**–**10**. Reagents and conditions: (a) R^1^NH_2_, EtOH, 80 °C, 3 h, 80–96 %; (b) Na_2_CO_3_, EtOAc/H_2_O (3:1), rt, 12 h, 83–97 %; (c) (*R*)-CBS-Me, BH_3_-THF (1 M), THF, 0 °C to RT, 3 h, 85–96 %; (d) TFA, CH_2_Cl_2_, rt, 2 h, 100 %; (e) DIPEA, CH_3_CN, 0 °C to rt, 36 h, 77–82 %; (f) (*S*)-CBS-Me, BH_3_-THF (1 M), THF, 0 °C to RT, 3 h, 78–98 %.

The synthesis of target compounds **5**–**10** containing the (3,5-difluorophenyl)methyl moiety at the P1 position and a chiral 4-(1-hydroxyethyl)benzene moiety at the P2′ position is outlined in Scheme 1. Reaction of the chiral epoxide **21** with selected amines provided the amino alcohols **22a**–**c**, which were reacted with 4-acetylbenzenesulfonyl chloride using sodium carbonate as a base under biphasic conditions to provide the 4-acetylbenzenesulfonamide intermediates **23a**–**c** in excellent yield. The required chiral intermediates with stereoisomers of the 4-(1-hydroxyethyl)benzene moiety were prepared from the corresponding 4-acetylbenzene derivatives by asymmetric reduction using the Corey-Bakshi-Shibata (CBS) catalyst [28]. Accordingly, the 4-acetylbenzene intermediates **23a–c** were reacted with BH_3_-THF in the presence of the chiral catalyst (*R*)-CBS-Me to give the required intermediates (**24a**–**c**) containing the (*S*)-4-(1-hydroxyethyl)benzene moiety with excellent enantioselectivity. The purified products were recrystallized from a mixture of EtOAc/hexanes to ensure chiral purity. Boc deprotection with trifluoroacetic acid followed by reaction of the resulting amine salts with the *bis*-tetrahydrofuran (*bis*-THF) activated carbonate **25** provided the target compounds **5**, **7** and **9**. Analogs containing the (*R*)-4-(1-hydroxyethyl)-benzene at the P2′ position were synthesized via the same reaction sequence, employing the (*S*)-CBS-Me as the chiral catalyst during the stereoselective reduction step, providing the target compounds **6**, **8**, and **10**.

**Scheme 2.**
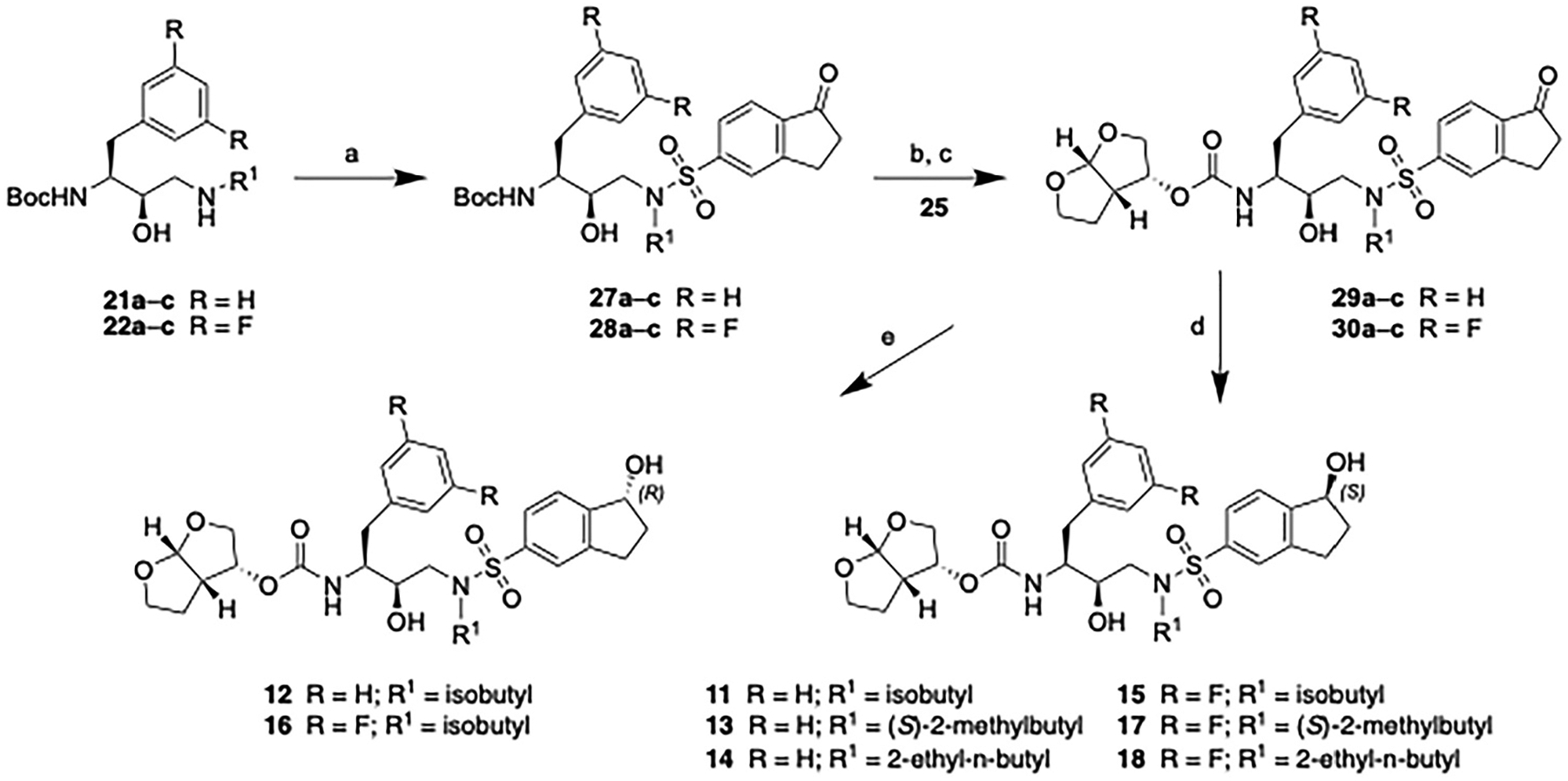
Synthesis of target compounds **11**–**18**. Reagents and conditions: (a) Na_2_CO_3_, EtOAc/H_2_O (3:1), rt, 12 h, 83–97 %; (b) TFA, CH_2_Cl_2_, rt, 2 h, 100 %; (c) DIPEA, CH_3_CN, 0 °C to rt, 36 h, 77–82 %; (d) RuCl[(*S*,*S*)-Ts-DPEN](mesitylene) (5 mol%), HCO_2_H/Et_3_N (1:2), CH_2_Cl_2_, rt, 72 h, 70–81 %; (e) RuCl[(*R*,*R*)-TsDPEN] (mesitylene) (5 mol%), HCO_2_H/Et_3_N (1:2), CH_2_Cl_2_, RT, 72 h, 77–80 %.

The synthesis of target compounds **11**–**18** with a chiral 1-indanol moiety at the P2′ is outlined in Scheme 2. The amino alcohols **21a**–**c** and **22a**–**c** were reacted with 5-indanonesulfonyl chloride in the presence of sodium carbonate under biphasic conditions to give the corresponding sulfonamide intermediates **27a**–**c** and **28a**–**c**, respectively. Based on the literature reports, the reduction of these 1-indanone derivatives using the CBS catalyst was expected to result in poor enantioselectivity [29]. Therefore, Noyori’s asymmetric transfer hydrogenation reaction [30] was considered as an alternative method, preferably as the final step in the synthesis of target compound. The required precursors of the final compounds **29a**–**c** and **30a**–**c** were prepared from the corresponding sulfonamide intermediates **27a**–**c** and **28a**–**c** in two steps, involving removal of the Boc group and reaction of the resulting amine salts with the *bis*-THF activated carbonate **25**. Gratifyingly, asymmetric transfer hydrogenation of 1-indanone derivatives using the Noyori catalyst provided the corresponding 1-indanol compounds with excellent enantioselectivity. Accordingly, treatment of intermediates **29a**–**c** and **30a**–**c** with a catalytic amount of the (*S*, *S*)-Noyori catalyst in a formic acid/triethylamine (1:2) mixture provided the target compounds **11**, **13**, and **14**, and the corresponding P1 fluorinated analogs **15**, **17**, and **18**, all containing the (*S*)-1-indanol moiety at the P2′ position. Similarly, asymmetric transfer hydrogenation reaction employing the (*R*,*R*)-Noyori catalyst afforded the target compounds **12** and **16**, containing the (*R*)-1-indanol moiety at the P2′ position.

### 2.3. Enzyme inhibition assays

The inhibitory potencies of the new compounds were evaluated against a panel of drug-resistant HIV-1 protease variants. The enzyme inhibition constants (*K*i) were determined using a highly sensitive fluorogenic assay, with DRV included as a control. Compounds were tested against two common drug-resistant protease variants, I84V and I50V/A71V, and two highly drug-resistant protease variants, 10Mut-84V and 10Mut-50V. The latter two variants, each containing 10 mutations, represent the two distinct pathways to high-level resistance, anchored by protease mutations I84V and I50V [31]. Thus these highly mutated and highly resistant protease variants are particularly suitable for evaluating the potency and resistance profile of new HIV-1 protease inhibitors.

DRV maintained pM potency against the I84V (*K*i = 0.025 nM) and I50V/A71V (*K*i = 0.075 nM) protease variants but exhibited significantly reduced potency against the two highly drug-resistant protease variants 10Mut-84V (*K*i = 150 nM) and 10Mut-50V (*K*i = 50 nM). The parent compounds **1**–**4** maintained excellent potency against the I84V and I50V/A71V protease variants, as previously reported [21]. However, similar to DRV, these compounds also exhibited significantly reduced potency against the two highly resistant variants 10Mut-84V and 10Mut-50V, with slight differences in potencies depending on the P1′ and P2′ moieties. Compared to DRV, compounds with the P1′ isobutyl and isohexyl groups retained better potency against the 10Mut-84V variant but were less active against the 10Mut-50V variant. As observed previously for the I84V and I50V/A71V variants, the stereochemistry at the P2′ 4-(1-hydroxyethyl)benzene moiety had minimal impact on potency against both 10Mut variants, with *K*i values within 2-fold for the (*S*)-and (*R*)-epimer compounds. The similar potency observed across these epimeric analogs (**1**/**2** and **3**/**4**) is attributed primarily to the flexibility of hydroxyethyl substituent at the P2′ moiety, which allows these compounds to make similar polar interactions in the S2′ subsite of the protease.

To further enhance potency against highly drug-resistant protease variants, we explored a series of protease inhibitors incorporating a (3,5-difluorophenyl)methyl moiety as the P1 ligand in combination with chiral 4-(1-hydroxyethyl)benzene moieties as P2′ ligands and three P1′ groups (Table 1). The P1 fluorinated analogs **5**–**10** displayed exceptional (pM) potency against the I84V and I50V/A71V protease variants, with *K*i values either in the low pM range or below the assay detection limit (5 pM). Most importantly, compared to the parent compounds, the fluorinated analogs exhibited markedly improved potency against the two highly resistant 10Mut protease variants. Compound **5**, with an isobutyl group at the P1′ position and (*S*)-4-(1-hydroxyethy)benzene moiety at the P2′ position, displayed a 4-fold and 17-fold improvement in potency against the 10Mut-84V (*K*i = 24 nM) and 10Mut-50V (*K*i = 15 nM) protease variants, respectively, as compared to the parent compound **1**. The corresponding P2′ epimer compound **6** with the (*R*)-4-(1-hydroxyethy)benzene moiety was even more active against the 10Mut-84V protease variant, maintaining low nM potency against this variant. Similar trends were observed for compounds with the P1′ (*S*)-2-methy-butyl group, with the P2′ (*R*)-stereoisomer **8** (*K*i = 6 nM) showing a 2-fold better potency against the 10Mut-84V protease variant than the corresponding P2′ (*S*)-stereoisomer **7** (*K*i = 14 nM). Increasing the hydrophobic bulk at the P1′ position by incorporating a larger isohexyl group further improved potency of the resulting compounds **9** (*K*i = 4.3 nM) and **10** (*K*i = 1.3 nM) against the 10Mut-84V variant. However, their potency against the 10Mut-50V variant remained comparable to that observed for compounds with the P1′ isobutyl and isopentyl groups (**5**–**8**). For all three pairs of P2′ epimeric compounds, the configuration of the 4-(1-hydroxyethyl)benzene moiety had minimal impact on the inhibitory activity against both 10Mut protease variants, likely due to the conformational flexibility of the 1-hydroxyethyl substituent in the S2′ subsite of HIV-1 protease.

As fluorine has unique physicochemical and electronic properties and a larger van der Waals radius, the P1 (3,5-difluorophenyl)methyl moiety resulted in enhanced van der Waals interactions as well as fluorine-mediated interactions in the protease active site, as shown previously [19,25,27]. Consistent with this, compounds **5**–**10** with the P1 (3,5-difluorophenyl)methyl moiety displayed significant enhancement in the enzyme inhibitory activity against drug-resistant protease variants tested, as compared to their corresponding non-fluorinated counterparts (**1**–**4**). Notably, the fluorinated analogs displayed significantly improved potency against the highly resistant protease variants compared to DRV. Overall, a combination of a P1 (3,5-difluorophenyl) methyl moiety and P2′ 4-(1-hydroxyethyl)benzene moieties, along with a larger hydrophobic moiety at the P1′ position, resulted in inhibitors with excellent potency against highly drug-resistant protease variants.

Next, we evaluated a series of protease inhibitors (**11**–**14**) containing a chiral 1-indanol moiety at the P2′ position along with three P1′ modifications. The stereoisomers of the bicyclic 1-indanol moiety were expected to interact differently with the residues in the S2′ subsite due to restricted conformation of the hydroxyl group, while making additional hydrophobic interactions. Compared to the parent compound **1**, analog **11** with the P2′ (*S*)-1-indanol moiety was more active against the 10Mut-50V variant but was 4-fold less active against the 10Mut-84V variant (Table 2). The corresponding analog **12** with the P2′ (*R*)-indanol stereoisomer was equipotent to **2** against the I84V variant but showed reduced potency against the other three protease variants. While both compounds **11** and **12** showed similar inhibition of the 10Mut-84V variant regardless of the 1-indanol stereochemistry at the P2′ position, their potencies differed significantly against the other three protease variants. Thus, as expected, restricting the conformational flexibility of the hydroxyl group resulted in distinct potency profiles for the pair of P2′ epimeric compounds, especially against the protease variants containing the I50V mutation.

Introducing larger P1 groups in combination with the (*S*)-1-indanol moiety at the P2′ position provided compounds with potency profiles comparable to that of the corresponding isobutyl analog **11**. Compound **13** with a P1′ isopentyl group was equipotent to **11** against the 10Mut-84V protease variant but exhibited slightly lower potency against the 10Mut-50V variant. The analog **14** with the P1′ isohexyl group and P2′ (*S*)-1-indanol moiety displayed a 2-fold improvement in potency against the 10Mut-84V protease variant. In this series, only compound **14** exhibited improved potency against all the protease variants tested compared to the corresponding P1′ isobutyl and isopentyl analogs (**11**–**13**).

As observed for the P2′ 4-(1-hydroxyethyl)benzene compound series, incorporating a (3,5-difluorophenyl)methyl moiety at the P1 position in combination with a chiral 1-indanol moiety significantly improved the potency of the resulting analogs (**15**–**18**) compared to the non-fluorinated compounds (**11**–**14**), as well as the parent compounds (**1**–**4**). The P1 fluorinated compound **15**, containing the P2′ (*S*)-1-indanol moiety, maintained nM activity against the 10Mut-84V (*K*i = 54.4 nM) and 10Mut-50V (12.8 nM) protease variants. The corresponding analog **16** with the (*R*)-1-indanol configuration retained similar potency against the 10Mut-84V (*K*i = 56.4 nM) protease variant but was less active against the other three variants. Consistent with the trend seen with the non-fluorinated compounds (**11** and **12**), the P1 fluorinated compound **15** with the P2′ (*S*)-1-indanol moiety was more potent compared to the corresponding (*R*)-1-indanol analog **16**. When compared with the corresponding P2′ 4-(1-hydroxyethyl)benzene analogs (**5**–**6**), the (*R*)-epimer **16** was less active compared to **6**, whereas the (*S*)-epimer **15** displayed a potency profile comparable to that of **5**.

Compound **17** featuring an P1′ isopentyl group and the P2′ (*S*)-1-indanol moiety also exhibited improved potency against all the protease variants tested when compared to its non-fluorinated analog **13**. Incorporating a more hydrophobic isohexyl moiety at P1′ together with the (*S*)-1-indanol at P2′ resulted in further potency gains, particularly against the 10Mut-84V protease variant, in comparison to the P1′ isobutyl analog (compound **18** versus **15**). Notably, compound **18** displayed low pM potency (<5 pM) against the I84V and I50V/A71V variants. Overall, compared to the parent compounds (**1**–**4**), incorporating a chiral 1-indanol moiety at the P2′ position in combination with a (3,5-difluorophenyl)methyl moiety at the P1 position, resulted in improved potency against highly drug-resistant HIV-1 protease variants.

### 2.4. Antiviral assays

The antiviral activity of the new compounds was evaluated against wild-type HIV-1 (NL4-3 strain) and two drug-resistant viral variants in a cell culture-based viral inhibition assay (Table 3). As with the protease variants tested in enzyme inhibition assays, the two drug-resistant HIV-1 variants, HIV-1-DRV and HIV-1-V9, are representative of the two distinct pathways (anchored by protease mutations I84V and I50V, respectively) of high-level resistance. These viral variants were identified in viral passaging experiments with increasing concentrations of DRV and analogs and contain 10–14 amino acid substitutions in the protease [31]. Notably, the HIV-1-DRV viral variant contains the same 10 acid substitutions in the protease as the 10Mut-84V protease variant used in the enzymatic assays. As expected, the drug-resistant viral variants showed reduced susceptibility to DRV. Compared to wild-type HIV-1 (EC_50_ = 0.009 μM), DRV exhibited markedly lower antiviral potency against HIV-1-DRV (EC_50_ = 0.69 μM) and HIV-1-V9 (EC_50_ = 1.24 μM) viral strains, resulting in a 77- and 138-fold reduction in potency, respectively. Thus, both viral variants exhibited high-level resistance to DRV.

The P1 fluorinated analogs (**5**–**10**) of the parent compounds showed excellent antiviral potency against wild-type HIV-1, with EC_50_ values comparable to that of DRV (EC_50_ = 0.009 μM). These compounds exhibited significant enhancements in antiviral potency against the drug-resistant variant HIV-1-DRV compared to DRV, as well as a notable improvement against the HIV-1-V9 variant. Compound **5**, with the P1′ isobutyl group and P2′ (*S*)-4-(1-hydroxyethyl)benzene moiety, showed EC_50_ values of 0.02 μM and 0.49 μM against the HIV-1-DRV and HIV-1-V9 variants, respectively. This represents a 35-fold and 2-fold improvement in potency compared to DRV. In comparison, the diastereoisomer **6** with the P2′ (*R*)-4-(1-hydroxyethyl)benzene moiety was less active than **5**. Still, compound **6** exhibited improved potency against the HIV-1-DRV viral variant (EC_50_ = 0.14 μM) relative to DRV. Thus the 3,5-diflurobenzene moiety at the P1 position appeared to greatly contribute to the antiviral activity of compounds against highly drug-resistant HIV-1 variants.

The P1 fluorinated analogs with the P1′ isopentyl group (compounds **7**–**8**) also exhibited improved antiviral activity compared to DRV, especially against the HIV-1-DRV viral variant. The P2′ (*S*)-stereoisomer **7** displayed an EC_50_ value of 0.04 μM against the HIV-1-DRV variant, reflecting a 17-fold improvement in potency over DRV. This analog was also among the most potent against the HIV-1-V9 variant (EC_50_ = 0.23 μM), indicating a 5-fold improvement in potency relative to DRV. The corresponding P2′ (*R*)-stereoisomer **8** achieved low nanomolar potency (EC_50_ < 0.005 μM) against the HIV-1-DRV variant and showed improved potency against the HIV-1-V9 variant. Similar trends were observed with compounds containing a more hydrophobic isohexyl group at the P1′ position. The P2′ epimeric compounds **9** and **10** both exhibited exceptional antiviral potency against the HIV-1-DRV variant (EC_50_ < 0.005 μM), while also showing a 2-fold enhancement in potency against the HIV-1-V9 variant. Overall, while significant enhancement in antiviral potency was realized against the HIV-1 variant HIV-1-DRV, improving potency against the HIV-1-V9 proved to be more challenging. Nevertheless, the P1 fluorinated compounds maintained better antiviral potency against both drug-resistant variant relative to DRV.

The antiviral activities of P1 fluorinated compounds, especially against the HIV-1-DRV viral variant, showed a clear correlation with increasing hydrophobic bulk at the P1′ position. In this series, the stereochemistry of the P2′ 4-(1-hydroxyethyl)benzene moiety had a minimal effect on antiviral activity against drug-resistant HIV-1 variants, especially for compounds with a larger hydrophobic group at the P1′ position. These observations align with enzymatic assay data, suggesting that compounds with a larger hydrophobic group at the P1′ position that optimally fills the S1′ subsite of protease generally show improved potency against the drug-resistant variants.

Next, the antiviral activity of the P2′ 1-indanol series of HIV-1 protease inhibitors was evaluated (Table 3). The P2′ 1-indanol analogs **11**–**14** with a P1 phenylmethyl moiety showed potent antiviral activity against the wild-type HIV-1, with EC_50_ values ranging from 0.012 μM to 0.025 μM. Among these, compounds **12** and **13** were 2-fold less active than DRV against wild-type HIV-1, although they exhibited similar potencies in enzymatic assays against wild-type HIV-1 protease (Table S1). The P2′ 1-indanol analogs showed a range of potencies against the drug-resistant HIV-1 variants, depending on the P1′ hydrophobic group, but were generally less active than DRV. The P1′ isobutyl analogs **11** and **12** containing the (*S*)- and (*R*)-1-indanol moiety at the P2′ position, respectively, exhibited similar potencies against the two drug-resistant HIV-1 variants. While these analogs maintained similar potency as DRV against the HIV-1-DRV variant, they were significantly less active against the HIV-1-V9 variant, losing 300-fold potency relative to wild-type HIV-1. The P1′ isopentyl analog **13** showed reduced potency against both drug-resistant viral variants compared to DRV. The P1′ isohexyl analog **14** displayed a potency profile comparable to that of the corresponding isobutyl analog **11**. Thus, replacing the 4-(1-hydroxyethyl)benzene moiety with a bicyclic 1-indanol moiety at the P2′ position was detrimental to potency, especially against the HIV-1-V9 variant. The SAR data indicate that the conformational flexibility of the P2′ moiety, especially the hydroxyl group, is important for maintaining activity against highly drug-resistant HIV-1 variants.

The P1 fluorinated compounds **15**–**18** with a P2′ chiral 1-indanol moiety maintained excellent antiviral activity against drug-resistant HIV-1 variants. Compared to DRV, compound **15**, containing the isobutyl group at P1′ and (*S*)-1-indanol at P2′ in combination with the (3,5-difluorophenyl)methyl moiety at P1, showed a 2-fold improvement in antiviral potency against the HIV-1-DRV variant. The corresponding P2′ (*R*)-1-indanol analog **16** displayed a 5-fold enhancement in potency against the HIV-1-DRV variant (EC_50_ = 0.13 μM) and was equipotent to DRV against the HIV-1-V9 variant. For both compounds the fold potency losses were lower than those of DRV. The P1′ isopentyl analog **17** experienced a 3-fold loss in potency against the HIV-1-V9 variant relative to DRV but exhibited comparable potency against the HIV-1-DRV variant (EC_50_ = 0.57 μM). Compound **18**, with a more hydrophobic isohexyl moiety at P1′ and (*S*)-1-indanol at P2′, showed a 4-fold improvement in potency against the HIV-1-DRV (EC_50_ = 0.12 μM) variant compared to its non-fluorinated analog **14** (EC_50_ = 0.50 μM), as well as darunavir. Overall, consistent with the biochemical data, the P1 fluorinated compounds (**15**–**18**) displayed notable improvements in antiviral activity against the highly resistant HIV-1 strains compared to their non-fluorinated analogs (**11**–**14**), irrespective of the configuration of the hydroxyl group at the P2′ 1-indanol moiety.

The antiviral data against drug-resistant HIV-1 variants are consistent with the results from enzymatic assays, indicating that restricting the flexibility of the P2′ hydroxyl group by replacing the 4-(1-hydroxyethyl)benzene moiety with 1-indanol is generally not advantageous, except when combined with the P1 (3,5-difluorophenyl)methyl moiety. The SAR data also indicate that increasing the hydrophobic bulk at the P1′ position generally improved potency. Among these analogs, the P1 fluorinated compounds **5**–**10**, which incorporate stereoisomers of the P2′ 4-(1-hydroxyethyl)benzene moiety featuring a flexible secondary hydroxyl group, emerged as the most potent inhibitors against highly drug-resistant HIV-1 variants.

Overall, these results further support the observations from previous SAR studies on the DRV scaffold [17,21], which identified compound hydrophobicity (as quantified by cLogP) as a critical determinant of cellular antiviral activity against both wild-type and drug-resistant HIV-1 strains. The results further emphasize that, in addition to enhancing hydrogen bond interactions with the backbone atoms in the protease active site, optimizing hydrophobic interactions is critical to enhancing potency across diverse drug-resistant viral strains. Moreover, the SAR data provide crucial insights into the chemical features influencing inhibitor potency and resistance profile. Notably, modifications at the P1 and P1′ positions on the inhibitor scaffold emerge as key determinants for maintaining activity against drug-resistant variants, offering opportunities for further optimization.

### 2.5. Analysis of protease-inhibitor complexes

To elucidate the protease-inhibitor binding interactions, we determined high-resolution cocrystal structures of nearly all new compounds bound to wild-type HIV-1 protease (NL4-3 strain). A total of 13 new cocrystal structures were determined with a resolution ranging from 1.72 to 1.95 Å; the crystallographic data collection and refinement statistics are provided in Tables S2–S4 (Supplementary Information). The cocrystal structures were determined in the P6_1_2_1_2_1_ space group, except for four compounds which crystalized in the *P*2_1_2_1_2_1_ space group, all with one protease homodimer in the asymmetric unit and only one orientation of the bound inhibitor in the protease active site. The cocrystal structures of the parent compounds (**1**–**4**) in complex with wild-type protease were previously determined in the *P*2_1_2_1_2_1_ space group [21]. The analysis and comparison of these cocrystal structures provided insights into the changes in protease-inhibitor binding interactions arising from specific inhibitor modifications at various positions.

The cocrystal structures of the new HIV-1 PIs were superimposed with that of DRV and the parent compounds to enable a direct structural comparison. All compounds adopted the same overall binding conformation as DRV and the parent compounds (**1**–**4**), as expected. However, notable differences were observed in the S2′ subsite, where the P2′ 4-(1-hydroxyethyl)benzene and 1-indanol moieties bind, as well as in the S1 subsite, where the P1 (3,5-difluorophenyl)methyl group binds (Figure S1 and Figure S2).

**Fig. 2.**
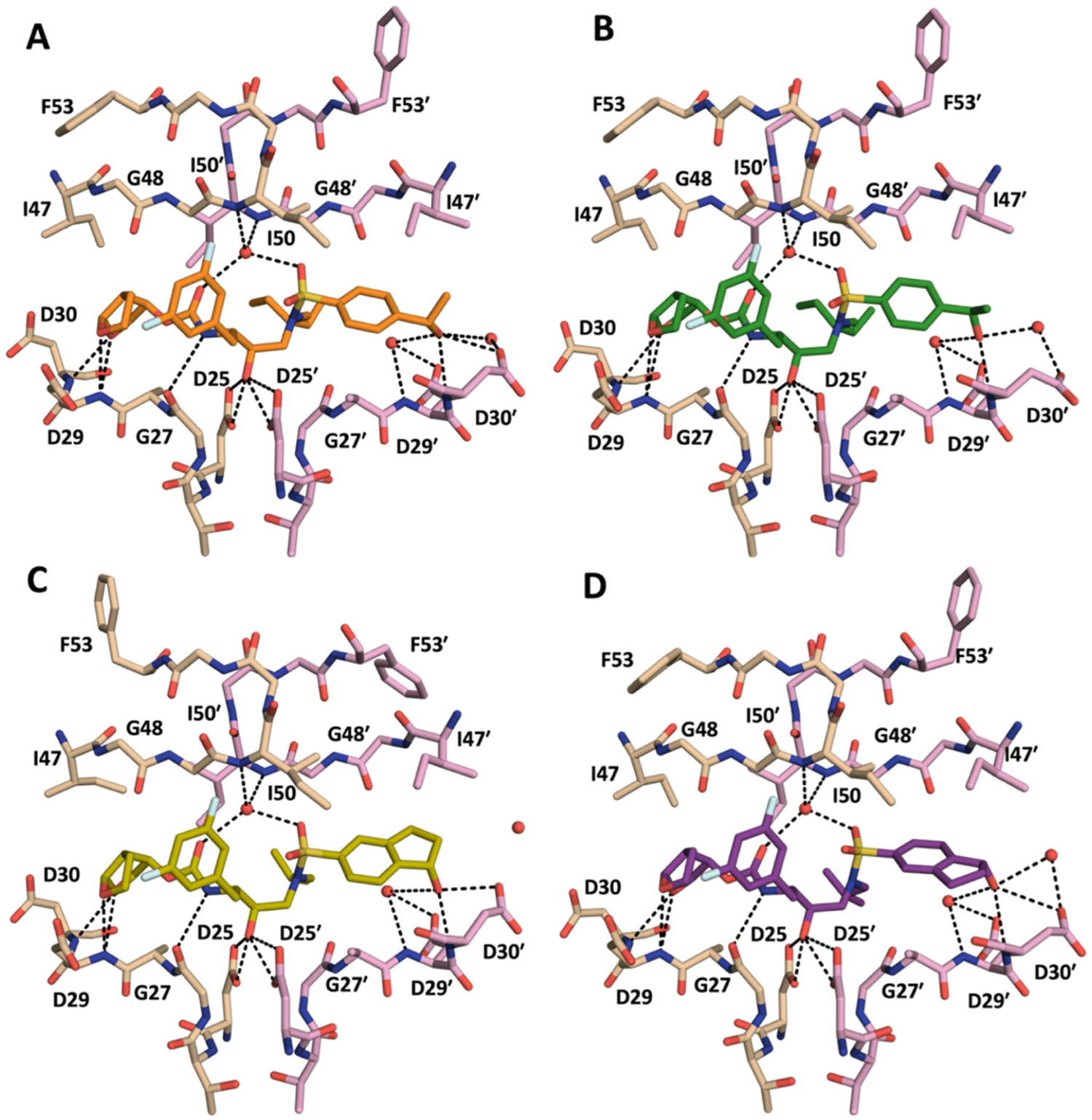
Crystal structures of wild-type HIV-1 protease in complex with inhibitors (A) **9**, (B) **10**, (C) **15**, and (D) **16**. The two protease monomers are shown in gold (denoted as nonprime) and pink (denoted as prime) colors. The key hydrogen bond interactions between the inhibitors and the active site residues of HIV-1 protease are shown as black dashed lines.

In the cocrystal structures, compounds **5**–**10** incorporating the P1 (3,5-difluorophenyl)methyl moiety and stereoisomers of the P2′ 4-(1-hydroxyethyl)benzene moiety make similar hydrogen bonding interactions with the protease as the parent compounds (**1**–**4**) (Fig. 2, Fig. S3). These include hydrogen bond interactions of the P2 *bis*-THF moiety with the backbone NH of Asp29 and Asp30 residues in the S2 binding pocket, water mediated hydrogen bond network with the flap residues, and the crucial interactions of the central hydroxyl group with the catalytic Asp25 and Asp25′ residues. In the S2′ subsite, the secondary hydroxyl group of both the (*S*)- and (*R*)-4-(1-hydroxyethyl)benzene moieties is oriented toward the Asp29′ and Asp30′ residues, forming a direct hydrogen bond with the backbone NH of Asp30′ (Fig. 2A and B) (Table S5). This hydroxyl group also interacts with the side chain carboxylates of both Asp29′ and Asp30′ through water-mediated hydrogen bonds. This extensive hydrogen-bonding network in the S2′ subsite likely contributed to the enhanced potency of these compounds compared to DRV.

**Fig. 3.**
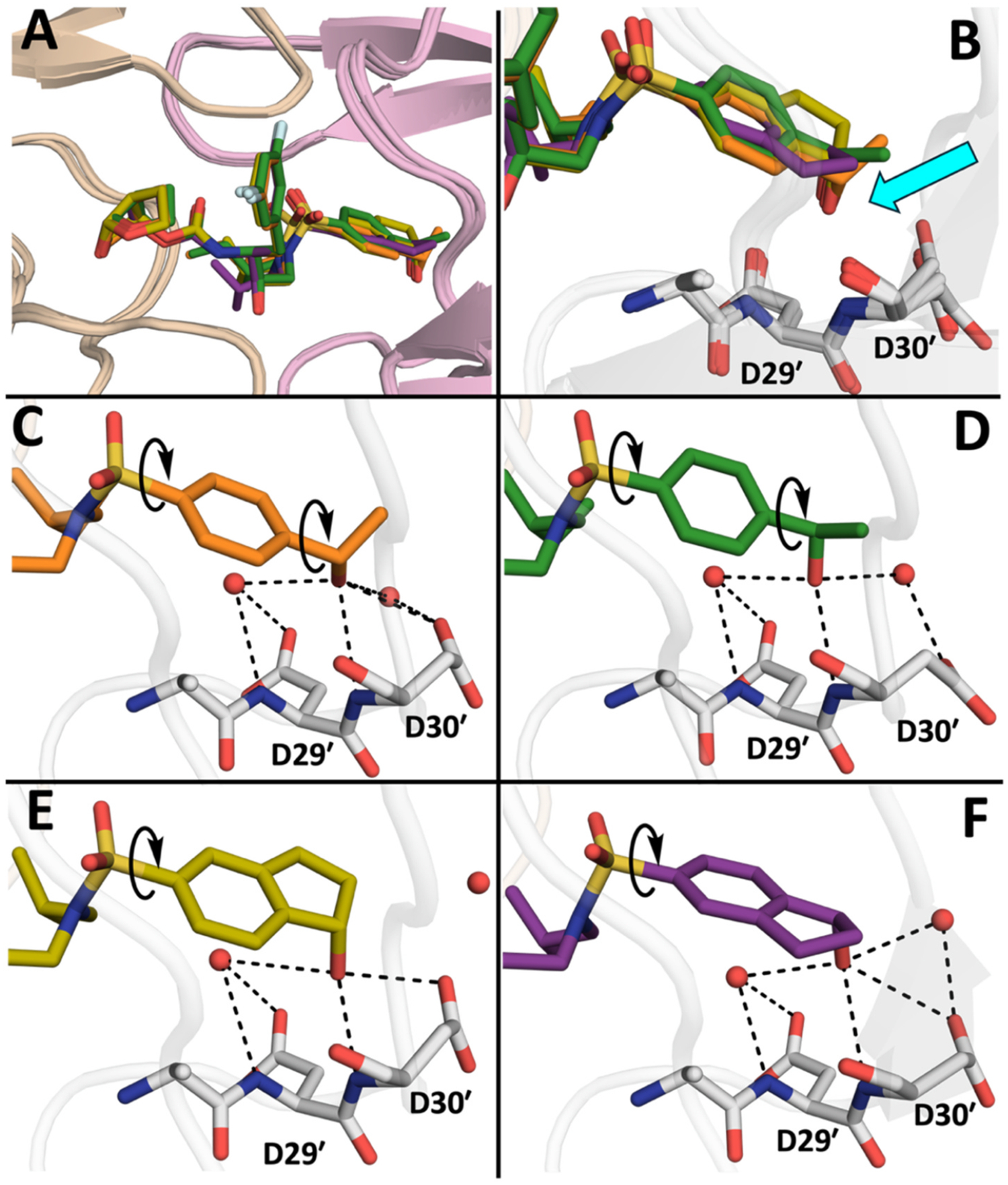
Comparison of inhibitor binding interactions in the S2′ subsite of HIV-1 protease. (A) A superimposed view of compounds **9**, **10**, **15** and **16** bound in the active site of wild-type HIV-1 protease. (B) Zoomed-in view of superimposed P2′ moieties, showing similar orientation of hydroxyl groups (indicated by a cyan arrow) toward the backbone of Asp29′ and Asp30′ residues in the S2′ subsite of HIV-1 protease, irrespective of the stereochemistry at the P2′ 1-(hydroxyethyl)benzene and 1-indanol moieties. (C) Compound **9** with the P2′ (*S*)-4-(1-hydroxyethyl)benzene. (D) Compound **10** with the P2′ (*R*)-4-(1-hydroxyethyl)benzene. (E) Compound **15** with the P2′ (*S*)-1-indanol. (F) Compound **16** with the P2′ (*R*)-1-indanol.

As observed for the parent compounds, in protease complexes with compounds **5**–**10**, both the (*S*)- and (*R*)-stereoisomers of the P2′ 4-(1-hydroxyethyl)benzene moiety form similar hydrogen bonding interactions in the S2′ binding pocket, primarily due to the flexibility of the hydroxyethyl group (Fig. 3C and D). The hydroxyl group of the (*R*)-4-(1-hydroxyethyl)benzene moiety appears to adopt a more favorable orientation toward the protease backbone compared to the (*S*)-4-(1-hydroxyethyl)benzene moiety (Fig. 3C and D), although distances to the backbone NH of Asp30 are similar.

The cocrystal structures of compounds incorporating the P2′ 1-indanol moiety (compounds **11**–**16** and **18**) revealed an overall similar binding conformation as the P2′ 4-(1-hydroxyethyl)benzene analogs, with key protease-inhibitor interactions within the S1, S2 and S2′ subsites maintained (Fig. 2C and D, Figure S4). We hypothesized that restricting the conformational flexibility of the P2′ moiety would result in distinct interactions of the hydroxyl group with the residues in the S2′ subsite. However, in cocrystal structures, both stereoisomers of the P2′ 1-indanol moiety make comparable interactions in the S2′ subsite. For example, the 1-indanol moieties of compounds **15** and **16** form similar polar interactions regardless of the 1-indanol stereochemistry (Fig. 3E and F). The hydroxyl group of the (*S*)-1-indanol moiety in **15** is oriented toward the Asp29′ and Asp30′ residues, forming direct hydrogen bonds with the Asp30′ backbone NH and sidechain carboxylate, and water-mediated hydrogen bonding interactions with the Asp29′ side chain (Fig. 3E). The hydroxyl group of the (*R*)-1-indanol in **16** also makes similar hydrogen bonding interactions with the Asp29′ and Asp30′ residues, but with the indanol moiety flipped relative to the conformation in **15** (Fig. 3F), resulting in similar polar interaction in the S2′ subsite. This likely explains the largely similar potencies of compounds **15** and **16** against the 10Mut-84V variant. Nevertheless, differences in orientation were observed in the binding of the 1-indanol moieties depending on the configuration of the hydroxyl group, with the (*S*)-1-indanol methylene carbons facing toward flaps and the (*R*)-1-indanol methylene toward the Asp30′ sidechain (Fig. 3E and F). The hydroxyl group of **15** with the (*S*)-1-indanol moiety is closer to Asp29′ and Asp30′, thus making stronger hydrogen bond interactions with the backbone NH of Asp30′ (2.9 Å) compared to compound **15** (3.4 Å) with the (*R*)-1-indanol. In addition, the (*S*)-1-indanol moiety of **15** displaced one of the water molecules in the S2′ subsite, making a direct hydrogen bond with the side chain of Asp30′.

**Fig. 4.**
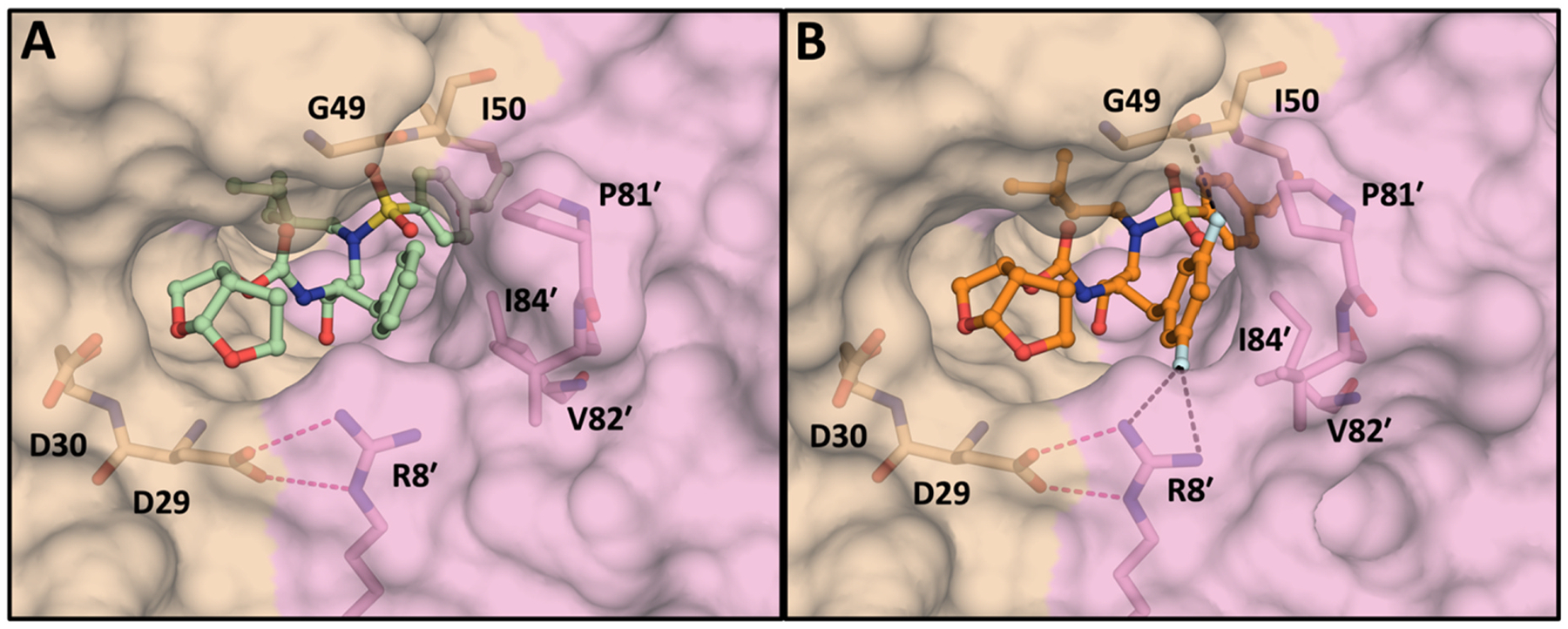
Surface view of HIV-1 protease active site, comparing the packing of P1 moieties in the S1 subsite of HIV-1 Protease. (A) Compound **3** with a P1 phenylmethyl moiety. (B) compound **9** with a P1 (3,5-difluorophenyl)methyl moiety. The two protease monomers are in gold (denoted as nonprime) and pink (denoted as prime) color. The fluorine-mediated multipolar interactions are shown in black dashed lines and electrostatic interactions between Asp29 and Arg8′ are in magenta dashed lines.

**Fig. 5.**
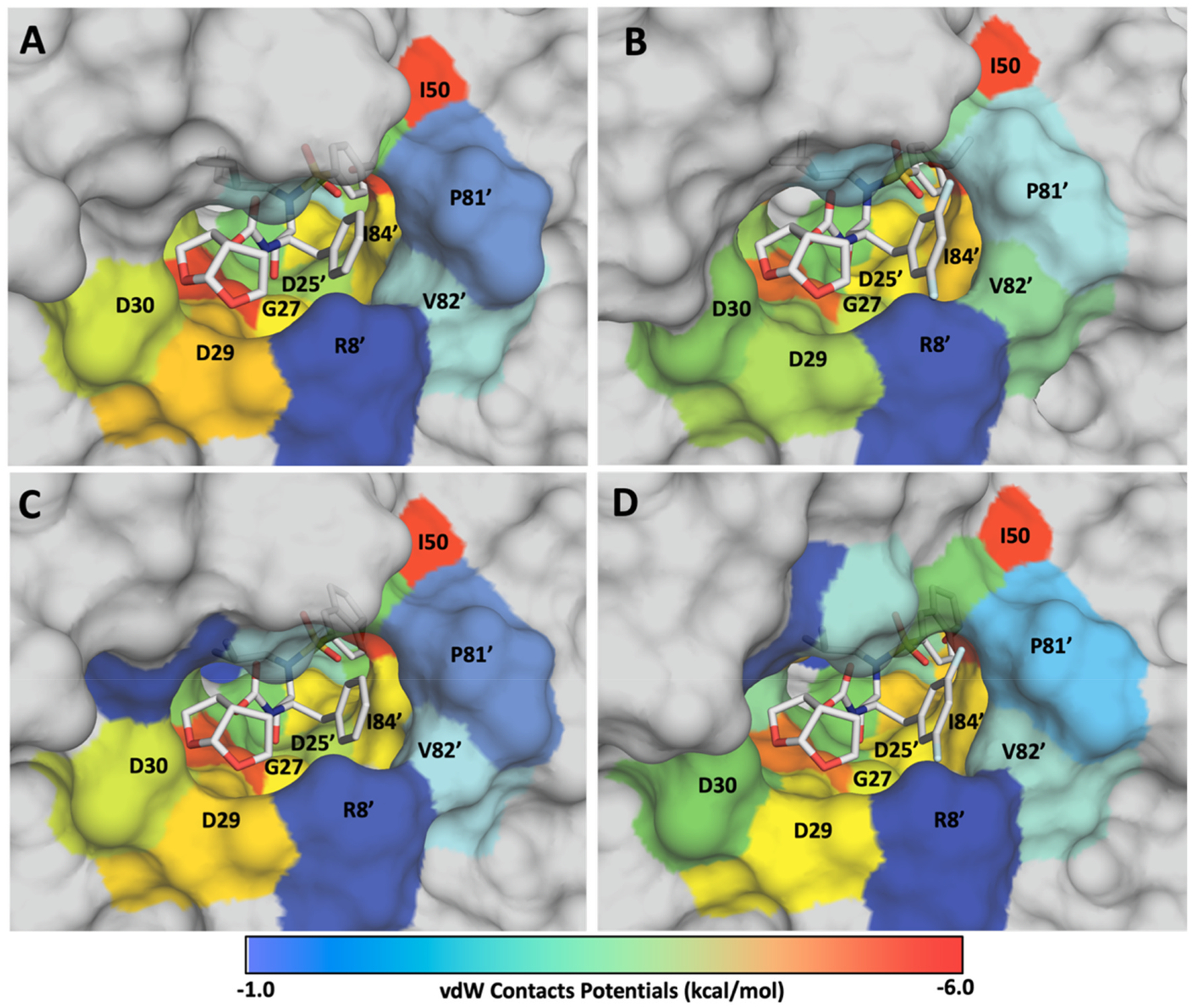
Comparison of van der Waals contacts between the P1 fluorinated and non-fluorinated HIV-1 protease inhibitors. Structures showing packing of the P1 phenyl ring of compounds **3** (A) and **11** (C) in comparison to the P1 difluorophenyl ring of compounds **9** (B) and **15** (D) within the S1 binding pocket of HIV-1 protease. The active site residues of HIV-1 protease pocket are colored from blue to red rainbow spectrum, indicating increase of van der Waals contact energies with the inhibitors mapped onto the cocrystal structures. Only the residues showing considerable differences due to the addition of fluorine atoms at the P1 moiety are labelled to highlight the structural impact of fluorine addition.

The cocrystal structures of P1 fluorinated compounds (**5**–**10**) showed minor conformational differences in the P1 (3,5-difluorophenyl)methyl moiety compared to the phenylmethyl moiety of the parent compounds (**1**–**4)** within the S1 subsite (Fig. S1D). The P1 (3,5-difluorophenyl) methyl moiety was observed to make multipolar and enhanced van der Waals interactions with both the flap residues and residues in the S1 subsite (Fig. 4). The two fluorine atoms on the P1 phenyl ring bridged the active site residues in the S1 subsite and the flap region by forming a network of multipolar interactions [19,27]. These interactions involved the conserved residue Arg8′ as well as the backbone NH of the flap residue Ile50 (Fig. 4, Fig. S5). The multipolar interactions of fluorine with the Arg8′ sidechain likely stabilize the salt-bridge with Asp29′. Moreover, the P1 difluorophenyl ring is engaged in extensive hydrophobic interactions with Pro81′, Val82′, and Ile84′ residues in the S1 subsite (Fig. 5, Fig. S6). These additional multipolar and van der Waals interactions mediated by the P1 (3,5-difluorophenyl)methyl moiety likely contributed to the improved inhibitory activity of compounds **5**–**10** against highly drug-resistant HIV-1 protease variants compared to the parent compounds (**1**–**4**).

As observed for the compounds with the P2′ 4-(1-hydroxyethyl) benzene (**5**–**10**), the 1-indanol analogs (**15**–**18**) containing a P1 (3,5-difluorophenyl)methyl moiety formed enhanced polar and hydrophobic interactions within the S1 binding pocket of protease compared to their non-fluorinated counterparts (Figure S5 and Figure S6), likely contributing toward their enhanced potency against drug-resistant variants.

Overall, the cocrystal structures of the new compounds revealed enhanced van der Waals interactions with the protease active site residues. Specifically, compounds with the P1 (3,5-difluorophenyl)methyl moiety (**5**–**10** and **15**–**18**) showed improved van der Waals interactions with residues in the S1 subsite of HIV-1 protease as compared to the corresponding non-fluorinated analogs (Fig. 5). The fluorine atoms on the P1 phenyl ring stabilize multiple protease regions, particularly 80’s loop (prime side) through interactions with Pro81′, Val82′, Ile84′, the flap region via Ile50 contacts, and the S1 subsite through van der Waals contacts with Gly27 and Asp25′ (Fig. S6). In addition to multipolar interactions, the enhanced van der Waals contacts effectively constrain the conformational flexibility of the P1 difluorophenylmethyl moiety compared to the phenylmethyl, contributing to the improved inhibitory potency of the P1 fluorinated analogs. The structural analysis further highlights the critical role of P1 fluorination in enhancing inhibitor binding through an extensive network of multipolar interactions and van der Waals contacts with key active site residues.

## 3. Conclusions

A structure-guided design strategy was employed to develop HIV-1 protease inhibitors featuring either a flexible or a conformationally constrained secondary hydroxyl group at the P2′ position by incorporating stereoisomers of the 4-(1-hydroxyethyl)benzene and 1-indanol moieties, in combination with a difluorophenyl group at the P1 position. The resulting compounds exhibited distinct potency profiles depending on the P1 fluorination, size of the P1′ hydrophobic group, and the flexibility of the P2′ moiety. In both series, P1 fluorination significantly improved potency against highly drug-resistant variants, and increasing the hydrophobic bulk at the P1′ position was generally beneficial for maintaining potency. While the stereochemistry of the 4-(1-hydroxyethyl)benzene and 1-indanol moieties had a minimal impact on both enzymatic and antiviral activity, the conformational flexibility of the P2′ hydroxyl groups appears to contribute significantly toward improving potency against highly drug-resistant variants. The cocrystal structures with protease revealed that compounds with both the 4-(1-hydroxyethyl)benzene and 1-indanol moieties make similar polar interactions in the S2′ subsite, irrespective of the configuration of the hydroxyl group. Notably, stereoisomers of the 1-indanol moiety, with a conformationally constrained hydroxyl group, adopt different conformations, with the (*R*)-1-indanol moiety flipped relative to the conformation of the (*S*)-1-indanol. The enhanced van der Waals and fluorine-mediated interactions of P1 fluorinated compounds, particularly with invariants residues in the protease active site, likely underlie their improved potency profile. Overall, our SAR results demonstrate that concurrent modifications of inhibitor moieties to enhance polar and hydrophobic interactions and optimize physicochemical properties improved potency against highly drug-resistant HIV-1 variants, providing promising lead compounds for further optimization.

## 4. Experimental section

### 4.1. Chemistry

#### General.

All chemicals, reagents and solvents were purchased from commercial sources (Sigma-Aldrich, Fisher Scientific, Chem-Impex, Biosynth) and were used as received, unless otherwise stated. Anhydrous solvents were purchased from Sigma-Aldrich and were further dried over activated 3 Å molecular sieves. All reactions were performed in oven-dried round bottomed flasks fitted with rubber septa under argon atmosphere, unless otherwise noted. Automated flash column chromatography was performed on an Interchim PuriFlash system equipped with a UV–vis detectors using prepacked silica gel cartridges or was performed manually using silica gel (230–400 mesh, EMD Millipore). Analytical thin-layer chromatography (TLC) was performed using silica gel (60 F_254_) coated aluminum plates (EMD Millipore), and spots were visualized by exposure to ultraviolet light (UV), and/or stained with iodine adsorbed on silica gel. ^1^H NMR, ^13^C NMR, ^19^F NMR spectra were acquired on a Bruker Avance III HD 500 MHz NMR instrument. Chemical shifts are reported in ppm (*δ* scale) relative to the solvent signal and coupling constant (*J*) values are reported in hertz. Data are presented as follows: chemical shift, multiplicity (s = singlet, d = doublet, t = triplet, q = quartet, m = multiplet, br = broad, dd = doublet of doublets), coupling constant in Hz, and integration. Low-resolution mass spectra (MS) were recorded on an Advion CMS using Atmospheric Pressure Chemical Ionization (APCI) in the positive mode. High-Resolution Mass Spectra (HRMS) were recorded on a Thermo Scientific Orbitrap Velos Pro mass spectrometer coupled with a Thermo Scientific Accela 1250 UPLC and an autosampler using electrospray ionization (ESI) in the positive mode. Analytical HPLC was performed on an Agilent 6130 LC/MS system equipped with an autosampler under the following conditions: column, Phenomenex Synergi Fusion-RP-C18 (4 μm, 4.6 mm × 150 mm, 80 Å); solvent A, H_2_O containing 0.1 % TFA; solvent B, CH_3_CN containing 0.1 % TFA; gradient, 20 % B to 100 % B over 15 min followed by 100 % B over 5 min; injection volume, 20 μL; flow rate, 1 mL/min. The wavelengths of detection were 254 nm and 280 nm. Retention times and purity data for each target compound are provided in the Experimental Section and Supporting Information.

### 4.2. Synthesis of HIV-1 protease inhibitors

We describe the synthesis of target compounds **5**–**6** and **15**–**16** here in detail. Synthesis details and characterization data of remaining target compounds are provided in the Supporting Information.

#### 4.2.1. tert-Butyl ((2S,3R)-1-(3,5-difluorophenyl)-3-hydroxy-4-(isobutylamino)butan-2-yl)carbamate (22a)

A solution of chiral epoxide *tert*-butyl ((*S*)-2-(3,5-difluorophenyl)-1-((*S*)-oxiran-2-yl)ethyl)carbamate **20** (0.40 g, 1.34 mmol) in EtOH (10 mL) was treated with isobutylamine (0.2 mL, 2.0 mmol) at room temperature. The resulting reaction mixture was heated at 80 °C for 3 h, cooled to room temperature, and the solvents were evaporated under reduced pressure. The residue was purified by recrystallization from a mixture of EtOAc/hexanes (1:9) (15 mL) to provide the amino alcohol **22a** (0.45 g, 90 %) as a white solid. ^1^H NMR (500 MHz, CDCl_3_) *δ* 6.78–6.75 (m, 2H), 6.67–6.63 (m, 1H), 4.69 (d, *J* = 9.2 Hz, 1H), 3.80–3.75 (m, 1H), 3.50–3.46 (m, 1H), 3.02 (dd, *J* = 4.2, 14.1 Hz, 1H), 2.81–2.76 (m, 4H), 2.49–2.41 (m, 2H), 1.80–1.72 (m, 1H), 1.36 (s, 9H), 0.92 (d, *J* = 6.6 Hz, 6H); ^13^C NMR (126 MHz, CDCl_3_) *δ* 163.91 (d, *J* = 13.1 Hz), 161.94 (d, *J* = 13.0 Hz), 155.80, 142.28 (t, *J* = 8.9 Hz), 112.51 (d, *J* = 5.6 Hz), 112.36 (d, *J* = 5.5 Hz), 101.84 (t, *J* = 25.1 Hz), 79.77, 70.60, 57.80, 53.95, 51.51, 36.63, 28.23, 20.50, 20.49; ^19^F NMR (470 MHz, CDCl_3_) *δ* −110.58 ppm; MS (APCI) *m/z*: calcd for C_19_H_31_F_2_N_2_O_3_ [M + H]^+^: 373.46; found 373.29.

#### 4.2.2. tert-Butyl ((2S,3R)-4-((4-acetyl-N-isobutylphenyl)sulfonamido)-1-(3,5-difluorophenyl)-3-hydroxybutan-2-yl)carbamate (23a)

A solution of compound **22a** (0.34 g, 0.90 mmol) in EtOAc (8 mL) at room temperature was treated with a solution of Na_2_CO_3_ (0.19 g, 1.80 mmol) in H_2_O (8 mL) followed by the addition of 4-acetylbenzenesulfonyl chloride (0.26 g, 1.18 mmol). The resulting biphasic reaction mixture was stirred at room temperature overnight and then diluted with EtOAc (50 mL). The layers were separated, and the aqueous layer was further extracted with EtOAc (50 mL). The combined organic fractions were washed with saturated aqueous NaCl solution (100 mL), dried over anhydrous Na_2_SO_4_, filtered, and then concentrated under reduced pressure. The residue obtained was purified by automated flash column chromatography using a silica gel column (SiliaSep 25 g, gradient elution with 0–50 % EtOAc/hexanes) to give compound **23a** (0.40 g, 80 %) as a white solid. ^1^H NMR (500 MHz, CDCl_3_) *δ* 8.08 (d, *J* = 8.5 Hz, 2H), 7.88 (d, *J* = 8.5 Hz, 2H), 6.80–6.76 (m, 2H), 6.68–6.64 (m, 1H), 4.65 (d, *J* = 8.3 Hz, 1H), 3.83 (s, 1H), 3.72–3.67 (m, 1H), 3.17–3.07 (m, 2H), 3.02–2.96 (m, 2H), 2.92–2.87 (m, 2H), 2.65 (s, 3H), 1.90–1.82 (m, 1H), 1.36 (s, 9H), 0.90 (d, *J* = 6.6 Hz, 3H), 0.87 (d, *J* = 6.6 Hz, 3H); ^13^C NMR (126 MHz, CDCl_3_) *δ* 196.63, 163.97 (d, *J* = 12.7 Hz), 163.00 (d, *J* = 13.1 Hz), 156.02, 142.27, 142.02 (t, *J* = 9.0 Hz), 140.15, 129.02, 127.63, 112.54 (d, *J* = 5.6 Hz), 112.39 (d, *J* = 5.5 Hz), 102.04 (t, *J* = 25.2 Hz), 80.22, 72.73, 58.63, 54.60, 53.52, 35.02, 28.20, 27.19, 26.86, 20.05, 19.85; ^19^F NMR (470 MHz, CDCl_3_) *δ* −110.16 ppm; MS (APCI) *m/z*: calcd for C_27_H_37_F_2_N_2_O_6_S [M + H]^+^: 555.56; found 555.85.

#### 4.2.3. tert-Butyl ((2S,3R)-1-(3,5-difluorophenyl)-3-hydroxy-4-((4-((S)-1-hydroxyethyl)-N-isobutylphenyl)sulfonamido)butan-2-yl)carbamate (24a)

A solution of **23a** (0.15 g, 0.27 mmol) in anhydrous THF (6 mL) under argon was cooled to 0 °C, and then the chiral catalyst (*R*)-2-methyl-CBS-oxazaborolidine (0.15 g, 0.54 mmol) was added. After stirring the reaction mixture at 0 °C for 15 min, a solution of BH_3_-THF complex (1 M in THF) (0.46 mL, 0.40 mmol) was added dropwise over 1 h. Stirring was continued at 0 °C until the reaction was complete, as monitored by TLC. The reaction was quenched with a mixture of acetone/methanol (1:1, 10 mL) and the solvents were evaporated under reduced pressure. The crude product was purified by automated flash chromatography using a silica gel column (SiliaSep 25 g, gradient elution with 0–60 % EtOAc/hexanes) to give compound **24a** as a white solid (0.15 g, 96 %). ^1^H NMR (500 MHz, CDCl_3_) *δ* 7.75 (d, *J* = 8.4 Hz, 2H), 7.53 (d, *J* = 8.2 Hz, 2H), 6.79–6.77 (m, 2H), 6.67–6.63 (m, 1H), 4.98 (q, *J* = 6.4 Hz, 1H), 4.67 (d, *J* = 8.5 Hz, 1H), 3.82–3.81 (m, 1H), 3.72–3.66 (m, 1H), 3.10–3.06 (m, 2H), 3.02–2.94 (m, 2H), 2.90–2.83 (m, 2H), 1.89–1.81 (m, 2H), 1.51 (d, *J* = 6.5 Hz, 3H), 1.35 (s, 9H), 0.91 (d, *J* = 6.5 Hz, 3H), 0.88 (d, *J* = 6.6 Hz, 3H); ^13^C NMR (126 MHz, CDCl_3_) *δ* 163.94 (d, *J* = 12.9 Hz), 161.97 (d, *J* = 12.7 Hz), 155.94, 151.16, 142.16 (t, *J* = 8.6 Hz), 136.92, 127.61, 126.16, 112.57 (d, *J* = 5.6 Hz), 112.42 (d, *J* = 5.5 Hz), 101.96 (t, *J* = 25.1 Hz), 80.08, 72.90, 69.63, 58.97, 54.47, 53.83, 35.08, 28.20, 27.27, 25.44, 20.11, 19.87; ^19^F NMR (470 MHz, CDCl_3_) *δ* −110.29 ppm; MS (APCI) *m/z*: calcd for C_27_H_39_F_2_N_2_O_6_S [M + H]^+^: 557.67; found 557.89.

#### 4.2.4. tert-Butyl ((2S,3R)-1-(3,5-difluorophenyl)-3-hydroxy-4-((4-((R)-1-hydroxyethyl)-N-isobutylphenyl)sulfonamido)butan-2-yl)carbamate (26a)

The same procedure was used as described above for compound **24a**. A solution of **23a** (0.49 g, 0.88 mmol) in anhydrous THF (20 mL) was treated with (*S*)-2-methyl-CBS-oxazaborolidine (0.49 g, 1.76 mmol) catalyst and a solution of BH_3_-THF complex (1 M in THF) (1.32 mL, 1.32 mmol) to give compound **26a** as a white solid (0.48 g, 98 %). ^1^H NMR (500 MHz, CDCl_3_) *δ* 7.75 (d, *J* = 8.4 Hz, 2H), 7.53 (d, *J* = 8.3 Hz, 2H), 6.80–6.77 (m, 2H), 6.67–6.63 (m, 1H), 4.98 (q, *J* = 6.4 Hz, 1H), 4.67 (d, *J* = 8.6 Hz, 1H), 4.02 (s, 1H), 3.81 (br s, 1H), 3.69 (sept, *J* = 4.6, 1H), 3.07–3.06 (m, 2H), 3.02–2.94 (m, 2H), 2.90–2.82 (m, 2H), 2.08 (br s, 1H), 1.89–1.80 (m, 1H), 1.50 (d, *J* = 6.5 Hz, 3H), 1.35 (s, 9H), 0.91 (d, *J* = 6.5 Hz, 3H), 0.88 (d, *J* = 6.5 Hz, 3H); ^13^C NMR (126 MHz, CDCl_3_) *δ* 163.94 (d, *J* = 12.8 Hz), 161.92 (d, *J* = 12.8 Hz), 155.95, 151.17, 142.16 (t, *J* = 9.1 Hz), 136.90, 127.61, 126.16, 112.58 (d, *J* = 5.5 Hz), 112.42 (d, *J* = 5.4 Hz), 101.95 (t, *J* = 25.2 Hz), 80.08, 72.90, 69.63, 58.97, 54.47, 53.83, 35.09, 28.20, 27.27, 25.45, 20.10, 19.87; ^19^F NMR (470 MHz, CDCl_3_) *δ* −110.30 ppm; MS (APCI) *m/z*: calcd for C_27_H_39_F_2_N_2_O_6_S [M + H]^+^: 557.67; found 557.27.

#### 4.2.5. (3R,3aS,6aR)-Hexahydrofuro[2,3-b]furan-3-yl ((2S,3R)-1-(3,5-difluorophenyl)-3-hydroxy-4-((4-((S)-1-hydroxyethyl)-N-isobutylphenyl) sulfonamido)butan-2-yl)carbamate (5)

A solution of compound **24a** (0.28 g, 0.50 mmol) in anhydrous CH_2_Cl_2_ (5 ml) was treated with TFA (4 mL) and the resulting reaction mixture was stirred at room temperature for 2 h. Upon completion of the reaction, solvents were evaporated under reduced pressure. Toluene (5 mL) was added, then evaporated under reduced pressure, and the residue was dried under high vacuum. A solution of the resulting amine salt in anhydrous CH_3_CN (5 mL) under argon was cooled to 0 °C and diisopropylethylamine (0.25 mL, 1.50 mmol) was added followed by *bis*-THF activated carbonate **25** (0.16 g, 0.60 mmol). After 15 min, the reaction mixture was allowed to warm to room temperature and stirred for 36 h. The solvents were evaporated under reduced pressure, and the residue was purified by automated flash chromatography using a silica gel column (SiliaSep, 25 g, gradient elution with 0–80 % EtOAc/hexanes) to give compound **5** (0.29 g, 94 %) as a white solid. ^1^H NMR (500 MHz, CDCl_3_) *δ* 7.73 (d, *J* = 8.3 Hz, 2H), 7.52 (d, *J* = 8.2 Hz, 2H), 6.76–6.75 (m, 2H), 6.66–6.62 (m, 1H), 5.64 (d, *J* = 5.2 Hz, 1H), 5.26 (d, *J* = 9.1 Hz, 1H), 5.03–4.99 (m, 1H), 4.98–4.94 (m, 1H), 3.93–3.81 (m, 4H), 3.72–3.67 (m, 3H), 3.12–3.03 (m, 3H), 2.97–2.85 (m, 3H), 2.74 (dd, *J* = 13.9, 9.9 Hz, 1H), 2.48 (d, *J* = 3.0 Hz, 1H), 1.88–1.80 (m, 1H), 1.73-1.65 (m, 1H), 1.54–1.52 (m, 1H), 1.49 (d, *J* = 6.5 Hz, 3H), 0.90 (dd, *J* = 11.2, 6.5 Hz, 6H); ^13^C NMR (126 MHz, CDCl_3_) *δ* 163.97 (d, *J* = 12.6 Hz), 161.99 (d, *J* = 12.8 Hz), 155.38, 151.38, 142.00 (t, *J* = 9.2 Hz), 136.68, 127.59, 126.26, 112.47 (d, *J* = 5.5 Hz), 112.32 (d, *J* = 5.4 Hz), 109.31, 102.14 (t, *J* = 25.0 Hz), 73.63, 72.80, 70.86, 69.57, 69.52, 59.05, 54.85, 53.78, 45.38, 35.47, 27.34, 25.76, 25.42, 20.12, 19.88; ^19^F NMR (470 MHz, CDCl_3_) *δ* −109.97 ppm; HRMS (ESI) *m/z*: calcd for C_29_H_39_F_2_N_2_O_8_S [M + H]^+^: 613.2390; found 613.2388. Anal. HPLC: *t*_R_ 10.28 min, purity 100 %.

#### 4.2.6. (3R,3aS,6aR)-Hexahydrofuro[2,3-b]furan-3-yl ((2S,3R)-1-(3,5-difluorophenyl)-3-hydroxy-4-((4-((R)-1-hydroxyethyl)-N-isobutylphenyl) sulfonamido)butan-2-yl)carbamate (6)

The same procedure was used as described above for compound **5**. A solution of compound **26a** (0.25 g, 0.45 mmol) in CH_2_Cl_2_ (4 ml) was treated with TFA (3 mL). The resulting amine salt was dissolved in anhydrous CH_3_CN (5 mL) and treated with diisopropylethylamine (0.23 g, 1.80 mmol) and *bis*-THF activated carbonate **25** (0.15 g, 0.54 mmol) to give compound **6** (0.24 g, 87 %) as a white solid. ^1^H NMR (500 MHz, CDCl_3_) *δ* 7.76 (d, *J* = 8.3 Hz, 2H), 7.55 (d, *J* = 8.2 Hz, 2H), 6.78–6.74 (m, 2H), 6.68–6.64 (m, 1H), 5.66 (d, *J* = 5.1 Hz, 1H), 5.05–4.95 (m, 3H), 3.97–3.89 (m, 2H), 3.86–3.81 (m, 2H), 3.78–3.73 (m, 1H), 3.69–3.66 (m, 2H), 3.15–2.92 (m, 5H), 2.86–2.76 (m, 2H), 2.04 (d, *J* = 3.4 Hz, 1H), 1.86–1.78 (m, 1H), 1.76–1.70 (m, 1H), 1.64–1.60 (m, 1H), 1.52 (d, *J* = 6.5 Hz, 3H), 0.94 (d, *J* = 6.6 Hz, 3H), 0.90 (d, *J* = 6.5 Hz, 3H); ^13^C NMR (126 MHz, DMSO-*d*_6_) *δ* 163.52, 161.52 (d, *J* = 13.6 Hz), 155.63, 152.81, 144.75, 137.21, 127.47, 126.46, 112.48 (d, *J* = 5.5 Hz), 112.33 (d, *J* = 5.4 Hz), 109.28, 101.80 (t, *J* = 25.1), 72.98, 70.91, 69.11, 67.97, 57.66, 55.84, 52.98, 45.60, 35.19, 26.75, 26.17, 25.89, 20.41, 20.37; ^19^F NMR (470 MHz, CDCl_3_) *δ* −109.73 ppm; HRMS (ESI) *m/z*: calcd for C_29_H_39_F_2_N_2_O_8_S [M + H]^+^: 613.2390; found 613.2388. Anal. HPLC: *t*_R_ 10.33 min, purity 98 %.

#### 4.2.7. tert-Butyl ((2S,3R)-1-(3,5-difluorophenyl)-3-hydroxy-4-((N-isobutyl-1-oxo-2,3-dihydro-1H-indene)-5-sulfonamido)butan-2-yl) carbamate (28a)

A solution of compound **22a** (0.39 g, 1.04 mmol) in EtOAc (5 mL) was treated with a solution of Na_2_CO_3_ (0.19 g, 1.77 mmol) in H_2_O (5 mL) followed by the addition of 5-indanonesulfonyl chloride (0.25 g, 1.09 mmol). The resulting biphasic reaction mixture was stirred at room temperature for 14 h. The reaction mixture was diluted with EtOAc (50 mL), and the layers were separated. The aqueous layer was further extracted with EtOAc (2 × 50 mL). The combined organic portions were washed with saturated aqueous NaCl solution (50 mL), dried (Na_2_SO_4_), filtered, and concentrated under reduced pressure. The residue was purified by automated flash column chromatography (RediSep Gold, 24 g, gradient elution with 0–80 % EtOAc/hexanes), to give compound **28a** (0.53 g, 90 %) as a white solid. ^1^H NMR (500 MHz, CDCl_3_) *δ* 7.92 (br s, 1H), 7.88 (d, *J* = 8.0 Hz, 1H), 7.77 (dd, *J* = 8.0, 1.0 Hz, 1H), 6.80–6.77 (m, 2H), 6.67 (tt, *J* = 9.0, 2.5 Hz, 1H), 4.64 (d, *J* = 8.5 Hz, 1H), 4.00 (br s, 1H), 3.88–3.79 (m, 1H), 3.73–3.67 (m, 1H), 3.25 (t, *J* = 5.5 Hz, 2H), 3.18 (dd, *J* = 15.0, 4.0 Hz, 1H), 3.12 (dd, *J* = 15.5, 8.0 Hz, 1H), 3.03–2.98 (m, 2H), 2.95–2.87 (m, 2H), 2.80–2.78 (m, 2H), 1.88 (sep, *J* = 7.0 Hz, 1H), 1.36 (s, 9H), 0.92 (d, *J* = 6.5 Hz, 3H), 0.88 (d, *J* = 6.5 Hz, 3H) ppm; ^13^C NMR (126 MHz, CDCl_3_) *δ* 205.46, 164.11 (d, *J* = 13.0 Hz), 162.14 (d, *J* = 12.7 Hz), 156.21, 155.63, 144.05, 142.16 (t, *J* = 9.3 Hz), 140.32, 126.37, 126.05, 124.78, 112.55 (d, *J* = 5.6 Hz), 112.40 (d, *J* = 5.6 Hz), 102.20 (t, *J* = 25.2 Hz), 80.39, 72.92, 58.84, 54.75, 53.69, 36.58, 35.12, 28.37, 27.37, 26.09, 20.21, 20.00 ppm; ^19^F NMR (470 MHz, CDCl_3_) *δ* −110.14 ppm.

#### 4.2.8. (3R,3aS,6aR)-Hexahydrofuro[2,3-b]furan-3-yl ((2S,3R)-1-(3,5-difluorophenyl)-3-hydroxy-4-((N-isobutyl-1-oxo-2,3-dihydro-1H-indene)-5-sulfonamido)butan-2-yl)carbamate (30a)

A solution of compound **28a** (0.52 g, 0.92 mmol) in anhydrous CH_2_Cl_2_ (8 mL) was treated with TFA (6 mL). After stirring the reaction mixture at the room temperature for 2 h, the solvents were evaporated under reduced pressure. Toluene (6 mL) was added, then evaporated under reduced pressure, and the residue was dried under high vacuum. A solution of the resulting amine salt was in anhydrous CH_3_CN (20 mL) was cooled to 0 °C under argon and treated with diisopropylethylamine (0.36 g, 2.76 mmol) followed by *bis*-THF activated carbonate **25** (0.30 g, 1.10 mmol). After 15 min, the reaction mixture was allowed to warm to room temperature and stirred for 36 h. The solvents were evaporated under reduced pressure, and the residue was purified by automated flash column chromatography using a silica gel column (RediSep Gold, 24 g, gradient elution with 0–10 % methanol/CH_2_Cl_2_) to give compound **30a** (0.45 g, 78 %) as a white solid. ^1^H NMR (500 MHz, CDCl_3_) *δ* 7.93 (br s, 1H), 7.88 (d, *J* = 8.0 Hz, 1H), 7.77 (d, *J* = 8.0 Hz, 1H), 6.80–6.74 (m, 2H), 6.67 (tt, *J* = 9.0, 1.5 Hz, 1H), 5.66 (d, *J* = 5.0 Hz, 1H), 5.09 (d, *J* = 8.5 Hz, 1H), 5.05 (q, *J* = 8.0 Hz, 1H), 3.95 (dd, *J* = 10.0, 6.0 Hz, 1H), 3.92–3.82 (m, 3H), 3.78–3.70 (m, 2H), 3.66 (br s, 1H), 3.25 (t, *J* = 5.5 Hz, 2H), 3.19 (dd, *J* = 15.5, 8.0 Hz, 1H), 3.14-3.00 (m, 3H), 2.97–2.89 (m, 2H), 2.81–2.76 (m, 3H), 1.86 (sep, *J* = 7.0 Hz, 1H), 1.78–1.68 (m, 1H), 1.60–1.55 (m, 1H), 0.93 (d, *J* = 6.5 Hz, 3H), 0.89 (d, *J* = 6.5 Hz, 3H) ppm; ^13^C NMR (126 MHz, CDCl_3_) *δ* 205.41, 164.13 (d, *J* = 13.0 Hz), 162.15 (d, *J* = 12.9 Hz), 155.68, 155.58, 143.87, 141.90 (t, *J* = 9.0 Hz), 140.41, 126.35, 126.04, 124.83, 112.45 (d, *J* = 5.5 Hz), 112.30 (d, *J* = 5.6 Hz), 109.44, 102.34 (t, *J* = 25.0 Hz), 73.86, 72.89, 70.96, 69.63, 58.92, 55.04, 53.67, 45.58, 36.58, 35.42, 27.45, 26.10, 25.90, 20.23, 19.99 ppm; ^19^F NMR (470 MHz, CDCl_3_) *δ* −109.65 ppm; MS (APCI) *m/z*: calcd for C_30_H_37_F_2_N_2_O_8_S [M + H]^+^: 623.22; found 623.25.

#### 4.2.9. (3R,3aS,6aR)-Hexahydrofuro[2,3-b]furan-3-yl ((2S,3R)-1-(3,5-difluorophenyl)-3-hydroxy-4-(((S)-1-hydroxy-N-isobutyl-2,3-dihydro-1H-indene)-5-sulfonamido)butan-2-yl)carbamate (15)

A solution of compound **30a** (0.15 g, 0.24 mmol) in anhydrous CH_2_Cl_2_ (5 mL) under argon was cooled to 0 °C and treated slowly with a mixture of HCO_2_H and Et_3_N (1:2 ratio, 15 mL) over 10 min. The reaction mixture was allowed to warm to room temperature and stirred for 30 min. Then, Noyori asymmetric transfer hydrogenation catalyst RuCl[(*S*, *S*)-Ts-DPEN](mesitylene) (5 mol%) (7.5 mg, 0.012 mmol) was added and the reaction mixture was stirred at room temperature for 72 h. The reaction was then quenched with saturated aqueous NaHCO_3_ solution and extracted with CH_2_Cl_2_ (3 × 30 mL). The combined organic portions were dried (Na_2_SO_4_), filtered, and concentrated under reduced pressure. The residue was purified by automated flash column chromatography using a silica gel column (RediSep Gold, 24 g, gradient elution with 0–10 % methanol/CH_2_Cl_2_) to provide the target compound **15** (0.11 g, 73 %) as a white solid. ^1^H NMR (500 MHz, CDCl_3_) *δ* 7.68–7.63 (m, 2H), 7.56 (d, *J* = 7.5 Hz, 1H), 6.78–6.74 (m, 2H), 6.68–6.64 (m, 1H), 5.66 (d, *J* = 5.5 Hz, 1H), 5.29 (t, *J* = 6.5 Hz, 1H), 5.05–5.00 (m, 2H), 3.95 (dd, *J* = 9.5, 5.0 Hz, 1H), 3.90 (td, *J* = 8.5, 2.0 Hz, 1H), 3.85–3.82 (m, 2H), 3.77–3.72 (m, 1H), 3.69 (dd, *J* = 9.5, 6.0 Hz, 1H), 3.15–2.98 (m, 5H), 2.96–2.82 (m, 3H), 2.78 (dd, *J* = 14.0, 9.5 Hz, 1H), 2.62–2.55 (m, 1H), 2.05–1.96 (m, 1H), 1.84 (sep, *J* = 7.0 Hz, 1H), 1.78–1.68 (m, 1H), 1.61–1.57 (m, 1H), 0.95 (d, *J* = 6.5 Hz, 3H), 0.91 (d, *J* = 6.5 Hz, 3H) ppm; ^13^C NMR (126 MHz, CDCl_3_) *δ* 164.11 (d, *J* = 12.6 Hz), 162.13 (d, *J* = 12.7 Hz), 155.51, 150.54, 144.87, 141.99 (t, *J* = 9.0 Hz), 137.90, 126.35, 125.19, 123.97, 112.47 (d, *J* = 5.9 Hz), 112.32 (d, *J* = 5.5 Hz), 109.44, 102.28 (t, *J* = 24.7 Hz), 75.83, 73.77, 72.98, 70.95, 69.65, 59.32, 54.99, 54.00, 45.50, 36.22, 35.60, 29.88, 27.54, 25.88, 20.29, 20.03 ppm; ^19^F NMR (470 MHz, CDCl_3_) *δ* −109.78 ppm; HRMS (ESI) *m/z*: calcd for C_30_H_39_F_2_N_2_O_8_S [M + H]^+^: 625.2390; found 625.2389. Anal. HPLC: *t*_R_ 10.39 min, purity 98 %.

#### 4.2.10. (3R,3aS,6aR)-Hexahydrofuro[2,3-b]furan-3-yl ((2S,3R)-1-(3,5-difluorophenyl)-3-hydroxy-4-(((R)-1-hydroxy-N-isobutyl-2,3-dihydro-1H-indene)-5-sulfonamido)butan-2-yl)carbamate (16)

The same procedure was used as described above for compound **15**. A solution of compound **30a** (0.15 g, 0.24 mmol) in anhydrous CH_2_Cl_2_ (5 mL) was treated with a mixture of HCO_2_H and Et_3_N (1:2 ratio, 15 mL) followed by the addition of Noyori asymmetric transfer hydrogenation catalyst RuCl[(*R*,*R*)-TsDPEN](mesitylene) (5 mol%) (7.50 mg, 0.012 mmol) to provide the target compound **16** (0.12 g, 80 %) as a white solid. ^1^H NMR (500 MHz, CDCl_3_) *δ* 7.67–7.63 (m, 2H), 7.56 (d, *J* = 7.5 Hz, 1H), 6.78–6.73 (m, 2H), 6.66 (t, *J* = 9.0 Hz, 1H), 5.66 (d, *J* = 5.5 Hz, 1H), 5.29 (t, *J* = 6.5 Hz, 1H), 5.05–5.02 (m, 2H), 3.96 (dd, *J* = 9.5, 6.0 Hz, 1H), 3.90 (td, *J* = 8.0, 1.5 Hz, 1H), 3.88–3.81 (m, 2H), 3.76–3.67 (m, 3H), 3.16–2.98 (m, 5H), 2.96–2.74 (m, 4H), 2.62–2.55 (m, 1H), 2.05–1.97 (m, 1H), 1.84 (sep, *J* = 7.0 Hz, 1H), 1.78–1.68 (m, 1H), 1.60–1.56 (m, 1H), 0.95 (d, *J* = 6.5 Hz, 3H), 0.91 (d, *J* = 7.0 Hz, 3H) ppm; ^13^C NMR (126 MHz, CDCl_3_) *δ* 164.12 (d, *J* = 12.7 Hz), 162.14 (d, *J* = 12.7 Hz), 155.51, 150.53, 144.87, 141.98 (t, *J* = 9.0 Hz), 137.94, 126.36, 125.20, 123.97, 112.48 (d, *J* = 5.5 Hz), 112.33 (d, *J* = 5.7 Hz), 109.43, 102.30 (t, *J* = 25.4 Hz), 75.84, 73.78, 72.97, 70.91, 69.65, 59.34, 54.99, 54.02, 45.50, 36.26, 35.56, 29.89, 27.57, 25.88, 20.30, 20.03 ppm; ^19^F NMR (470 MHz, CDCl_3_) *δ* −109.76 ppm; HRMS (ESI) *m/z*: calcd for C_30_H_39_F_2_N_2_O_8_S [M + H]^+^: 625.2390; found 625.2386. Anal. HPLC: *t*_R_ 10.31 min, purity 98 %.

## 5. Biology

### 5.1. Protein expression and purification

The expression, isolation, and purification of drug resistant HIV-1 proteases used for the enzyme inhibition assays and the wild-type HIV-1 protease used for crystallization were carried out as previously described [32,33]. Briefly, the genes encoding the HIV protease were subcloned into the heat-inducible pXC35 expression vector (ATCC) and transformed into *E. coli* TAP-106 cells. Cells grown in 6 L of Terrific Broth were lysed with a cell disruptor and the protein was purified from inclusion bodies [34]. The inclusion body centrifugation pellet was dissolved in 50 % acetic acid followed by another round of centrifugation to remove impurities. Size-exclusion chromatography was used to separate high molecular weight proteins from the desired protease. This was carried out on a 2.1 L Sephadex G-75 superfine column (Millipore Sigma) equilibrated with 50 % acetic acid. The cleanest fractions of HIV protease were refolded into a 10-fold dilution of 0.05 M sodium acetate at pH 5.5, 5 % ethylene glycol, 10 % glycerol, and 5 mM DTT. Folded protein was concentrated down to 1–2 mg/mL and stored. This stored protease was used in *K*i assays. For crystallography, a final purification was performed with a Pharmacia Superdex 75 FPLC column equilibrated with 0.05 M sodium acetate at pH 5.5, 5 % ethylene glycol, 10 % glycerol, and 5 mM DTT. Protease fractions purified from the size exclusion column were concentrated to 1–2 mg/mL using an Amicon Ultra-15 10-kDa device (Millipore) for crystallization.

### 5.2. Enzyme inhibition assays

The enzyme inhibition assays were carried out as previously described [21,22,35,36]. To determine the enzyme inhibition constant (*K*i), in a 96-well plate, each inhibitor was serially diluted, including a no drug control, and incubated with protein (0.75 nM–5.0 nM) for 1 h. A 10-amino acid substrate containing an optimized protease cleavage site with an EDANS/DABCYL FRET pair [35] was dissolved in 4 % DMSO at 120 μM. Using the Envision plate reader, 5 μL of the 120 μM substrate was added to the 96-well plate to a final concentration of 10 μM. The fluorescence was observed with an excitation at 340 nm and emission at 492 nm and monitored for 200 counts, for approximately 60 min. Data was analyzed with GraphPad Prism (version 10). DRV was used as a control in all assays.

### 5.3. Protein crystallization

The conditions that reliably produced cocrystals of NL4-3 wild-type protease bound to PIs were discovered and optimized as previously described [21,37,38]. Briefly, all cocrystals were grown at room temperature by hanging drop vapor diffusion method in a 24-well VDX hanging-drop trays (Hampton Research) with a protease concentration of 1.0–2.0 mg/mL with 3- to 5-fold molar excess of inhibitors (0.5–1.0 % DMSO). The crystallization drops were set with 1.0 μL of protein-inhibitor solution and 2.0 μL of reservoir solution, which contained 23–24 % (w/v) ammonium sulfate with 0.1 M bis-Tris-methane buffer at pH 5.5. The small size crystals were obtained within one week which were used as micro-seeds. Crystal quality was greatly improved by micro-seeding crystallization drops containing protein-inhibitor complex to precipitant ratio of 1:2 to 3:2 (μL), with seeds at seed dilutions between 1:100 to 1:1000. Diffraction quality crystals were obtained within 1 week. Data were collected at 100 K, with cryogenic conditions maintained by adding 25 % glycerol to the precipitant solution.

### 5.4. X-ray data collection and structure solution

X-ray diffraction data were collected and solved as previously described [21,32,37]. Diffraction quality crystals were flash frozen in liquid nitrogen and X-ray diffraction data were collected at either the Chicago APS Synchrotron Beamline 23-1D-D or the Brookhaven National Laboratory NSLS II AMX and FMX beamlines. Crystallographic data was indexed, integrated, and scaled using XDS [39]. All structures were solved using molecular replacement with PHASER [40]. Model building and refinement were performed using Coot [41] and Phenix [42]. HIV inhibitors were designed in Maestro and optimized with Gaussian 16 (Revision C.01) prior to using the Phenix program eLBOW [43] to generate cif files containing atomic positions and constraints necessary for ligand refinement. Iterative rounds of crystallographic refinement were carried out until convergence was achieved. To limit bias throughout the refinement process, five percent of the data were reserved for the free R-value calculation [44]. MolProbity [45] was applied to evaluate the final structures before deposition in the PDB. Structure analysis, superposition and figure generation was done using PyMOL [46]. X-ray data collection and crystallographic refinement statistics are presented in the Supporting Information (Table S2–S4). *F*_o_–*F*_c_ electron density maps for representative inhibitors are shown in Fig. S7.

### 5.5. Intermolecular van der Waals contact analysis

To calculate the intermolecular van der Waals (vdW) interaction energies the crystal structures were prepared using the Schrödinger Protein Preparation Wizard [47]. Hydrogen atoms were added, protonation states were determined, and the structures were minimized. The protease active site was monoprotonated at Asp25. Subsequently, force field parameters were assigned using the OPLS2005 force field [48]. Interaction energies between the inhibitor and protease were estimated using a simplified Lennard–Jones potential, as previously described in detail [49,50]. Briefly, the vdW energy was calculated for pairwise interactions depending on the types of atoms interacting and the distance between them. For each protease residue, the change in vdW interactions relative to a reference complex in the same space group was also calculated for each protease-inhibitor complex.

### 5.6. Antiviral assays

293T and TZM-BL [51] cells (NIH AIDS Research and Reference Reagent Program) were maintained in Dulbecco’s modified Eagle’s medium supplemented with 10 % fetal calf serum in the presence of penicillin and streptomycin at 37 °C with 5 % CO_2_. To determine the concentration of drugs achieving 50 % inhibition of infection compared with the drug-free control, 4.5 × 10^6^ 293T cells were seeded onto a 10-cm plate 24 h before transfection. Cells were transfected with 8 μg of the wild-type plasmid, the infectious molecular clone pNL-CH derived from the pNL4-3 clone of HIV-1, using FuGENE HD transfection reagent (Roche). The culture supernatant of 293T cells transfected with wild-type or PI-resistant HIV-1 variant was removed 18 h after transfection and the cells were washed with 1 × PBS. The 293T cells were collected and transferred to wells of a 24-well plate. Briefly, each drug was serially diluted in the culture medium, and the dilutions were added to the wells of a 24-well plate. The 293T cells (0.5 × 10^6^ per well) collected from the transfection were added to wells containing various concentrations of drug. The culture supernatant containing virus particles was harvested 18 h after the 293T cells were reseeded in the presence of drug. This supernatant was filtered through a 0.45-μm-pore-size membrane (Millipore) to remove cell debris then used to infect 2 × 10^4^ TZM-BL cells in a 96-well plate following a procedure previously described [52]. The culture supernatant was removed from each well 48 h post-infection, and the cells were washed with 1 × PBS. For the luciferase assay, infected TZM-BL cells were lysed in 1 × reporter lysis buffer (Promega) and the cells were kept at −80 °C. After one freeze-thaw cycle, the cell lysates were transferred into a 96-well assay plate (Costar), and luciferase activity was measured using a luminometer (Promega). The culture supernatant harvested from 293T cells reseeded in the absence of drugs was used as a drug-free control. EC_50_ was determined based on a dose-response curve generated using GraphPad Prism (version 7.0).

### 5.7. Protein data bank accession codes

The PDB accession codes for X-ray cocrystal structures of HIV-1 protease with inhibitors: **5**, 00009YRR; **6**, 00009Q1C; **7**, 00009Q3P; **8**, 00009Q5D; **9**, 00009PZ1; **10**, 00009PYX; **11**, 00009YKP; **12**, 00009Q3T; **13**, 00009YRY; **14**, 00009YRA; **15**, 00009Q0T; **16**, 00009Q13; **18**, 00009Q1P.

## Supplementary Material

Supplementary Material

## Figures and Tables

**Table 1:** Inhibitory activity of compounds **5**–**10** against drug-resistant HIV-1 protease variants.

Inhibitor	Structure	*K*i (nM)^a^			
		I84V	I50V/A71V	10Mut-84V	10Mut-50V
**1**	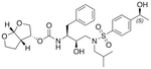	0.048 ± 0.004	0.057 ± 0.008	109 ± 10	251 ± 14
**2**	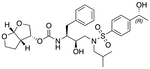	0.109 ± 0.007	0.092 ± 0.008	50.1 ± 3.0	231 ± 13
**3**	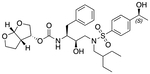	0.020 ± 0.002	0.102 ± 0.010	65.6 ± 4.0	213 ± 17
**4**	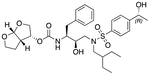	0.028 ± 0.003	0.107 ± 0.014	107 ± 14	93 ± 12
**5**	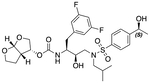	0.009 ± 0.003	0.011 ± 0.003	24.1 ± 1.6	15.0 ± 1.4
**6**	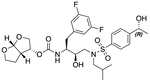	0.011 ± 0.002	0.014 ± 0.004	4.1 ± 0.2	16.3 ± 2.1
**7**	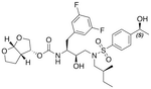	0.012 ± 0.005	<0.005	14.2 ± 0.7	17.6 ± 1.7
**8**	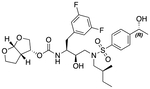	<0.005	<0.005	6.0 ± 0.9	19.9 ± 2.3
**9**	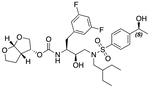	<0.005	<0.005	4.3 ± 0.4	18.0 ± 1.6
**10**	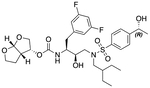	<0.005	<0.005	1.3 ± 0.2	15.0 ± 1.2
**DRV**		0.025 ± 0.006	0.075 ± 0.006	156 ± 4	55.6 ± 1.2

a The drug-resistant HIV-1 protease variants contain the following amino acid substitutions compared to the wild-type protease (NL4-3 strain): 10Mut-84V (I13V, G16E, V32I, L33F, K45I, M46I, A71V, L76V, V82F, I84V); 10Mut-50V (L10F, M46I, I47V, I50V, F53L, L63P, I72V, G73S, V82I, I85V).

**Table 2:** Inhibitory activity of compounds **11**–**18** against drug-resistant HIV-1 protease variants.

Inhibitor	Structure	*K*i (nM)^a^			
		I84V	I50V/A71V	10Mut-84V	10Mut-50V
**11**	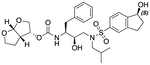	0.026 ± 0.006	0.082 ± 0.024	447 ± 24.1	155 ± 12.3
**12**	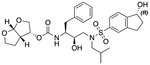	0.182 ± 0.021	0.328 ± 0.101	326 ± 27.5	854 ± 103.5
**13**	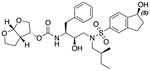	0.043 ± 0.01	0.155 ± 0.043	427 ± 21	249 ± 27.7
**14**	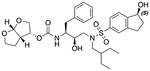	0.018 ± 0.003	0.056 ± 0.017	157 ± 12	108 ± 11.1
**15**	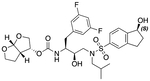	0.011 ± 0.003	0.025 ± 0.007	54.4 ± 4.7	12.8 ± 0.8
**16**	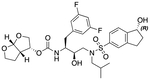	0.026 ± 0.006	0.042 ± 0.020	56.4 ± 3.1	113.3 ± 12.3
**17**	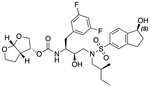	0.01 ± 0.004	<0.005	86.1 ± 5.0	40.8 ± 2.6
**18**	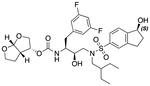	<0.005	<0.005	18.28 ± 1.4	32.7 ± 3.8
**DRV**		0.025 ± 0.006	0.075 ± 0.006	156 ± 4	55.6 ± 1.2

a The drug-resistant HIV-1 protease variants contain the following amino acid substitutions compared to the wild-type protease (NL4-3 strain): 10Mut-84V (I13V, G16E, V32I, L33F, K45I, M46I, A71V, L76V, V82F, I84V); 10Mut-50V (L10F, M46I, I47V, I50V, F53L, L63P, I72V, G73S, V82I, I85V).

**Table 3:** Antiviral activity of compounds **5**–**18** against wild-type HIV-1 and drug-resistant variants.

Inhibitor	cLogP	Antiviral EC_50_ (μM)^a (fold change)^		
		WT	HIV-1-DRV	HIV-1-V9
**5**	3.17	0.011	0.02 (<2)	0.49 (45)
**6**	3.17	0.012	0.14 (12)	1.63 (136)
**7**	3.67	0.014	0.04 (3)	0.23 (16)
**8**	3.67	0.016	<0.005 (0.3)	0.64 (40)
**9**	4.23	0.008	<0.005 (0.6)	0.64 (80)
**10**	4.23	0.018	<0.005 (0.3)	0.48 (27)
**11**	2.98	0.017	0.88 (52)	5.33 (314)
**12**	2.98	0.025	1.22 (49)	7.47 (299)
**13**	3.52	0.019	3.30 (174)	15.8 (832)
**14**	4.05	0.012	0.50 (42)	9.29 (774)
**15**	3.28	0.017	0.32 (19)	1.94 (114)
**16**	3.28	0.014	0.13 (9)	1.15 (82)
**17**	3.80	0.009	0.57 (63)	3.82 (424)
**18**	4.33	0.018	0.12 (7)	2.86 (159)
**DRV**	2.38	0.009	0.69 (77)	1.24 (138)

a The resistant HIV-1 variants contain the following amino acid substitutions in HIV-1 protease compared to the wild-type NL4-3 strain: HIV-DRV (I13V, G16E, V32I, L33F, K45I, M46I, A71V, L76V, V82F, I84V); HIV–V9 (L10F, I13V, L33F, K45R, M46I, I47V, I50V, F53L, I54L, I66F, A71V, T74A, L76S, V82I).

## Data Availability

Data will be made available on request.

## Appendix A. Supplementary data

Supplementary data to this article can be found online at https://doi.org/10.1016/j.ejmech.2025.118510.
