# Supplementary material for: Fluorinated HIV-1 protease inhibitors containing chiral hydroxyethylbenzene and indanol as P2′ ligands with potent activity against drug-resistant variants
Source: Eur J Med Chem. Author manuscript; Available in PMC 2026 Apr 18. (PMC13091712; doi:10.1016/j.ejmech.2025.118510)
Supplement: Supplementary Material [file NIHMS2164643-supplement-Supplementary_Material.pdf]

Supporting Information

Fluorinated HIV-1 Protease Inhibitors Containing Chiral Hydroxyethylbenzene and Indanol as P2′ Ligands with Potent Activity Against Drug-Resistant Variants

Jagroop Kaur,[1] Ean Spielvogel,[2,3] Desaboini Nageswara Rao,[1] Linah N. Rusere,[1] Ala M. Shaqra,[1] Gordon J. Lockbaum,[1] Arooma Maryam,[1] Nese Kurt Yilmaz,[1] Ronald Swanstrom,[2,3] Celia A. Schiffer,[1,*] Akbar Ali[1,*]

[1]Department of Biochemistry and Molecular Biotechnology, University of Massachusetts Chan Medical School, Worcester, MA 01605, United States

[2]Lineberger Comprehensive Cancer Center, University of North Carolina at Chapel Hill, Chapel Hill, NC 27599, United States

[3]Department of Biochemistry and Biophysics, University of North Carolina at Chapel Hill, Chapel Hill, NC 27599, United States

Table S1. Inhibitory activity of indanol compounds against wild-type HIV-1 protease

Inhibitor	Structure	*K*i (nM)
**11**	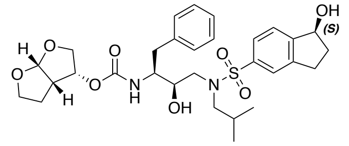	<0.005
**12**	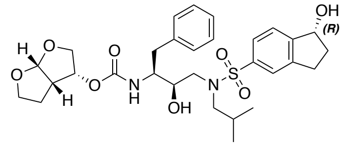	0.006 ± 0.004
**13**	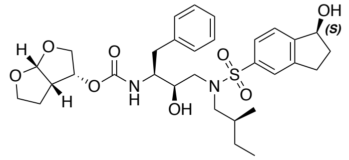	0.006 ± 0.004
**16**	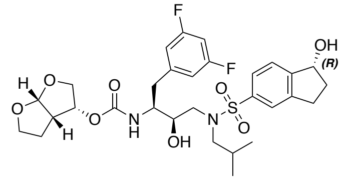	<0.005
**18**	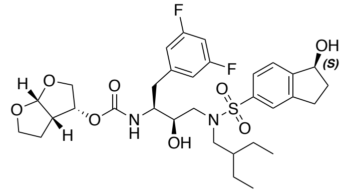	<0.005
**DRV**		0.007 ± 0.004

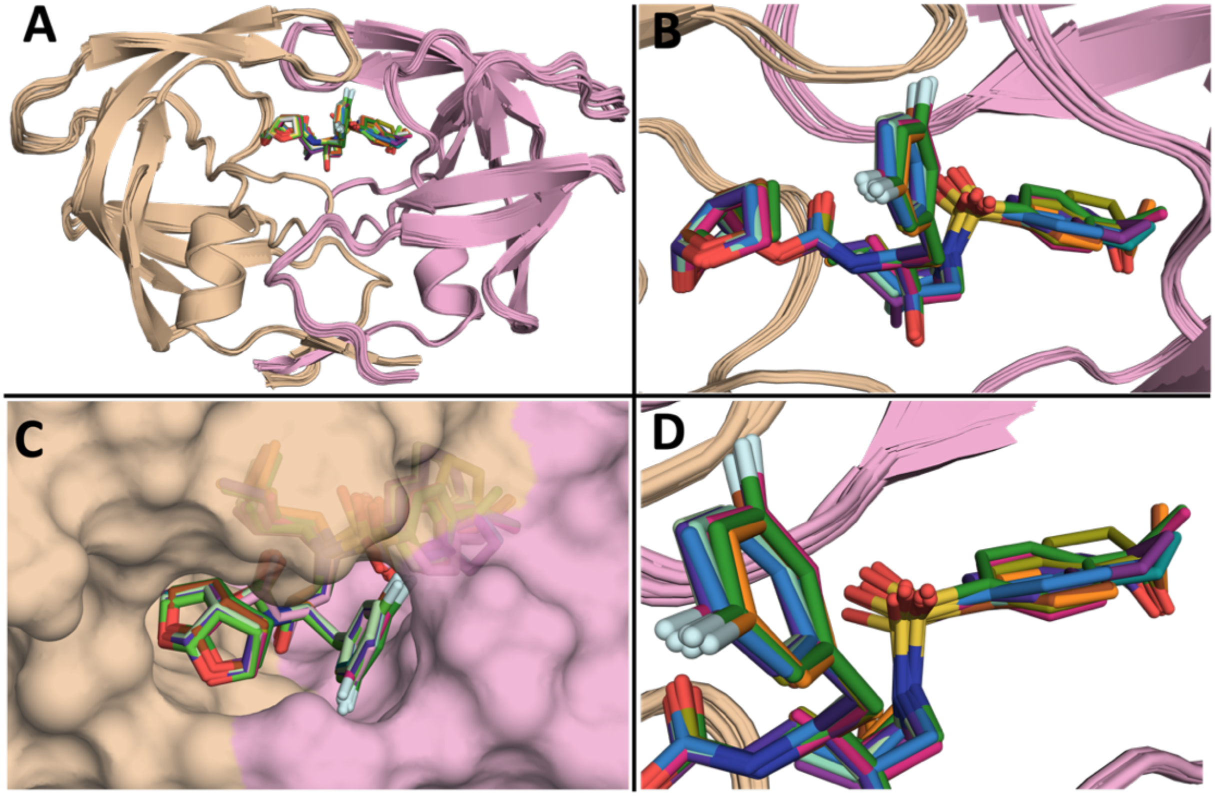

Figure S1. Binding modes of protease inhibitors in the active site of HIV-1 protease. The two protease monomers are in gold (denoted as nonprime) and pink (denoted as prime) colored cartoons. (A) Superposition of protease complexes with darunavir, parent compounds (**1**–**4**) and new analogs (**5**–**18**). The inhibitors are shown as sticks, and HIV-1 protease dimers are shown as cartoon representations. (B) Zoomed-in view of the active site, with superimposed complexes showing similar overall binding conformations of all the compounds. (C) Surface view of inhibitors bound within the active site of HIV-1 protease. (D) Variations in the binding modes of inhibitors' P1 and P2′ moieties.

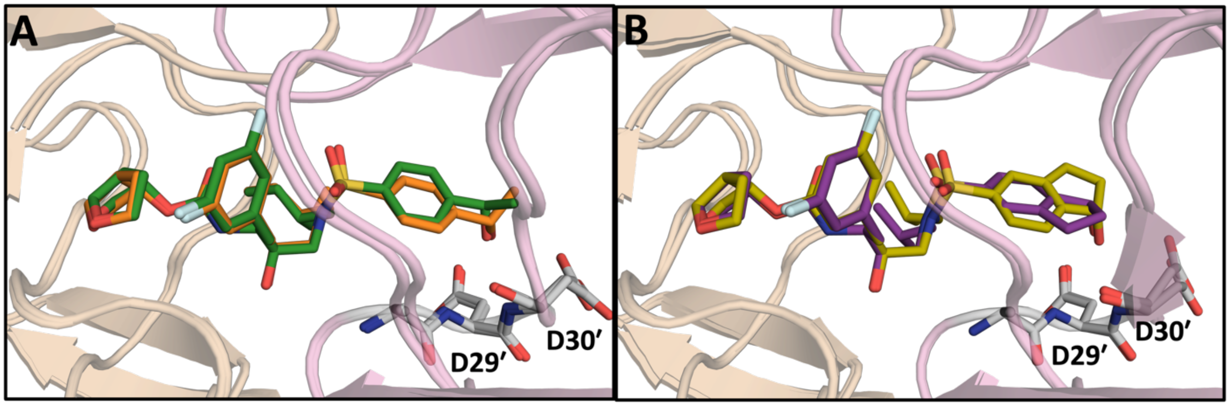

Figure S2. Superimposed cocrystal structures of P2′ epimeric compounds showing the similar orientation of the hydroxyl group toward Asp29′ and Asp30′ residues, irrespective of the stereochemistry of the P2′ hydroxyl group. (A) Superposition of cocrystal structures of compounds **9** (orange) and **10** (green) highlighting the differences in binding conformations of the P2′ (*S*)- and (*R*)-4-(1-hydroxyethyl)benzene moieties. (B) **15** (deep olive) and **16** (purple) highlighting the differences in binding conformations of the P2′ (*S*)- and (*R*)-1-indanol moieties.

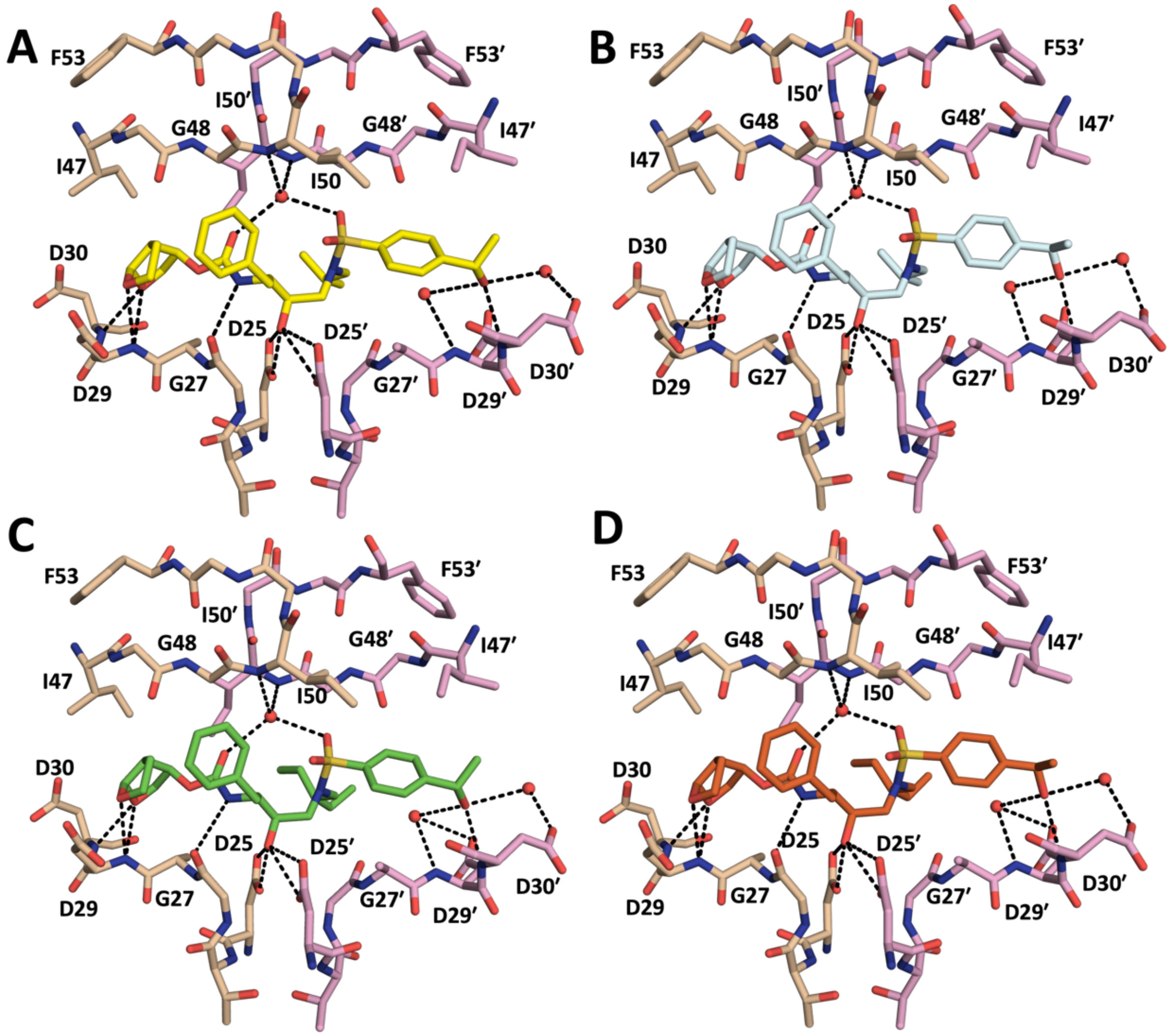

Figure S3. Crystal structures of wild-type HIV-1 protease in complex with inhibitors (A) **1**, (B) **2**, (C) **3**, and (D) **4**. Hydrogne bonding interactions are shown in black dashed lines. The two protease monomers are shown in gold (denoted as nonprime) and pink (denoted as prime) colors.

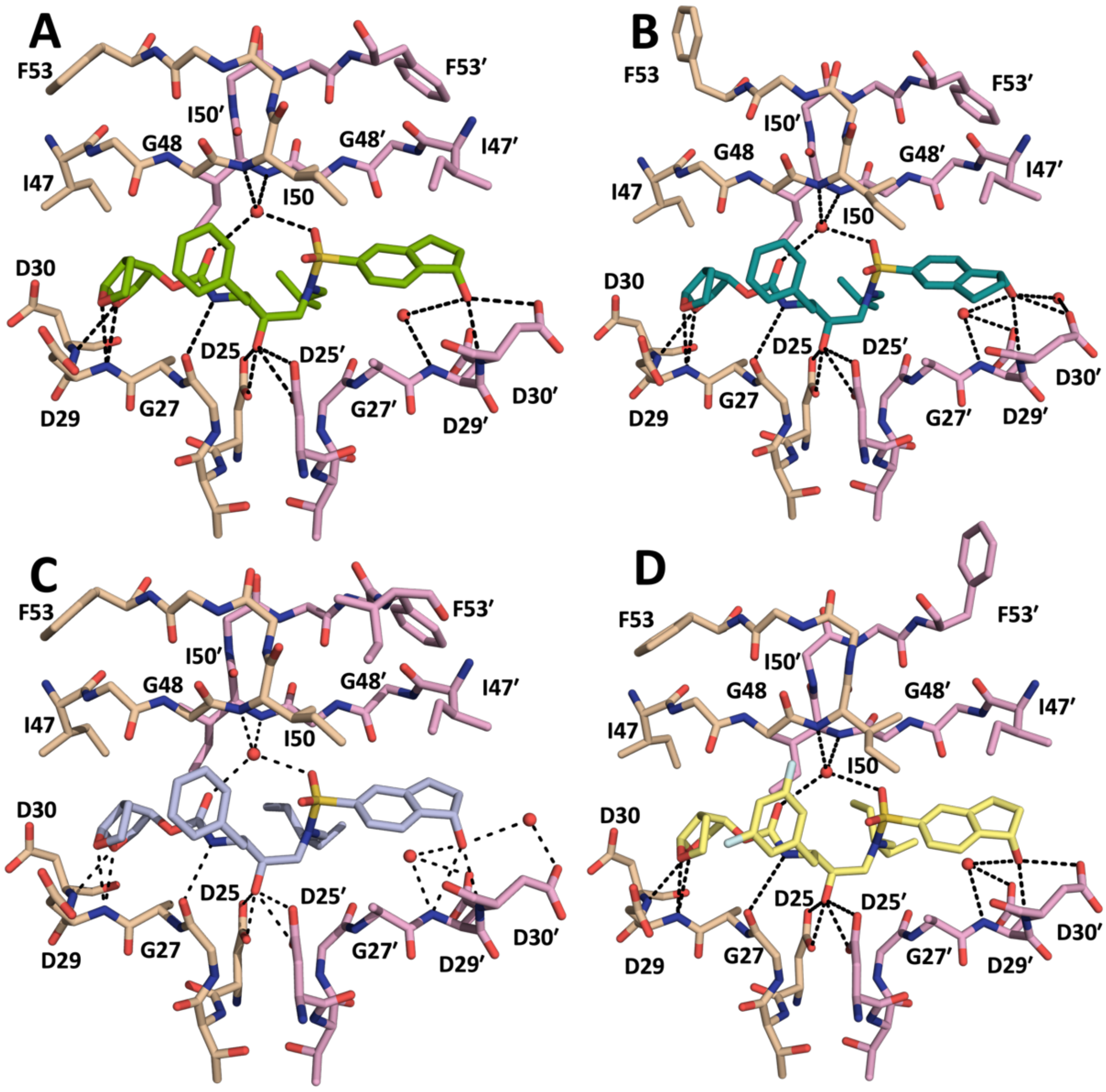

Figure S4. Crystal structures of wild-type HIV-1 protease in complex with inhibitors (A) **11**, (B) **12**, (C) **14**, and (D) **18**. Hydrogen bonding interactions are shown in black dashed lines. The two protease monomers are shown in gold (denoted as nonprime) and pink (denoted as prime) colors.

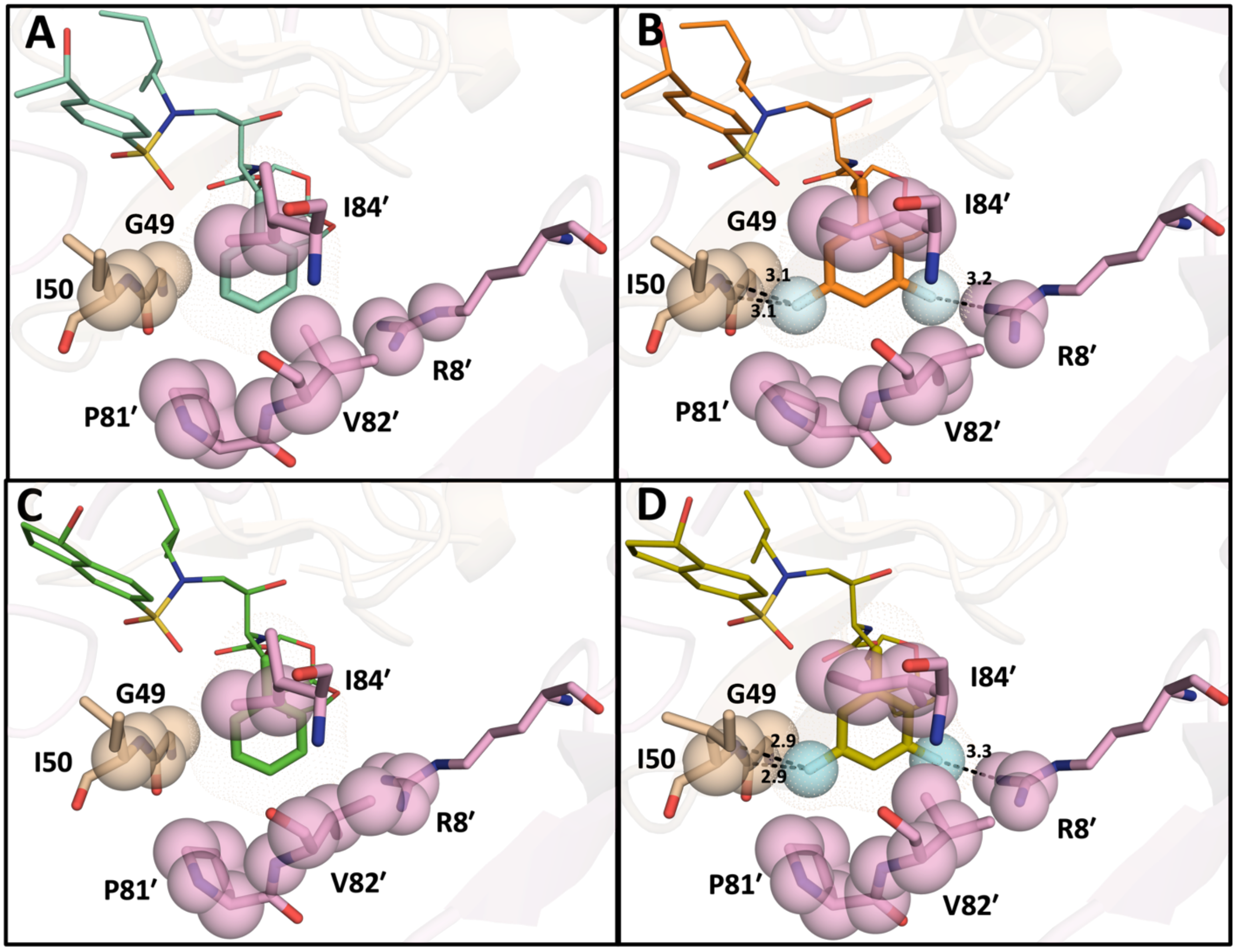

Figure S5. Comparison of the binding interactions with the protease for the P1 phenyl moiety of compound **3** (A) and **11** (C) and the P1 difluorophenyl moiety of compound **9** (B) and **15** (D) in the S1 subsite of HIV-1 Protease. The van der Waals spheres of residues are shown in pink (Chain B) and gold (Chain A) colors, and fluorine atoms in cyan color. The fluorine mediated multipolar interactions are shown as black dashed lines.

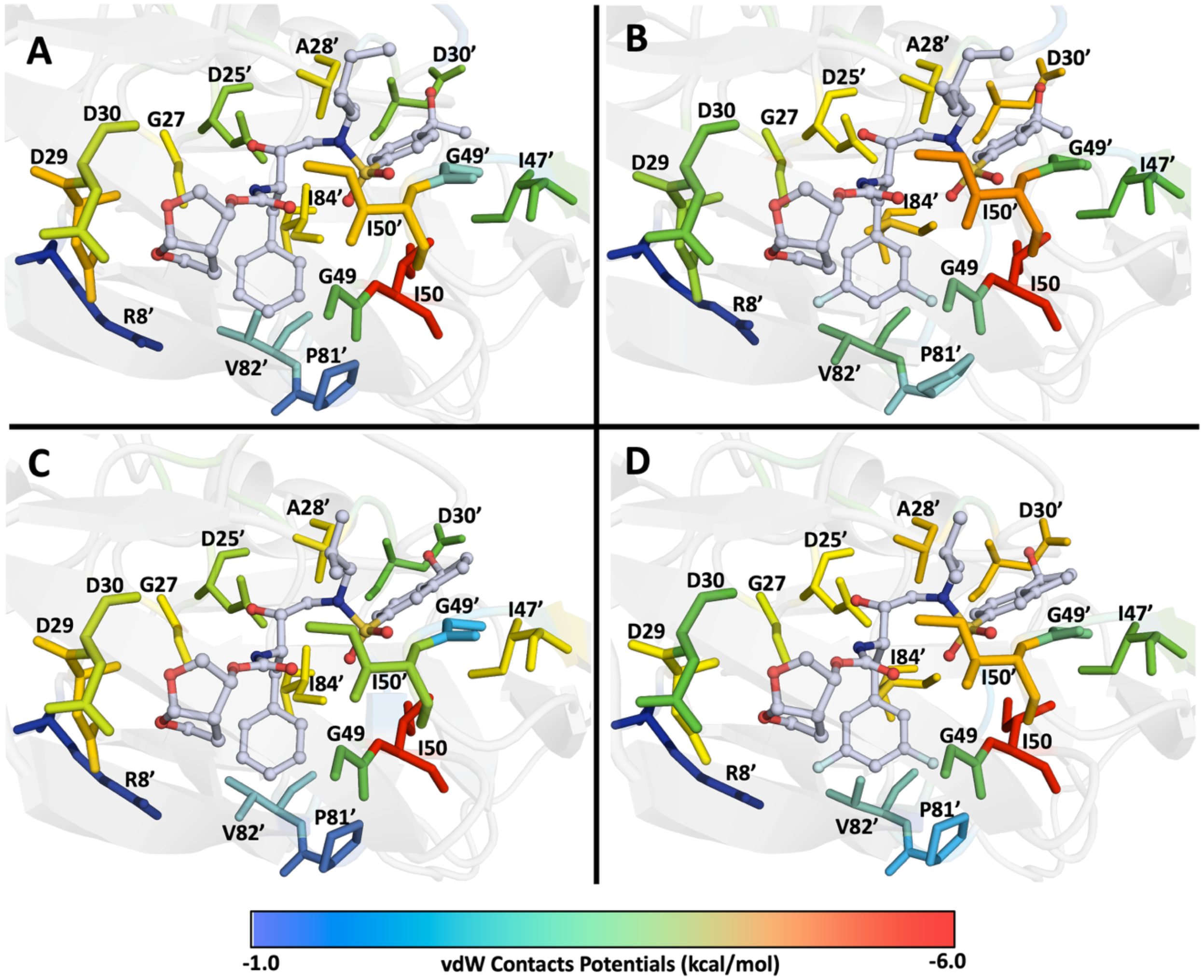

Figure S6. Comparison of crystal structures showing packing of the P1 phenyl ring of compounds **3** (A) and **11** (C) in comparison to the P1 difluorophenyl moiety of compounds **9** (B) and **15** (D) within the S1 binding pocket of HIV-1 protease. The active site residues are colored from blue to red rainbow spectrum, indicating increase of van der Waals contact potentials with the inhibitors mapped onto the inhibitor cocrystal structures.

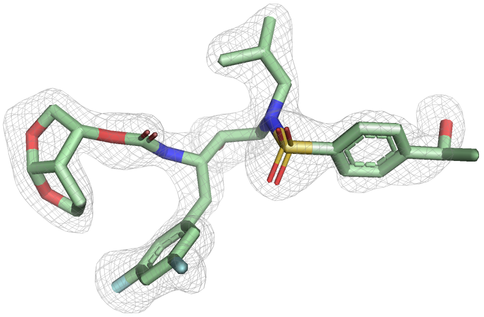

Compound **5**

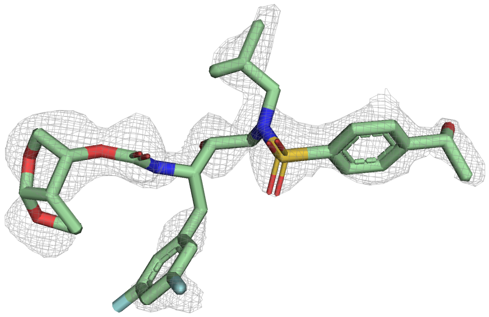

Compound **6**

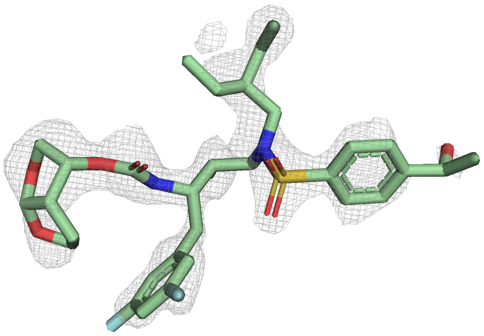

Compound **9**

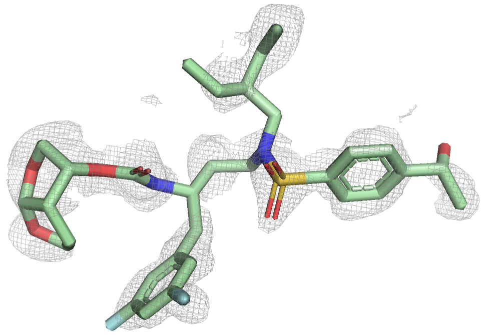

Compound **10**

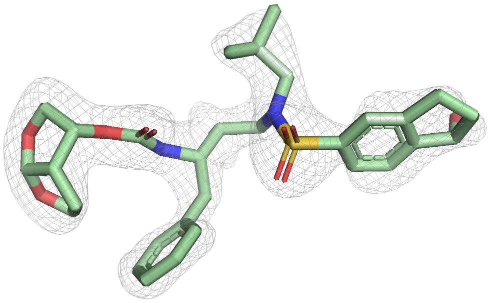

Compound **11**

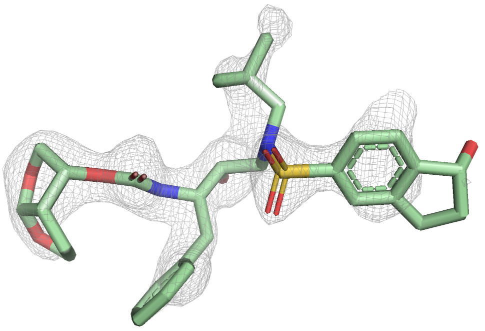

Compound **12**

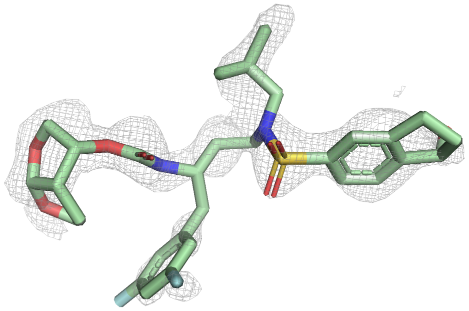

Compound **15**

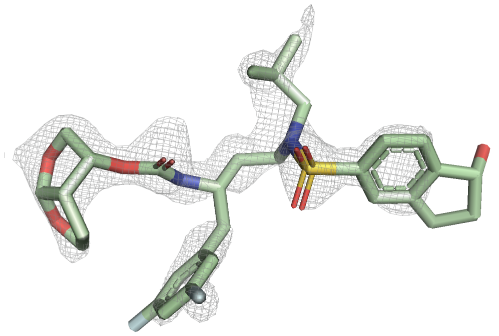

Compound **16**

Figure S7. F_o–F_c electron density maps (grey mesh) contoured at 3σ for representative inhibitors.

Table S2. X-ray data collection and crystallographic refinement statistics of Inhibitors 6–10.

**Inhibitor**	**6**	**7**	**8**	**9**	**10**
**PDB ID**	PDB_00009Q1C	PDB_00009Q3P	PDB_00009Q5D	PDB_00009PZ1	PDB_00009PYX
**Data Collection**					
Location	NSLS-II	NSLS-II	NSLS-II	NSLS-II	NSLS-II
Resolution Range (Å)	19.23–1.94 (2.01–1.94)	19.22–1.90 (1.97–1.90)	19.86–1.94 (2.01–1.94)	19.25–1.80 (1.87–1.80)	53.99–1.73 (1.79–1.73)
Space Group	P 6_1	P 6_1	P 6_1	P 6_1	P 6_1
a,b,c, (Å)	62.3, 62.3, 82.2	62.4, 62.4, 82.0	62.5, 62.5, 82.2	62.5, 62.5, 82.1	62.3, 62.3, 82.0
α, β, γ (°)	90, 90, 120	90, 90, 120	90, 90, 120	90, 90, 120	90, 90, 120
Total Reflections	26477 (2592)	28550 (2828)	27078 (2724)	33742 (3359)	37051 (3757)
Unique Reflections	13265 (1299)	14286 (1414)	13540 (1362)	16876 (1680)	18754 (1879)
Multiplicity	2.0 (2.0)	2.0 (2.0)	2.0 (2.0)	2.0 (2.0)	2.0 (2.0)
Completeness (%)	98.8 (98.0)	99.7 (99.2)	100.0 (100.0)	99.8 (99.5)	99.1 (99.8)
(Average I)/sigma	16.5 (3.5)	18.4 (4.9)	20.4 (4.9)	15.2 (2.4)	19.5 (3.3)
Wilson B-Factor	38.66	35.21	29.76	38.91	23.51
R_{merge}^{a}	0.020 (0.188)	0.019 (0.126)	0.023 (0.145)	0.021 (0.270)	0.021 (0.204)
CC1/2	0.999 (0.911)	0.999 (0.958)	1.000 (0.938)	0.999 (0.839)	0.999 (0.875)
**Twin Fraction**	0.510	0.500	0.490	0.500	0.540
**Twin Operator**	h,–h–k,–l	h,–h–k,–l	h,–h–k,–l	h,–h–k,–l	h,–h–k,–l
**Refinement**					
R_{factor}^{b}	0.2013 (0.2931)	0.2132 (0.3162)	0.2105 (0.2467)	0.2104 (0.3235)	0.1932 (0.2833)
R_{free}^{c}	0.2521 (0.3169)	0.2422 (0.2378)	0.2435 (0.2915)	0.2531 (0.3146)	0.2466 (0.3163)
**RMSD[d] in:**					
Bond Lengths (Å)	0.002	0.002	0.002	0.005	0.005
Bond Angles (°)	0.590	0.560	0.630	0.850	0.900
**Ramachandran:**					
Favored (%)	98.97	98.96	98.45	97.94	99.48
Allowed (%)	1.03	0.63	1.55	2.06	0.52
Outliers (%)	0.00	0.00	0.00	0.00	0.00
Rotamer outliers (%)	0.62	0.63	0.62	1.91	0.62
**B-Factors:**					
Average	38.66	35.21	27.38	38.91	23.51
Macromolecules	38.96	35.17	26.84	38.90	22.60
Ligand	34.89	33.41	27.20	37.80	23.82
Solvent	35.96	36.08	32.25	39.48	30.46

$^{a}R_{merge} = \Sigma \mid I - \langle I \rangle \mid / \Sigma I$, where I = observed intensity, <I> = average intensity over symmetry equivalent.

$^{b}R_{factor} = \Sigma \| F_o| - |F_c\| / \Sigma|F_o|$.

$^{c}R_{free}$ was calculated from 5% of reflections, chosen randomly, which were omitted from the refinement process

[d]RMSD, root mean square deviation.

Table S3. X-ray data collection and crystallographic refinement statistics of Inhibitors 12, 15, 16 and 18.

Inhibitor	12	15	16	18
**PDB ID**	PDB_00009Q3T	PDB_00009Q0T	PDB_00009Q13	PDB_00009Q1P
**Data Collection**				
Location	NSLS-II	NSLS-II	NSLS-II	NSLS-II
Resolution Range (Å)	19.80–1.87 (1.93–1.87)	53.92–1.87 (1.93–1.87)	19.81–1.71 (1.78–1.72)	19.26–1.87 (1.93–1.87)
Space Group	$P\ 6_1$	$P\ 6_1$	$P\ 6_1$	$P\ 6_1$
a,b,c, (Å)	62.3, 62.3, 82.1	62.2, 62.2, 81.7	62.3, 62.3, 82.3	62.5, 62.5, 82.1
α, β, γ (°)	90, 90, 120	90, 90, 120	90, 90, 120	90, 90, 120
Total Reflections	30127 (2999)	29478 (2960)	38220 (3793)	30191 (3008)
Unique Reflections	15128 (1510)	14828 (1492)	19138 (1901)	15102 (1503)
Multiplicity	2.0 (2.0)	2.0 (2.0)	2.0 (2.0)	2.0 (2.0)
Completeness (%)	99.9 (100.0)	99.5 (99.0)	98.4 (97.4)	99.9 (100.0)
(Average I)/sigma	13.0 (2.6)	13.3 (2.7)	14.3 (2.4)	13.8 (2.3)
Wilson B-Factor	41.15	35.79	34.47	41.26
$R_{merge}{}^{a}$	0.026 (0.253)	0.026 (0.253)	0.022 (0.261)	0.024 (0.282)
CC1/2	0.999 (0.875)	0.999 (0.859)	0.999 (0.821)	0.999 (0.836)
**Twin Fraction**	0.500	0.480	0.490	0.490
**Twin Operator**	h,–h–k,–l	h,–h–k,–l	h,–h–k,–l	h,–h–k,–l
**REFINEMENT**				
$R_{factor}{}^{b}$	0.2001 (0.3005)	0.2030 (0.2936)	0.2125 (0.3311)	0.2258 (0.3190)
$R_{free}{}^{c}$	0.2462 (0.3049)	0.2335 (0.3128)	0.2374 (0.3297)	0.2548 (0.3530)
**RMSD[d] in:**				
Bond Lengths (Å)	0.009	0.003	0.002	0.008
Bond Angles (°)	0.900	0.600	0.580	0.840
**Ramachandran:**				
Favored (%)	99.48	100	98.97	97.42
Allowed (%)	0.52	0.00	1.03	2.06
Outliers (%)	0.00	0.00	0.00	0.52
Rotamer outliers (%)	1.85	2.44	1.24	1.24
**B-Factors:**				
Average	41.15	35.79	34.47	41.28
Macromolecules	41.14	35.54	34.05	41.72
Ligand	41.67	41.05	41.13	35.41
Solvent	41.12	36.18	36.36	37.49

[a] $R_{merge} = \Sigma \mid I - \langle I \rangle \mid / \Sigma I$, where I = observed intensity, <I> = average intensity over symmetry equivalent.

[b] $R_{factor} = \Sigma \mid\mid F_o \mid - \mid F_c \mid\mid / \Sigma \mid F_o \mid$.

[c] R_{free} was calculated from 5% of reflections, chosen randomly, which were omitted from the refinement process

[d] RMSD, root mean square deviation.

Table S4. X-ray data collection and crystallographic refinement statistics of Inhibitors 5, 11, 13 and 14.

Inhibitor	5	11	13	14
**PDB ID**	PDB_00009YRR	PDB_00009YKP	PDB_00009YRY	PDB_00009YRA
**Data Collection**				
Location	APS	APS	APS	APS
Resolution Range (Å)	23.53–1.89 (1.96–1.89)	23.60–1.96 (2.03–1.96)	23.59–1.95 (2.01–1.95)	24.16–1.91 (1.98–1.91)
Space Group	$P\ 2_1\ 2_1\ 2_1$	$P\ 2_1\ 2_1\ 2_1$	$P\ 2_1\ 2_1\ 2_1$	$P\ 2_1\ 2_1\ 2_1$
a,b,c, (Å)	50.9, 58.8, 61.6	51.0, 58.5, 62.1	51.0, 58.3, 62.2	51.1, 58.4, 62.0
α, β, γ (°)	90, 90, 90	90, 90, 90	90, 90, 90	90, 90, 90
Unique Reflections	14544 (1281)	13588 (1274)	13723 (1245)	14857 (1414)
Multiplicity	7.3 (6.6)	6.9 (5.5)	6.8 (4.5)	6.7 (4.4)
Completeness (%)	94.8 (85.4)	98.2 (93.8)	97.7 (90.5)	99.5 (96.6)
(Average I)/sigma	30.6 (4.2)	24.6 (3.2)	28.7 (3.7)	26.5 (3.5)
Wilson B-Factor	21.81	23.74	21.76	23.90
$R_{merge}{}^{a}$	0.059 (0.382)	0.075 (0.489)	0.063 (0.341)	0.070 (0.420)
**REFINEMENT**				
$R_{factor}{}^{b}$	0.1812 (0.2141)	0.1809 (0.2220)	0.1763 (0.1968)	0.1864 (0.2490)
$R_{free}{}^{c}$	0.2272 (0.3173)	0.2267 (0.2374)	0.2148 (0.3010)	0.2337 (0.3429)
**RMSD[d] in:**				
Bond Lengths (Å)	0.003	0.003	0.004	0.004
Bond Angles (°)	0.590	0.623	0.760	0.730
**Ramachandran:**				
Favored (%)	100	99.48	99.48	100
Allowed (%)	0.00	0.52	0.52	0.00
Outliers (%)	0.00	0.00	0.00	0.00
Rotamer outliers (%)	0.00	0.00	0.00	0.00
**B-Factors:**				
Average	27.08	28.94	25.04	28.07
Macromolecules	25.80	27.66	24.05	27.25
Ligand	30.29	39.56	28.72	27.85
Solvent	36.20	36.10	32.29	35.03

[a]$R_{merge} = \Sigma \mid I - \langle I \rangle \mid / \Sigma I$, where I = observed intensity, <I> = average intensity over symmetry equivalent.

[b]$R_{factor} = \Sigma \| F_o| - |F_c\| / \Sigma |F_o|$.

[c]R_{free} was calculated from 5% of reflections, chosen randomly, which were omitted from the refinement process

[d]RMSD, root mean square deviation.

Table S5. Intermolecular hydrogen bonds between the protease and inhibitors in the cocrystal structures (distance in Å).

Residue	D30	D29	D29	D27	D25	D25	D25	D25	I50	I50	I50'	I50'	D30'	D30'	D30'	D30'	D30'	D29'	D29'	D29'	D29'	D29'	D29'
Atom	MC-N	MC-N	MC-N	MC-O	SC-O	SC-O	SC-O	SC-O	MC-N	MC-N	MC-N	MC-N	SC-O	SC-O	SC-O	SC-O	MC-N	MC-N	MC-O	H2O	MC-N	MC-O	SC-O
Type	Direct	Direct	Direct	Direct	Direct	Direct	Direct	Direct	Water	Water	Water	Water	Water	Water	Direct	Direct	Direct	Direct	Direct	Water	Water	Water	water
DRV	3.1	3.2	3.0	3.5	3.1	2.9	3.0	3.0	2.9	3.1	2.9	3.1	3.4	2.8			3.5						
1	3.1	3.1	3.0	3.3	3.3	2.8	3.0	2.9	2.9	3.0	2.8	3.1	3.1	2.8			3.2			3.6	3.5		3.4
2	3.0	3.1	2.9	3.4	3.4	2.8	3.0	2.9	2.8	3.0	2.8	3.1	3.1	2.8			3.2			3.2	3.3		3.3
3	3.1	3.1	3.0	3.6	3.4	2.7	3.0	2.9	2.8	3.1	2.9	3.1	3.2	2.8			3.1			3.2	3.2		3.4
4	3.1	3.2	3.0	3.3	3.3	2.8	3.0	2.9	2.8	3.0	2.8	3.1	3.0	2.8			3.2			3.1	3.2		3.3
5	3.1	3.1	3.0	3.2	3.3	2.7	2.8	3.0	2.8	3.1	2.8	3.1	3.0	2.7			3.2			3.1	3.1		3.2
6	3.2	3.2	3.1	3.4	3.0	2.7	2.8	3.0	2.8	3.0	2.7	3.2			2.9		3.1			3.7			2.8
7	3.4	3.4	3.3	3.5	3.0	2.7	2.9	2.9	2.9	3.0	2.8	3.1	3.6	3.2	2.8		3.2			3.2	3.2		3.0
8	3.5	3.3	3.1	3.5	3.0	2.7	2.8	3.0	2.8	3.0	2.7	3.1	3.2	3.2	2.8		3.2			3.4	3.3		3.0
9	3.2	3.2	3.2	3.8	2.9	2.8	2.9	3.1	2.9	3.1	2.8	3.1	3.7	3.2	3.0		3.4			3.1	3.4		3.0
10	3.6	3.6	3.4	3.4	3.0	2.8	2.8	3.0	2.8	2.9	2.7	3.1	3.0	2.8			3.1			3.1	3.3		3.1
11	3.1	3.1	3.0	3.4	3.3	2.8	3.0	2.9	2.8	3.0	2.8	3.1			3.1		2.9			4.0	3.2		
12	3.3	3.3	3.2	3.6	3.0	2.7	3.0	2.9	2.8	3.2	2.7	3.0	3.4	3.6	3.1		3.6			3.3	3.2		3.1
13	3.0	3.1	3.1	3.3	3.4	2.8	3.0	2.9	2.8	3.0	2.8	3.1	3.2	2.8			3.0			3.6	3.2		3.4
14	3.1	3.2	3.0	3.4	3.3	2.8	3.0	2.9	2.8	3.0	2.8	3.1	3.2	2.8			3.0	3.5		3.2	3.1		3.3
15	3.3	3.4	3.1	3.6	3.0	2.7	3.0	2.8	2.8	3.0	2.7	3.1			2.9		2.9			3.6	3.6		3.0
16	3.5	3.4	3.2	3.5	3.0	2.8	3.0	2.9	2.9	3.0	2.8	3.3	3.1	2.8	3.4		3.4			3.2	3.2		3.0
18	3.4	3.4	3.0	3.8	3.0	2.8	3.0	2.8	2.9	3.0	2.8	3.1			2.9		2.9			3.3	3.2		2.9

MC: main chain; SC: side chain

Synthesis and Characterization Data of Intermediates and Target Compounds

***tert*-Butyl ((2*S*,3*R*)-3-hydroxy-4-(isobutylamino)-1-phenylbutan-2-yl)carbamate (21a).**

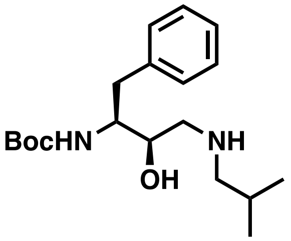

A solution of chiral epoxide (1*S*,2*S*)-(1-oxiranyl-2-phenylethyl)carbamic acid *tert*-butyl ester (13.0 g, 49.4 mmol) in EtOH (250 mL) was treated with isobutylamine (40 mL) at room temperature and the resulting reaction mixture was heated at 80 °C for 3 h. Upon completion of the reaction, the reaction mixture was cooled to room temperature. The solvents were removed under reduced pressure, and the residue was dried under high vacuum. Recrystallization from a mixture of EtOAc/hexanes (1:5) (100 mL) provided the amino alcohol **21a** (16.0 g, 96%) as a white solid. ^{1}H NMR (500 MHz, $CDCl_3$) δ 7.30–7.27 (m, 2H), 7.23–7.18 (m, 3H), 4.71 (d, J = 8.5 Hz, 1H), 3.81 (m, 1H), 3.46 (dd, J = 11.5, 5.0 Hz, 1H), 2.99 (dd, J = 14.0, 4.5 Hz, 1H), 2.86 (dd, J = 13.5, 7.5 Hz, 1H), 3.69 (d, J = 3.5 Hz, 2H), 2.45–2.38 (m, 2H), 1.77–1.68 (m, 1H), 1.35 (s, 9H), 0.92 (d, J = 7.0 Hz, 3H, overlapping), 0.91 (d, J = 7.0 Hz, 3H, overlapping); ^{13}C NMR (126 MHz, $CDCl_3$) δ 156.09, 138.04, 129.68, 128.53, 126.45, 79.52, 70.78, 58.06, 54.27, 51.60, 36.83, 28.47, 28.43, 20.68, 20.66; MS (APCI) *m/z*: calcd for $C_{19}H_{33}N_2O_3$ $[M + H]^+$: 337.48; found 337.21.

***tert*-Butyl ((2*S*,3*R*)-3-hydroxy-4-(((*S*)-2-methylbutyl)amino)-1-phenylbutan-2-yl)carbamate (21b).**

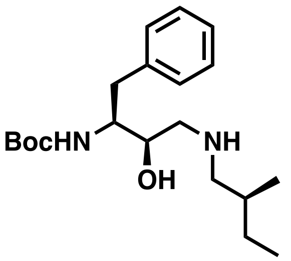

The same procedure was used as described above for compound **21a**. Chiral epoxide (1*S*,2*S*)-(1-oxiranyl-2-phenylethyl)carbamic acid *tert*-butyl ester (3.50 g, 13.3 mmol) was reacted with (*S*)-2-methylbutan-1-amine (1.31 g, 15.0 mmol), and the crude product was purified by recrystallization

from a mixture of EtOAc/hexanes (1:9) (40 mL) to provide the amino alcohol **21b** (3.83 g, 82%) as a white solid. ^{1}H NMR (500 MHz, $CDCl_3$) δ 7.30–7.27 (m, 2H), 7.24–7.19 (m, 3H), 4.74 (d, J = 8.7 Hz, 1H), 3.82 (m, 1H), 3.47 (dd, J = 11.5, 5.0 Hz, 1H), 2.99 (dd, J = 14.0, 4.5 Hz, 1H), 2.89–2.84 (m, 1H), 2.70 (d, J = 4.5 Hz, 2H), 2.52 (dd, J = 11.5, 6.0 Hz, 1H), 2.42 (dd, J = 11.5, 7.0 Hz, 1H), 1.56–1.47 (m, 1H), 1.46–1.37 (m, 1H), 1.35 (s, 9H), 1.18–1.11 (m, 1H), 0.92–0.86 (m, 6H); ^{13}C NMR (126 MHz, $CDCl_3$) δ 156.14, 138.00, 129.69, 128.53, 126.46, 79.57, 70.71, 56.06, 54.24, 51.66, 36.83, 34.85, 28.42, 27.42, 17.65, 11.40; MS (APCI) *m/z*: calcd for $C_{20}H_{35}N_2O_3$ $[M + H]^+$: 351.51; found 351.22.

***tert*-Butyl ((2*S*,3*R*)-4-((2-Ethylbutyl)amino)-3-hydroxy-1-phenylbutan-2-yl)carbamate (21c).**

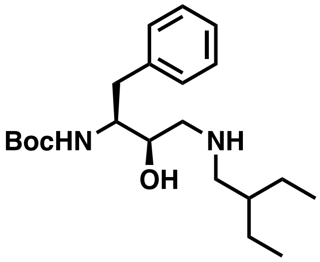

The same procedure was used as described above for compound **21a**. Chiral epoxide (1*S*,2*S*)-(1-oxiranyl-2-phenylethyl)carbamic acid *tert*-butyl ester (4.70 g, 17.9 mmol) was reacted with 2-ethyl-*n*-butylamine (1.98 g, 19.6 mmol), and the crude product was purified by recrystallization from a mixture of EtOAc/hexanes (1:9) (50 mL) to provide the amino alcohol **21c** (5.20 g, 80%) as a white solid. ^{1}H NMR (500 MHz, $CDCl_3$) δ 7.30–7.27 (m, 2H), 7.24–7.19 (m, 3H), 4.74 (d, J = 8.5 Hz, 1H), 3.82 (m, 1H), 3.47 (dd, J = 11.5, 5.0 Hz, 1H), 2.99 (dd, J = 14.0, 5.0 Hz, 1H), 2.92–2.85 (m, 1H), 2.71 (d, J = 3.5 Hz, 2H), 2.56–2.48 (m, 2H), 1.36 (s, 9H, overlapping), 1.35–1.20 (m, 5H), 0.87 (t, J = 7.0 Hz, 6H); ^{13}C NMR (126 MHz, $CDCl_3$) δ 156.17, 137.99, 129.69, 128.54, 126.47, 79.58, 70.68, 54.24, 52.68, 51.83, 40.95, 36.87, 28.43, 24.00, 11.06, 11.02; MS (APCI) *m/z*: calcd for $C_{21}H_{37}N_2O_3$ $[M + H]^+$: 365.54; found 365.20.

***tert*-Butyl-((2*S*,3*R*)-1-(3,5-difluorophenyl)-3-hydroxy-4-(((*S*)-2-methylbutyl)amino)butan-2-yl)carbamate (22b).**

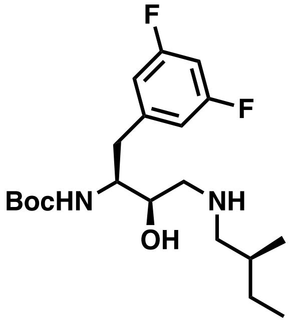

The same procedure was used as described above for compound **21a**. A solution of chiral epoxide *tert*-butyl ((*S*)-2-(3,5-difluorophenyl)-1-((*S*)-oxiran-2-yl)ethyl)carbamate **20** (0.70 g, 2.34 mmol) in EtOH (15 mL) was treated with (*S*)-2-methylbutan-1-amine (0.33 mL, 2.80 mmol) to provide the amino alcohol **22b** (0.82 g, 92%) as a white solid. ^{1}H NMR (500 MHz, $CDCl_3$) δ 6.78–6.74 (m, 2H), 6.67–6.62 (m, 1H), 4.73 (d, J = 9.5 Hz, 1H), 3.80–3.74 (m, 1H), 3.48 (ddd, J = 10.6, 6.9, 3.7 Hz, 1H), 3.01 (dd, J = 14.1, 4.2 Hz, 2H), 2.80–2.67 (m, 3H), 2.53 (dd, J = 5.8, 11.7 Hz, 1H), 2.43 (dd, J = 11.7, 7.3 Hz, 1H), 1.56–1.48 (m, 1H), 1.45–1.38 (m, 1H), 1.36 (s, 9H), 1.19–1.11 (m, 1H), 0.92–0.87 (m, 6H) ppm; ^{13}C NMR (126 MHz, $CDCl_3$) δ 163.90 (d, J = 12.8 Hz), 161.93 (d, J = 12.8 Hz), 155.79 , 142.30 (t, J = 9.1 Hz), 112.49 (d, J = 5.6 Hz), 112.35 (d, J = 5.6 Hz), 101.82 (t, J = 25.2 Hz), 79.74, 70.54, 55.83, 54.03, 51.58, 36.66, 34.60, 28.23, 27.26, 17.49, 11.23 ppm; ^{19}F NMR (470 MHz, $CDCl_3$) δ −110.48 ppm; MS (APCI) *m/z*: calcd for $C_{20}H_{33}F_2N_2O_3$ $[M + H]^+$: 387.49; found 387.20.

***tert*-Butyl((2*S*,3*R*)-1-(3,5-difluorophenyl)-4-((2-ethylbutyl)amino)-3-hydroxybutan-2-yl)carbamate (22c).**

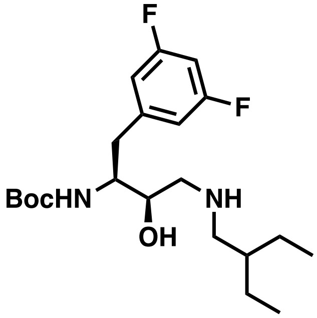

The same procedure was used as described above for compound **21a**. Chiral epoxide *tert*-butyl ((*S*)-2-(3,5-difluorophenyl)-1-((*S*)-oxiran-2-yl)ethyl)carbamate **20** (2.0 g, 6.68 mmol) was reacted with 2-ethyl-*n*-butylamine (0.81 g, 8.00 mmol), and the crude product was purified by

recrystallization from a mixture of EtOAc/hexanes (1:9) (20 mL) to provide the amino alcohol **22c** (2.54 g, 95%) as a white solid. ^{1}H NMR (500 MHz, $CDCl_3$) δ 6.77–6.74 (m, 2H), 6.67–6.63 (m, 1H), 4.72 (d, J = 9.0 Hz, 1H), 3.79–3.74 (m, 1H), 3.45 (q, J = 5.6 Hz, 1H), 3.00 (dd, J = 14.2, 4.2 Hz, 1H), 2.81–2.65 (m, 3H), 2.54–2.49 (m, 2H), 1.34–1.36 (m, overlapping, 14H), 0.87 (t, J = 6.9, 6H) ppm; ^{13}C NMR (126 MHz, $CDCl_3$) δ 163.91 (d, J = 12.9 Hz), 161.94 (d, J = 12.9 Hz, 2H), 155.85, 142.25 (t, J = 9.0 Hz), 112.51 (d, J = 5.6 Hz), 112.36 (d, J = 5.6 Hz), 101.85 (t, J = 25.1 Hz), 79.79, 70.51, 53.99, 52.47, 51.74, 40.66, 36.68, 28.23, 23.81, 10.87, 10.85 ppm; ^{19}F NMR (470 MHz, $CDCl_3$) δ −110.40 ppm; MS (APCI) *m/z*: calcd for $C_{21}H_{35}F_2N_2O_3$ $[M + H]^+$: 401.52; found 401.24.

***tert*-Butyl((2*S*,3*R*)-4-((4-acetyl-*N*-((*S*)-2-methylbutyl)phenyl)sulfonamido)-1-(3,5-difluorophenyl)-3-hydroxybutan-2-yl)carbamate (23b).**

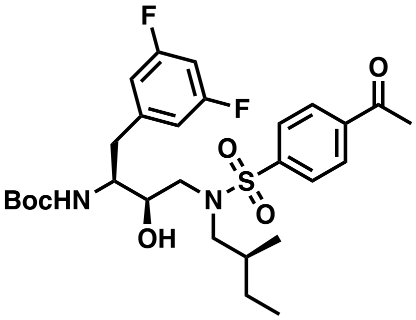

A solution of compound **22b** (0.70 g, 1.90 mmol) in EtOAc (12 mL) at room temperature was treated with a solution of Na_2CO_3 (0.40 g, 3.80 mmol) in H_2O (12 mL) followed by the addition of 4-acetylbenzenesulfonyl chloride (0.54 g, 2.47 mmol). The resulting biphasic reaction mixture was stirred at room temperature overnight and then diluted with EtOAc (100 mL). The layers were separated, and the aqueous layer was further extracted with EtOAc (100 mL). The combined organic fractions were washed with saturated aqueous NaCl solution (150 mL), dried over anhydrous Na_2SO_4, filtered, and then concentrated under reduced pressure. The residue obtained was purified by automated flash column chromatography using a silica gel column (SiliaSep 40 g, gradient elution with 0–50% EtOAc/hexanes) to give compound **23b** (0.95 g, 88%) as a white solid. ^{1}H NMR (500 MHz, $CDCl_3$) δ 8.07 (d, J = 8.3 Hz, 2H), 7.88 (d, J = 8.3 Hz, 2H), 6.80–6.76 (m, 2H), 6.68–6.64 (tt, J = 9.0, 2.0 Hz, 1H), 4.63 (d, J = 8.3 Hz, 1H), 3.91 (s, 1H), 3.81 (s, 1H), 3.73–3.68 (m, 1H), 3.16–2.98 (m, 4H), 2.92–2.86 (m, 2H), 2.65 (s, 3H), 1.65–1.58 (m, 1H), 1.49–1.41 (m, 1H), 1.36 (s, 9H), 1.11–1.03 (m, 1H), 0.87–0.83 (m, overlapping, 6H) ppm; ^{13}C NMR

(126 MHz, $CDCl_3$) δ 196.64, 163.97 (d, J = 13.0 Hz), 162.00 (d, J = 12.7 Hz), 155.9, 142.20, 141.96 (t, J = 9.1 Hz), 140.14, 129.01, 127.64, 112.56 (d, J = 5.6 Hz), 112.41 (d, J = 5.5 Hz), 102.04 (t, J = 25.3 Hz), 80.20, 72.60, 57.20, 54.52, 53.45, 35.04, 33.37, 28.20, 26.85, 26.48, 16.84, 11.01 ppm; ^{19}F NMR (470 MHz, $CDCl_3$) δ −110.14 ppm; MS (APCI) *m/z*: calcd for $C_{28}H_{39}F_2N_2O_6S$ $[M + H]^+$: 569.68; found 569.15.

***tert*-Butyl ((2*S*,3*R*)-4-((4-acetyl-*N*-(2-ethylbutyl)phenyl)sulfonamido)-1-(3,5-difluorophenyl)-3-hydroxybutan-2-yl)carbamate (23c)**.

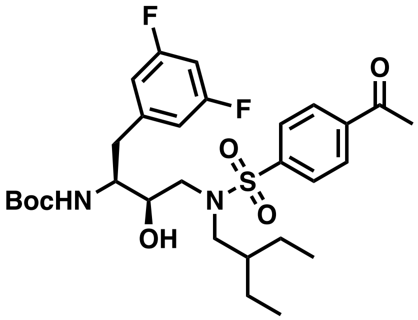

The same procedure was used as described above for compound **23b.** A solution of compound **22c** (0.80 g, 2.00 mmol) in EtOAc (15 mL) was treated with a solution of Na_2CO_3 (0.57 g, 2.60 mmol) in H_2O (15 mL) and 4-acetylbenzenesulfonyl chloride (0.42 g, 4.00 mmol) to give compound **23c** (0.77 g, 66%) as a white solid. ^1H NMR (500 MHz, $CDCl_3$) δ 8.08 (d, J = 8.5 Hz, 2H), 7.88 (d, J = 8.5 Hz, 2H), 6.80–6.76 (m, 2H), 6.68–6.64 (m, 1H), 4.59 (d, J = 8.4 Hz, 1H), 3.94 (s, 1H), 3.78–3.77 (m, 1H), 3.73–3.67 (m, 1H), 3.10– 2.99 (m, 4H), 2.95–2.86 (m, 2H), 2.65 (s, 3H), 1.49–1.35 (m, 2H), 1.35 (s, 9H), 1.31–1.26 (m, 3H), 0.84–0.81 (m, 6H) ppm; ^{13}C NMR (126 MHz, $CDCl_3$) δ 196.65, 163.97 (d, J = 12.9 Hz), 162.00 (d, 12.7 Hz), 155.94, 142.06 (t, J = 9.1 Hz), 141.93, 140.16, 129.03, 127.67, 112.56 (d, J = 5.8 Hz), 112.41 (d, J = 5.7 Hz), 102.04 (t, J = 25.2 Hz), 80.19, 72.84, 54.79, 54.44, 53.53, 38.97, 35.25, 28.20, 26.86, 22.97, 22.74, 10.50, 10.27 ppm; ^{19}F NMR (470 MHz, $CDCl_3$) δ −110.14 ppm; MS (APCI) *m/z*: calcd for $C_{29}H_{41}F_2N_2O_6S$ $[M + H]^+$: 583.71; found 583.22.

***tert*-Butyl ((2*S*,3*R*)-1-(3,5-difluorophenyl)-3-hydroxy-4-((4-((*S*)-1-hydroxyethyl)-*N*-((*S*)-2-methylbutyl)phenyl)sulfonamido)butan-2-yl)carbamate (24b).**

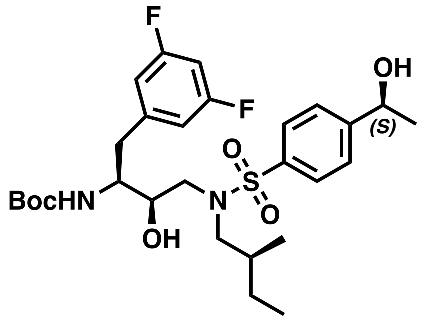

A solution of **23b** (0.18 g, 0.32 mmol) in anhydrous THF (17 mL) under argon was cooled to 0 °C, and then the chiral catalyst (*R*)-2-methyl-CBS-oxazaborolidine (0.18 g, 0.64 mmol) was added. After stirring the reaction mixture at 0 °C for 15 min, a solution of BH_3-THF complex (1 M in THF) (0.54 mL, 0.48 mmol) was added dropwise over 1 h. Stirring was continued at 0 °C until the reaction was complete, as monitored by TLC. The reaction was quenched with a mixture of acetone/methanol (1:1, 10 mL) and the solvents were evaporated under reduced pressure. The crude product was purified by automated flash chromatography using a silica gel column (SiliaSep 25 g, gradient elution with 0–60% EtOAc/hexanes) to give compound **24b** as a white solid (0.16 g, 87%). ^{1}H NMR (500 MHz, $CDCl_3$) δ 7.74 (d, *J* = 8.2 Hz, 2H), 7.52 (d, *J* = 8.2 Hz, 2H), 6.79–6.78 (m, 2H), 6.67–6.63 (m, 1H), 4.98 (q, *J* = 6.4 Hz, 1H), 4.66 (d, *J* = 8.4 Hz, 1H), 3.97 (s, 1H), 3.79 (s, 1H), 3.73–3.69 (m, 1H), 3.06–2.99 (m, 4H), 2.91–2.81 (m, 2H), 1.62–1.59 (m, 2H), 1.50 (d, *J* = 6.4 Hz, 3H), 1.48–1.45 (m, 1H), 1.35 (s, 9H), 1.12–1.03 (m, 1H), 0.87–0.84 (m, 6H) ppm; ^{13}C NMR (125 MHz, $CDCl_3$) δ 163.95 (d, *J* = 12.8 Hz), 161.97 (d, *J* = 12.8 Hz), 155.92, 151.16, 142.08 (d, *J* = 8.8 Hz), 136.85, 127.63, 126.16, 112.58 (d, *J* = 5.7 Hz), 112.44 (d, *J* = 5.6 Hz), 101.98 (t, *J* = 25.3), 80.08, 72.79, 69.65, 57.54, 54.37, 53.76, 35.09, 33.45, 28.21, 26.45, 25.45, 16.88, 11.01 ppm; ^{19}F NMR (470 MHz, $CDCl_3$) δ −110.29 ppm; MS (APCI) *m/z*: calcd for $C_{28}H_{41}F_2N_2O_6S$ [M + H]$^+$: 571.70; found 571.98.

***tert*-Butyl ((2*S*,3*R*)-1-(3,5-difluorophenyl)-4-((*N*-(2-ethylbutyl)-4-((*S*)-1-hydroxyethyl)phenyl)sulfonamido)-3-hydroxybutan-2-yl)carbamate (24c).**

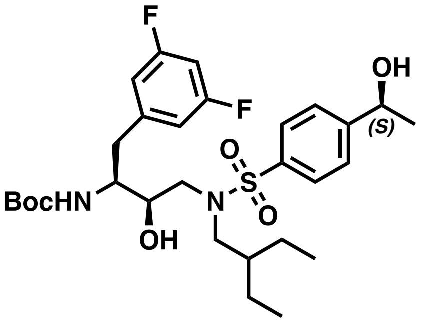

The same procedure was used as described above for compound **24b**. A solution of **23c** (1.30 g, 2.21 mmol) in anhydrous THF (40 mL) was treated with (*R*)-2-methyl-CBS-oxazaborolidine (0.12 g, 4.42 mmol) and a solution of BH_3-THF complex (1 M in THF) 3.30 mL, 3.30 mmol) to give compound **24c** as a white solid (1.17 g, 90%). ^{1}H NMR (500 MHz, $CDCl_3$) δ 7.74 (d, J = 8.4 Hz, 2H), 7.53 (d, J = 8.2 Hz, 2H), 6.79–6.77 (m, 2H), 6.67–6.63 (m, 1H), 4.97 (q, J = 6.4 Hz, 1H), 4.63 (d, J = 8.5 Hz, 1H), 3.77–3.67 (m, 2H), 3.42–3.33 (m, 1H), 3.06–2.99 (m, 4H), 2.90–2.84 (m, 2H), 1.50 (d, J = 6.5, 3H), 1.47–1.38 (m, 2H), 1.34 (s, 9H), 1.32–1.22 (m, 3H), 0.84–0.80 (m, 6H) ppm; ^{13}C NMR (126 MHz, $CDCl_3$) δ 163.94 (d, J = 12.9 Hz), 161.96 (d, J = 13.1 Hz, 2H), 155.21, 151.21, 142.06 (t, J = 9.1 Hz), 136.68, 127.64, 126.18, 112.60 (d, J = 5.6 Hz), 112.44 (d, J = 5.5 Hz), 101.96 (t, J = 25.3 Hz), 80.05, 73.01, 69.61, 55.02, 54.31, 53.78, 39.05, 35.32, 28.20, 25.43, 23.01, 22.73, 10.53, 10.26 ppm; ^{19}F NMR (470 MHz, $CDCl_3$) δ −110.14 ppm; MS (APCI) *m/z*: calcd for $C_{29}H_{43}F_2N_2O_6S$ [M + H]$^+$: 585.73; found 585.30.

***tert*-Butyl ((2*S*,3*R*)-1-(3,5-difluorophenyl)-3-hydroxy-4-((4-((*R*)-1-hydroxyethyl)-*N*-((*S*)-2-methylbutyl)phenyl)sulfonamido)butan-2-yl)carbamate (26b).**

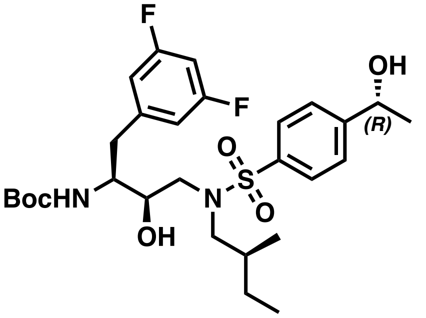

The same procedure was used as described above for compound **24b**. A solution of **23b** (0.50 g, 0.88 mmol) in anhydrous THF (17 mL) was treated with (*S*)-2-methyl-CBS-oxazaborolidine (0.49 g, 1.76 mmol) and a solution of BH_3-THF complex (1 M in THF) (1.5 mL, 1.32 mmol) to give compound **26b** as a white solid (0.41 g, 81%). ^{1}H NMR (500 MHz, $CDCl_3$) δ 7.75 (d, J = 8.3 Hz,

2H), 7.53 (d, J = 8.2 Hz, 2H), 6.79 (d, J = 6.2 Hz, 2H), 6.68–6.63 (m, 1H), 4.98 (q, J = 6.4 Hz, 1H), 4.63 (d, J = 8.1 Hz, 1H), 3.79 (s, 1H), 3.73–3.68 (m, 1H), 3.46–3.38 (m, 1H), 3.07–2.98 (m, 4H), 2.95–2.81 (m, 2H), 1.67–1.59 (m, 2H), 1.51 (d, J = 6.5 Hz, 3H), 1.48–1.44 (m, 1H), 1.36 (s, 9H), 1.12–1.03 (m, 1H), 0.87–0.84 (m, 6H) ppm; ^{13}C NMR (126 MHz, $CDCl_3$) δ 163.95 (d, J = 12.7 Hz), 161.98 (d, J = 13.0 Hz), 155.91, 151.13, 142.08 (t, J = 8.1 Hz), 136.88, 127.64, 126.16, 112.60 (d, J = 5.7 Hz), 112.44 (d, J = 5.6 Hz), 101.98 (t, J = 25.3), 80.08, 72.79, 69.65, 57.55, 54.38, 53.78, 35.09, 33.46, 28.21, 26.44, 25.46, 16.88, 11.01 ppm; ^{19}F NMR (470 MHz, $CDCl_3$) δ −110.26 ppm; MS (APCI) *m/z*: calcd for $C_{28}H_{41}F_2N_2O_6S$ $[M + H]^+$: 571.70; found 571.04.

***tert*-Butyl ((2*S*,3*R*)-1-(3,5-difluorophenyl)-4-((*N*-(2-ethylbutyl)-4-((*R*)-1-hydroxyethyl)phenyl)sulfonamido)-3-hydroxybutan-2-yl)carbamate (26c).**

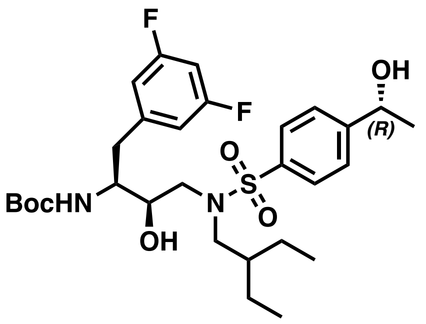

The same procedure was used as described above for compound **24b**. A solution of **23c** (0.55 g, 0.95 mmol) in anhydrous THF (20 mL) was treated with (*S*)-2-methyl-CBS-oxazaborolidine (0.52 g, 1.90 mmol) and a solution of BH_3-THF complex (1 M in THF) (1.62 mL, 1.42 mmol) to give compound **26c** as a white solid (0.43 g, 78%). ^{1}H NMR (500 MHz, $CDCl_3$) δ 7.75 (d, J = 8.3 Hz, 2H), 7.53 (d, J = 8.3 Hz, 2H), 6.79–6.78 (m, 2H), 6.68–6.64 (m, 1H), 4.98 (q, J = 6.4 Hz, 1H), 4.60 (d, J = 8.5 Hz, 1H), 3.77–3.67 (m, 2H), 3.42–3.33 (m, 1H), 3.07–3.00 (m, 4H), 2.89–2.85 (m, 2H), 1.62–1.61 (m, 1H), 1.51 (d, J = 6.4 Hz, 3H), 1.47–1.42 (m, 2H), 1.35 (s, 9H), 1.32–1.28 (m, 3H), 0.82 (t, J = 7.2 Hz, 6H) ppm; ^{13}C NMR (126 MHz, $CDCl_3$) δ 163.95 (d, J = 12.7 Hz), 161.97 (d, J = 12.7 Hz, 2H), 155.86, 151.15, 142.05 (t, J = 8.4 Hz), 136.73, 127.66, 126.18, 112.59 (d, J = 5.7 Hz), 112.44 (d, J = 5.6 Hz), 101.97 (t, J = 25.2 Hz), 80.06, 73.01, 69.65, 55.05, 54.31, 53.81, 39.07, 35.32, 28.21, 26.50, 25.46, 23.02, 22.73, 10.54, 10.26 ppm; ^{19}F NMR (470 MHz, $CDCl_3$) δ −110.28 ppm; MS (APCI) *m/z*: calcd for $C_{29}H_{43}F_2N_2O_6S$ $[M + H]^+$: 585.73; found 585.35.

(3*R*,3a*S*,6a*R*)-Hexahydrofuro[2,3-*b*]furan-3-yl ((2*S*,3*R*)-1-(3,5-difluorophenyl)-3-hydroxy-4-((4-((*S*)-1-hydroxyethyl)-*N*-((*S*)-2-methylbutyl)phenyl)sulfonamido)butan-2-yl)carbamate (7).

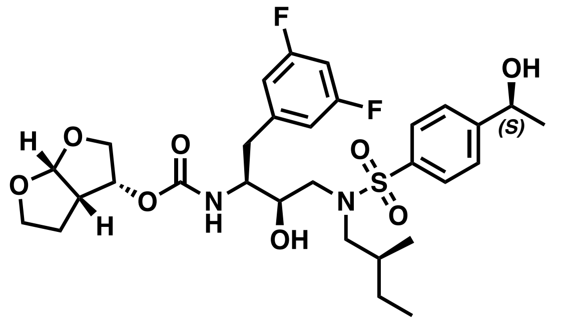

A solution of compound **24b** (0.36 g, 0.63 mmol) in anhydrous CH_2Cl_2 (5 ml) was treated with TFA (4 mL) and the resulting reaction mixture was stirred at room temperature for 2 h. Upon completion of the reaction, solvents were evaporated under reduced pressure. Toluene (5 mL) was added, then evaporated under reduced pressure, and the residue was dried under high vacuum. A solution of the resulting amine salt in anhydrous CH_3CN (8 mL) under argon was cooled to 0 °C and diisopropylethylamine (0.24 g, 1.89 mmol) was added followed by *bis*-THF activated carbonate **25** (0.20 g, 0.75 mmol). After 15 min, the reaction mixture was allowed to warm to room temperature and stirred for 36 h. The solvents were evaporated under reduced pressure, and the residue was purified by automated flash chromatography using a silica gel column (SiliaSep, 25 g, gradient elution with 0–80% EtOAc/hexanes) to give compound **7** (0.38 g, 96%) as a white solid. ^{1}H NMR (500 MHz, $CDCl_3$) δ 7.74 (d, J = 8.3 Hz, 2H), 7.53 (d, J = 8.3 Hz, 2H), 6.74–6.74 (m, 2H), 6.67–6.63 (m, 1H), 5.65 (d, J = 8.8 Hz, 1H), 5.10 (d, J = 8.8 Hz, 1H), 5.05–4.95 (m, 2H), 3.95–3.81 (m, 4H), 3.74–3.67 (m, 2H), 3.64 (d, J = 2.0 Hz, 1H), 3.11–3.00 (m, 4H), 2.96–2.91 (m, 1H), 2.85 (dd, J = 13.3 Hz, 7.4 Hz, 1H), 2.78 (dd, J = 13.9, 9.2 Hz, 1H), 2.26 (d, J = 3.6 Hz, 1H), 1.76–1.68 (m, 1H), 1.61–1.56 (m, 2H), 1.50 (d, J = 6.5 Hz, 3H), 1.49 –1.43 (m, 1H), 1.14–1.05 (m, 1H), 0.87–0.85 (m, 6H); ^{13}C NMR (126 MHz, $CDCl_3$) δ 163.99 (d, J = 12.9 Hz), 161.98 (d, J = 13.0 Hz), 155.38, 151.41, 142.23 (t, J = 9.2 Hz), 136.58, 127.60, 126.20, 112.44 (d, J = 5.5 Hz), 112.29 (d, J = 5.5 Hz), 109.32, 102.13 (t, J = 25.3 Hz), 73.62, 72.72, 70.89, 69.55, 69.51, 57.57, 54.84, 53.73, 45.42, 35.48, 33.51, 26.46, 25.78, 25.41, 16.91, 11.03; ^{19}F NMR (470 MHz, $CDCl_3$) δ −110.01 ppm; HRMS (ESI) *m/z*: calcd for $C_{30}H_{41}F_2N_2O_8S$ $[M + H]^+$: 627.2546; found 627.2543. Anal. HPLC: t_R 10.96 min, purity 98%.

(3*R*,3a*S*,6a*R*)-Hexahydrofuro[2,3-*b*]furan-3-yl ((2*S*,3*R*)-1-(3,5-difluorophenyl)-4-((*N*-(2-ethylbutyl)-4-((*S*)-1-hydroxyethyl)phenyl)sulfonamido)-3-hydroxybutan-2-yl)carbamate (9).

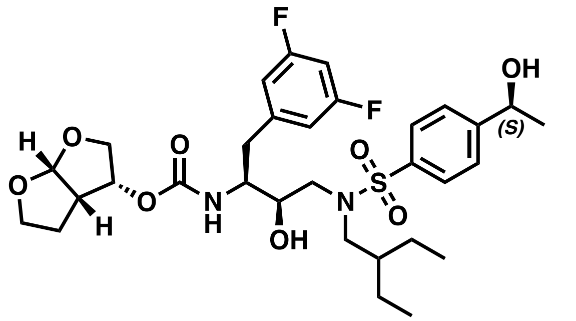

The same procedure was used as described above for compound **7**. A solution of compound **24c** (0.40 g, 0.68 mmol) in CH_2Cl_2 (5 ml) was treated with TFA (4 mL). The resulting amine salt was dissolved in anhydrous CH_3CN (15 mL) and treated with diisopropylethylamine (0.26 g, 2.01 mmol) and *bis*-THF activated carbonate **25** (0.22 g, 0.82 mmol) to give compound **9** (0.41 g, 94%) as a white solid. ^1H NMR (500 MHz, $CDCl_3$) δ 7.74 (d, J = 8.3 Hz, 2H), 7.53 (d, J = 8.2 Hz, 2H), 6.78–6.74 (m, 2H), 6.66–6.53 (m, 1H), 5.64 (d, J = 5.2 Hz, 1H), 5.13 (d, J = 8.8 Hz, 1H), 5.03–4.95 (m, 2H), 3.94–3.82 (m, 4H), 3.72–3.66 (m, 3H), 3.10–3.00 (m, 4H), 2.94–2.89 (m, 2H), 2.75 (dd, J = 13.9 Hz, 9.4 Hz, 1H), 2.37 (d, J = 3.6 Hz, 1H), 1.74–1.69 (m, 1H), 1.56–1.52 (m, 1H), 1.50 (d, J = 6.5 Hz, 3H), 1.45–1.37 (m, 2H), 1.37–1.27 (m, 3H) 0.84–0.80 (m, 6H); ^{13}C NMR (126 MHz, $CDCl_3$) δ 163.95 (d, J = 12.8 Hz), 161.97 (d, J = 12.8 Hz), 155.39, 151.46, 142.03 (t, J = 9.0 Hz), 136.43, 127.62, 126.27, 112.47 (d, J = 5.6 Hz), 112.32 (d, J = 5.5 Hz), 109.32, 102.10 (t, J = 25.38 Hz), 73.61, 73.05, 70.85, 69.53, 69.49, 54.99, 54.84, 45.44, 39.11, 35.60, 25.77, 25.39, 22.99, 22.75, 10.54, 10.29; ^{19}F NMR (470 MHz, $CDCl_3$) δ −109.84 ppm; HRMS (ESI) *m/z*: calcd for $C_{31}H_{43}F_2N_2O_8S$ $[M + H]^+$: 641.2703; found 641.2698. Anal. HPLC: t_R 11.55 min, purity 97%.

(3*R*,3a*S*,6a*R*)-Hexahydrofuro[2,3-*b*]furan-3-yl ((2*S*,3*R*)-1-(3,5-difluorophenyl)-3-hydroxy-4-((4-((*R*)-1-hydroxyethyl)-*N*-((*S*)-2-methylbutyl)phenyl)sulfonamido)butan-2-yl)carbamate (8).

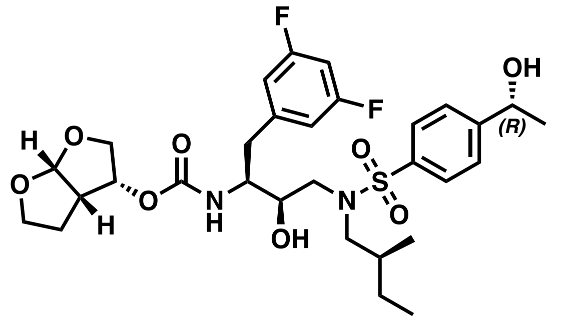

The same procedure was used as described above for compound **7**. A solution of compound **26b** (0.17 g, 0.36 mmol) in CH_2Cl_2 (4 ml) was treated with TFA (3 mL). The resulting amine salt was dissolved in anhydrous CH_3CN (5 mL) and treated with diisopropylethylamine (0.14 g, 1.08 mmol) and *bis*-THF activated carbonate **25** (0.10 g, 0.36 mmol) to give compound **8** (0.17 g, 76%) as a white solid. ^{1}H NMR (500 MHz, $CDCl_3$) δ 7.75 (d, J = 8.3 Hz, 2H), 7.54 (d, J = 8.2 Hz, 2H), 6.79–-6.74 (m, 2H), 6.68–6.64 (m, 1H), 5.66 (d, J = 5.2 Hz, 1H), 5.05–5.01 (m, 2H), 4.98 (q, J = 6.4, 1H), 3.96–3.88 (m, 2H), 3.84–3.81 (m, 2H), 3.77–3.72 (m, 1H), 3.69–3.66 (m, 1H), 3.11–3.02 (m, 4H), 2.99–2.91 (m, 1H), 2.87–2.77 (m, 2H), 1.78–169 (m, 1H), 1.63–1.60 (m, 2H), 1.51 (d, J = 6.5 Hz, 3H), 1.49–1.45 (m, 1H), 1.14–1.05 (m, 1H), 0.88–0.85 (m, 6H) ppm; ^{13}C NMR (126 MHz, $CDCl_3$) δ 163.98 (d, J = 12.9 Hz, 2H), 162.00 (d, J = 12.9 Hz, 2H), 155.36, 151.40, 141.92 (t, J = 8.9 Hz), 136.57, 127.62, 126.24, 112.49 (d, J = 5.4 Hz), 112.33 (d, J = 5.4 Hz), 109.31, 102.14 (t, J = 25.11), 73.64, 72.69, 70.87, 69.56, 69.51, 57.63, 54.81, 53.77, 45.36, 35.54, 33.54, 26.44, 25.78, 25.45, 16.91, 11.02 ppm; ^{19}F NMR (470 MHz, $CDCl_3$) δ −109.76 ppm; HRMS (ESI) *m/z*: calcd for $C_{30}H_{41}F_2N_2O_8S$ $[M + H]^+$: 627.2552; found 627.2542. Anal. HPLC: t_R 10.96 min, purity 98%.

(3*R*,3a*S*,6a*R*)-Hexahydrofuro[2,3-*b*]furan-3-yl ((2*S*,3*R*)-1-(3,5-difluorophenyl)-4-((*N*-(2-ethylbutyl)-4-((*R*)-1-hydroxyethyl)phenyl)sulfonamido)-3-hydroxybutan-2-yl)carbamate (10).

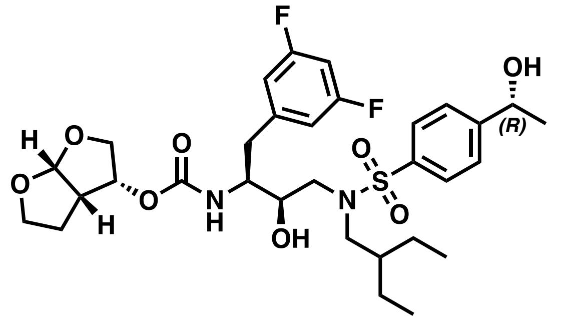

The same procedure was used as described above for compound **7**. A solution of compound **26c** (0.39 g, 0.81 mmol) in CH_2Cl_2 (6 ml) was treated with TFA (5 mL). The resulting amine salt was dissolved in anhydrous CH_3CN (10 mL) and treated with diisopropylethylamine (0.31 g, 2.43 mmol) and *bis*-THF activated carbonate **25** (0.22 g, 0.81 mmol) to give compound **10** (0.35 g, 67%) as a white solid. ^{1}H NMR (500 MHz, $CDCl_3$) δ 7.68 (d, J = 8.3 Hz, 2H), 7.48 (d, J = 8.2 Hz, 2H), 6.71–6.68 (m, 2H), 6.61–6.57 (m, 1H), 5.59 (d, J = 5.1 Hz, 1H), 4.97–4.90 (m, 3H), 3.88–3.81 (m, 2H), 3.78–3.74 (m, 2H), 3.69–3.58 (m, 3H), 3.05–2.97 (m, 3H), 2.93–2.81 (m, 3H), 2.71 (dd, J = 14.0, 9.1 Hz, 1H), 1.71–1.62 (m, 1H), 1.54–1.51 (m, 1H), 1.45 (d, J = 6.5 Hz, 3H), 1.39–1.33 (m, 2H), 1.27–1.22 (m, 3H), 0.78–0.74 (m, 6H) ppm; ^{13}C NMR (126 MHz, $CDCl_3$) δ 163.99 (d, J = 12.7 Hz), 162.01 (d, J = 12.8 Hz), 155.35, 151.40, 141.92 (d, J = 9.1 Hz), 136.42, 127.65, 126.26, 112.48 (d, J = 5.4 Hz), 112.33 (d, J = 5.4 Hz), 109.30, 102.17 (t, J = 25.1 Hz), 73.65, 73.01, 70.78, 69.58, 69.50, 55.12, 54.77, 53.81, 45.34, 39.20, 35.63, 25.75, 25.45, 23.03, 22.75, 10.58, 10.27 ppm; ^{19}F NMR (470 MHz, $CDCl_3$) δ −109.78 ppm; HRMS (ESI) *m/z*: calcd for $C_{31}H_{42}F_2N_2O_8S$ $[M + H]^+$: 641.2708; found 641.2699. Anal. HPLC: t_R 11.58 min, purity 99%.

***tert*-Butyl ((2*S*,3*R*)-3-hydroxy-4-((*N*-isobutyl-1-oxo-2,3-dihydro-1*H*-indene)-5-sulfonamido)-1-phenylbutan-2-yl)carbamate (27a).**

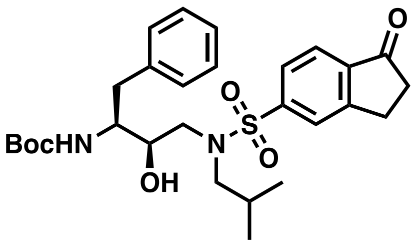

A solution of compound **21a** (1.50 g, 4.46 mmol) in EtOAc (45 mL) was treated with a solution of Na_2CO_3 (0.80 g, 7.57 mmol) in H_2O (45 mL) followed by the addition of 5-indanonesulfonyl chloride (1.03 g, 4.46 mmol). The resulting biphasic reaction mixture was stirred at room temperature for 14 h. The reaction mixture was diluted with EtOAc (150 mL), and the layers were separated. The aqueous layer was further extracted with EtOAc (150 mL). The combined organic portions were washed with saturated aqueous NaCl solution (200 mL), dried (Na_2SO_4), filtered, and concentrated under reduced pressure. The residue was purified by automated flash column chromatography (RediSep Gold, 40 g, gradient elution with 0–80% EtOAc/hexanes), to give compound **27a** (2.24 g, 95%) as a white solid. ^{1}H NMR (500 MHz, $CDCl_3$) δ 7.91 (d, J = 0.5 Hz,

1H), 7.85 (d, J = 8.0 Hz, 1H), 7.76–7.74 (m, 1H), 7.32–7.28 (m, 2H), 7.25–7.21 (m, 3H), 4.63 (d, J = 7.0 Hz, 1H), 3.90 (br s, 1H), 3.84–3.72 (m, 2H), 3.23 (t, J = 6.0 Hz, 2H), 3.16 (d, J = 6.0 Hz, 2H), 3.02–2.97 (m, 2H), 2.94–2.88 (m, 2H), 2.80–2.76 (m, 2H), 1.87 (sep, J = 7.0 Hz, 1H), 1.35 (s, 9H), 0.90 (d, J = 6.5 Hz, 3H), 0.87 (d, J = 6.5 Hz, 3H) ppm; ^{13}C NMR (126 MHz, $CDCl_3$) δ 205.53, 156.36, 155.55, 144.38, 140.16, 137.78, 129.63, 128.69, 126.70, 126.39, 126.01, 124.67, 80.07, 72.72, 58.37, 55.05, 53.37, 36.58, 35.62, 28.37, 27.24, 26.08, 20.20, 19.98 ppm; MS (APCI) *m/z*: calcd for $C_{28}H_{39}N_2O_6S$ [M + H]$^+$: 531.25; found 531.45.

***tert*-Butyl ((2*S*,3*R*)-3-hydroxy-4-((*N*-((*S*)-2-methylbutyl)-1-oxo-2,3-dihydro-1*H*-indene)-5-sulfonamido)-1-phenylbutan-2-yl)carbamate (27b).**

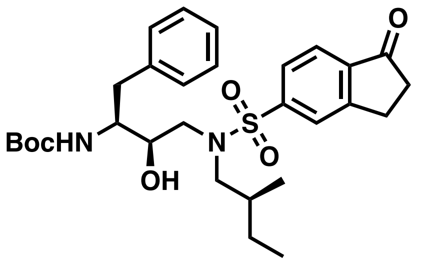

The same procedure was used as described above for compound **27a**. A solution of amino alcohol **21b** (1.00 g, 2.85 mmol) in EtOAc (30 mL) was treated with a solution of Na_2CO_3 (0.52 g, 4.84 mmol) in H_2O (10 mL) and 5-indanonesulfonyl chloride (0.69 g, 3.00 mmol) to provide the compound **27b** (1.50 g, 97%) as a white solid. ^{1}H NMR (500 MHz, $CDCl_3$) δ 7.91 (d, J = 0.5 Hz, 1H), 7.85 (d, J = 8.0 Hz, 1H), 7.76–7.73 (m, 1H), 7.33–7.28 (m, 2H), 7.25–7.21 (m, 3H), 4.61 (d, J = 6.0 Hz, 1H), 3.86 (br s, 1H), 3.81–3.72 (m, 2H), 3.24 (t, J = 5.5 Hz, 2H), 3.16 (app d, J = 7.0 Hz, 2H), 3.07 (dd, J = 13.5, 7.5 Hz, 1H), 2.98 (dd, J = 14.0, 5.0 Hz, 1H), 2.95–2.86 (m, 2H), 2.81–2.76 (m, 2H), 1.68–1.58 (m, 1H), 1.51–1.42 (m, 1H), 1.35 (s, 9H), 1.12–1.02 (m, 1H), 0.88–0.81 (m, 6H) ppm; ^{13}C NMR (126 MHz, $CDCl_3$) δ 205.53, 156.34, 155.55, 144.31, 140.16, 137.68, 129.66, 128.72, 126.74, 126.42, 126.04, 124.67, 80.09, 72.56, 56.96, 54.95, 53.30, 36.59, 35.64, 33.43, 28.39, 26.59, 26.08, 16.99, 11.19 ppm; MS (APCI) *m/z*: calcd for $C_{29}H_{41}N_2O_6S$ [M + H]$^+$: 545.27; found 545.09.

***tert*-Butyl ((2*S*,3*R*)-4-((*N*-(2-ethylbutyl)-1-oxo-2,3-dihydro-1*H*-indene)-5-sulfonamido)-3-hydroxy-1-phenylbutan-2-yl)carbamate (27c).**

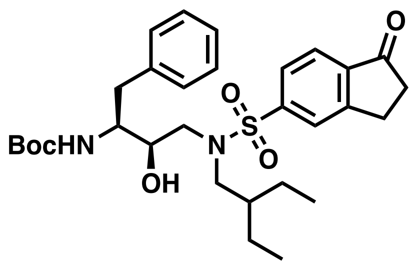

The same procedure was used as described above for compound **27a**. A solution of amino alcohol **21c** (1.00 g, 2.75 mmol) in EtOAc (30 mL) was treated with a solution of Na_2CO_3 (0.50 g, 4.67 mmol) in H_2O (10 mL) and 5-indanonesulfonyl chloride (0.66 g, 2.88 mmol) to provide compound **27c** (1.40 g, 91%) as a white solid. ^{1}H NMR (500 MHz, $CDCl_3$) δ 7.91 (d, J = 0.5 Hz, 1H), 7.86 (d, J = 8.0 Hz, 1H), 7.76–7.74 (m, 1H), 7.32–7.29 (m, 2H), 7.25–7.23 (m, 3H), 4.58 (d, J = 5.0 Hz, 1H), 3.91 (br s, 1H), 3.79–3.74 (m, 2H), 3.24 (t, J = 6.0 Hz, 2H), 3.15 (app d, J = 7.0 Hz, 2H), 3.09 (dd, J = 13.5, 8.0 Hz, 1H), 3.01–2.88 (m, 3H), 2.79–2.77 (m, 2H), 1.52–1.43 (m, 1H), 1.42–1.23 (m, 4H), 1.35 (s, 9H, overlapping), 0.86–0.78 (m, 6H) ppm; ^{13}C NMR (126 MHz, $CDCl_3$) δ 205.53, 156.31, 155.56, 144.17, 140.17, 137.65, 129.64, 128.70, 126.72, 126.42, 126.06, 124.68, 80.06, 72.82, 54.89, 54.53, 53.33, 38.99, 36.59, 35.81, 28.38, 26.08, 23.12, 22.86, 10.68, 10.44 ppm; MS (APCI) *m/z*: calcd for $C_{30}H_{43}N_2O_6S$ $[M + H]^+$: 559.28; found 559.25.

***tert*-Butyl ((2*S*,3*R*)-1-(3,5-difluorophenyl)-3-hydroxy-4-((*N*-((*S*)-2-methylbutyl)-1-oxo-2,3-dihydro-1*H*-indene)-5-sulfonamido)butan-2-yl)carbamate (28b).**

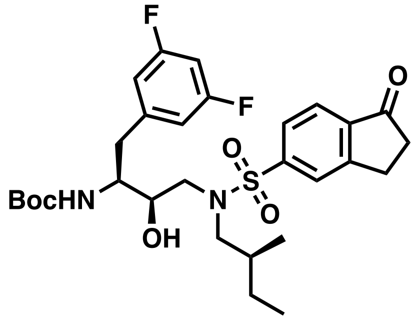

The same procedure was used as described above for compound **27a**. A solution of amino alcohol **22b** (0.34 g, 0.88 mmol) in EtOAc (15 mL) was treated with a solution of Na_2CO_3 (0.16 g, 1.50 mmol) in H_2O (5 mL) and 5-indanonesulfonyl chloride (0.21 g, 0.92 mmol) to provide compound **28b** (0.45 g, 88%) as a white solid. ^{1}H NMR (500 MHz, $CDCl_3$) δ 7.93 (br s, 1H), 7.88 (d, J = 8.0 Hz, 1H), 7.77 (d, J = 8.0 Hz, 1H), 6.83–6.76 (m, 2H), 6.67 (t, J = 8.5 Hz, 1H), 4.62 (d, J = 8.0 Hz, 1H), 3.95 (br s, 1H), 3.86–3.76 (m, 1H), 3.75–3.67 (m, 1H), 3.25 (t, J = 5.5 Hz, 2H), 3.20–3.10

(m, 2H), 3.07 (dd, J = 14.0, 8.0 Hz, 1H), 3.01 (dd, J = 14.0, 4.0 Hz, 1H), 2.91 (dd, J = 13.0, 7.5 Hz, 2H), 2.79 (t, J = 6.0 Hz, 2H), 1.66–1.57 (m, 1H), 1.52–1.43 (m, 1H), 1.37 (s, 9H), 1.13–1.04 (m, 1H), 0.90–0.83 (m, 6H) ppm; ^{13}C NMR (126 MHz, $CDCl_3$) δ 205.46, 164.13 (d, J = 12.9 Hz), 162.15 (d, J = 13.0 Hz), 156.18, 155.63, 143.98, 142.06 (t, J = 9.3 Hz), 140.32, 126.40, 126.07, 124.77, 112.58 (d, J = 5.4 Hz), 112.43 (d, J = 5.5 Hz), 102.22 (t, J = 25.1 Hz), 80.40, 72.80, 57.43, 54.65, 53.64, 36.59, 35.13, 33.58, 28.35, 26.61, 26.09, 17.00, 11.17 ppm; ^{19}F NMR (470 MHz, $CDCl_3$) δ −110.10 ppm.

***tert*-Butyl ((2*S*,3*R*)-1-(3,5-difluorophenyl)-4-((*N*-(2-ethylbutyl)-1-oxo-2,3-dihydro-1*H*-indene)-5-sulfonamido)-3-hydroxybutan-2-yl)carbamate (28c).**

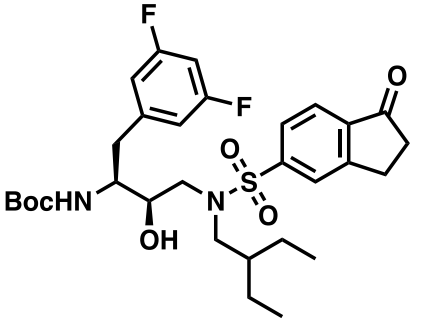

The same procedure was used as described above for compound **27a**. A solution of amino alcohol **22c** (0.35 g, 0.87 mmol) in EtOAc (15 mL) was treated with a solution of Na_2CO_3 (0.16 g, 1.50 mmol) in H_2O (5 mL) and 5-indanonesulfonyl chloride (0.21 g, 0.92 mmol) to provide compound **28c** (0.43 g, 83%) as a white solid. ^{1}H NMR (500 MHz, $CDCl_3$) δ 7.93 (br s, 1H), 7.88 (d, J = 8.0 Hz, 1H), 7.79–7.76 (m, 1H), 6.82–6.76 (m, 2H), 6.67 (tt, J = 9.0, 2.5 Hz, 1H), 4.58 (d, J = 8.0 Hz, 1H), 3.99 (br s, 1H), 3.82–3.75 (m, 1H), 3.74–3.67 (m, 1H), 3.25 (t, J = 6.0 Hz, 2H), 3.18–3.06 (m, 3H), 3.03 (dd, J = 14.0, 4.0 Hz, 1H), 2.96 (dd, J = 13.0, 6.5 Hz, 1H), 2.93–2.85 (m, 1H), 2.83–2.76 (m, 2H), 1.52–1.45 (m, 1H), 1.43–1.22 (m, 4H), 1.36 (s, 9H, overlapping), 0.83 (td, J = 7.5, 2.0 Hz, 6H) ppm; ^{13}C NMR (126 MHz, $CDCl_3$) δ 205.46, 164.12 (d, J = 12.7 Hz), 162.15 (d, J = 13.1 Hz), 156.11, 155.64, 143.81, 142.04 (t, J = 9.2 Hz), 140.33, 126.41, 126.11, 124.79, 112.57 (d, J = 6.0 Hz), 112.42 (d, J = 5.6 Hz), 102.21 (t, J = 24.9 Hz), 80.37, 73.04, 54.99, 54.56, 53.71, 39.18, 36.59, 35.36, 28.34, 26.10, 23.12, 22.88, 10.67, 10.42 ppm; ^{19}F NMR (470 MHz, $CDCl_3$) δ −110.11 ppm.

(3*R*,3a*S*,6a*R*)-Hexahydrofuro[2,3-*b*]furan-3-yl ((2*S*,3*R*)-3-hydroxy-4-((*N*-isobutyl-1-oxo-2,3-dihydro-1*H*-indene)-5-sulfonamido)-1-phenylbutan-2-yl)carbamate (29a).

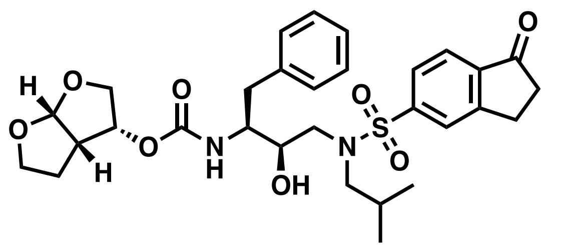

A solution of compound **27a** (0.58 g, 1.09 mmol) in anhydrous CH_2Cl_2 (8 mL) was treated with TFA (6 mL). After stirring the reaction mixture at the room temperature for 2 h, the solvents were evaporated under reduced pressure. Toluene (5 mL) was added, then evaporated under reduced pressure, and the residue was dried under high vacuum. A solution of the resulting amine salt was in anhydrous CH_3CN (20 mL) was cooled to 0 °C under argon and treated with diisopropylethylamine (0.43 g, 3.30 mmol) followed by *bis*-THF activated carbonate **25** (0.33 g, 1.21 mmol). After 15 min, the reaction mixture was allowed to warm to room temperature and stirred for 36 h. The solvents were evaporated under reduced pressure, and the residue was purified by automated flash column chromatography using a silica gel column (RediSep Gold, 24 g, gradient elution with 0–10% methanol/CH_2Cl_2) to give compound **29a** (0.50 g, 78%) as a white solid. ^{1}H NMR (500 MHz, $CDCl_3$) δ 7.91 (br s, 1H), 7.87 (d, J = 8.0 Hz, 1H), 7.75 (d, J = 8.0 Hz, 1H), 7.31–7.26 (m, 2H), 7.24–7.19 (m, 3H), 5.64 (d, J = 5.0 Hz, 1H), 5.02 (q, J = 6.5 Hz, 1H), 4.96 (d, J = 8.5 Hz, 1H), 3.94 (dd, J = 9.5, 6.5 Hz, 1H), 3.92–3.84 (m, 2H), 3.83 (dd, J = 8.0, 2.0 Hz, 1H), 3.71–3.64 (m, 2H), 3.58 (br s, 1H), 3.26–3.19 (m, 3H), 3.10–3.01 (m, 3H), 2.91–2.87 (m, 2H), 2.82–2.77 (m, 3H), 1.86 (sep, J = 7.0 Hz, 1H), 1.68–1.59 (m, 1H), 1.46 (dd, J = 13.0, 5.5 Hz, 1H), 0.93 (d, J = 6.5 Hz, 3H), 0.88 (d, J = 6.5 Hz, 3H) ppm; ^{13}C NMR (126 MHz, $CDCl_3$) δ 205.45, 155.71, 155.61, 144.07, 140.29, 137.60, 129.44, 128.75, 126.83, 126.36, 126.01, 124.75, 109.43, 73.69, 72.89, 70.93, 69.71, 58.69, 55.38, 53.61, 45.51, 36.57, 35.69, 27.37, 26.09, 25.95, 20.23, 19.97 ppm; MS (APCI) *m/z*: calcd for $C_{30}H_{39}N_2O_8S$ [M + H]$^+$: 587.71; found 587.15.

(3*R*,3a*S*,6a*R*)-Hexahydrofuro[2,3-*b*]furan-3-yl ((2*S*,3*R*)-3-hydroxy-4-((*N*-((*S*)-2-methylbutyl)-1-oxo-2,3-dihydro-1*H*-indene)-5-sulfonamido)-1-phenylbutan-2-yl)carbamate (29b).

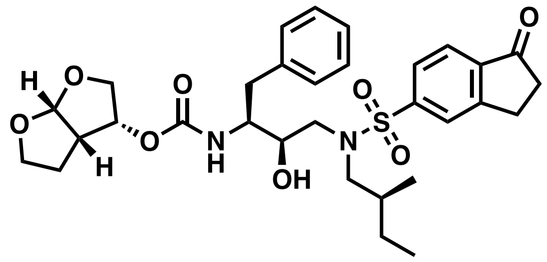

The same procedure was used as described above for compound **29a**. A solution of compound **27b** (0.61 g, 1.12 mmol) in anhydrous CH_2Cl_2 (10 mL) was treated with TFA (10 mL). The resulting amine salt was dissolved in anhydrous CH_3CN (20 mL) and treated with diisopropylethylamine (0.43 g, 3.36 mmol) and *bis*-THF activated carbonate **25** (0.37 g, 1.34 mmol) to provide compound **29b** (0.55 g, 82%) as a white solid. ^{1}H NMR (500 MHz, $CDCl_3$) δ 7.91 (br s, 1 H), 7.87 (d, J = 8.5 Hz, 1H), 7.75 (d, J = 7.5 Hz, 1H), 7.31–7.27 (m, 2H), 7.23–7.21 (m, 3H), 5.64 (d, J = 5.5 Hz, 1H), 5.03 (q, J = 6.5 Hz, 1H), 4.93 (d, J = 8.5 Hz, 1 H), 3.95 (dd, J = 9.5, 6.5 Hz, 1H), 3.91–3.82 (m, 3H), 3.71–3.65 (m, 2H), 3.55 (br s, 1H), 3.25–3.18 (m, 3H), 3.13–3.06 (m, 3H), 2.94–2.81 (m, 3H), 2.80–2.77 (m, 2H), 1.69–1.60 (m, 2H), 1.54–1.44 (m, 2H), 1.14–1.04 (m, 1H), 0.86 (t, J = 7.5 Hz, 3H), 0.84 (d, J = 6.5 Hz, 3H, overlapping) ppm; ^{13}C NMR (126 MHz, $CDCl_3$) δ 205.43, 155.71, 155.61, 143.96, 140.30, 137.51, 129.47, 128.78, 126.87, 126.40, 126.04, 124.76, 109.42, 73.71, 72.74, 70.91, 69.72, 57.27, 55.31, 53.59, 45.50, 36.57, 35.69, 33.56, 26.51, 26.10, 25.96, 17.01, 11.17 ppm; MS (APCI) *m/z*: calcd for $C_{31}H_{41}N_2O_8S$ [M + H]$^+$: 601.73; found 601.15.

(3*R*,3a*S*,6a*R*)-Hexahydrofuro[2,3-*b*]furan-3-yl ((2*S*,3*R*)-4-((*N*-(2-ethylbutyl)-1-oxo-2,3-dihydro-1*H*-indene)-5-sulfonamido)-3-hydroxy-1-phenylbutan-2-yl)carbamate (29c).

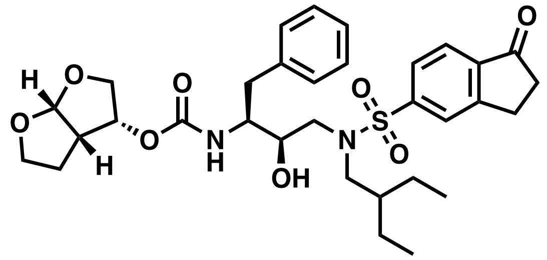

The same procedure was used as described above for compound **29a**. A solution of compound **27c** (0.72 g, 1.29 mmol) in anhydrous CH_2Cl_2 (10 mL) was treated with TFA (10 mL). The resulting amine salt was dissolved in anhydrous CH_3CN (20 mL) and treated with diisopropylethylamine (0.50 g, 3.87 mmol) and *bis*-THF activated carbonate **25** (0.42 g, 1.55 mmol) to give compound **29c** (0.65 g, 82%) as a white solid. ^{1}H NMR (500 MHz, $CDCl_3$) δ 7.92 (br s, 1H), 7.87 (d, J = 8.0

Hz, 1H), 7.76 (d, J = 7.5 Hz, 1H), 7.30–7.27 (m, 2H), 7.22–7.19 (m, 3H), 5.63 (d, J = 5.5 Hz, 1H), 5.01 (q, J = 6.0 Hz, 1H), 4.92 (d, J = 9.0 Hz, 1H), 3.93 (dd, J = 10.0, 6.5 Hz, 1H), 3.91–3.81 (m, 3H), 3.70–3.63 (m, 3H), 3.24 (t, J = 6.0 Hz, 2H), 3.19 (dd, J = 15.5, 9.0 Hz, 1H), 3.13–3.05 (m, 3H), 2.95–2.87 (m, 2H), 2.85–2.77 (m, 3H), 1.67–1.58 (m, 1H), 1.50–1.38 (m, 3H), 1.35–1.22 (m, 3H), 0.85–0.79 (m, 6H) ppm; ^{13}C NMR (126 MHz, $CDCl_3$) δ 205.44, 155.69, 155.62, 143.79, 140.31, 137.53, 129.46, 128.76, 126.84, 126.41, 126.07, 124.76, 109.42, 73.69, 73.12, 70.84, 69.70, 55.27, 54.81, 53.66, 45.50, 39.20, 36.57, 35.82, 26.10, 25.93, 23.12, 22.85, 10.73, 10.39 ppm; MS (APCI) *m/z*: calcd for $C_{32}H_{43}N_2O_8S$ $[M + H]^+$: 615.76; found 615.47.

(3*R*,3a*S*,6a*R*)-Hexahydrofuro[2,3-*b*]furan-3-yl ((2*S*,3*R*)-1-(3,5-difluorophenyl)-3-hydroxy-4-((*N*-((*S*)-2-methylbutyl)-1-oxo-2,3-dihydro-1*H*-indene)-5-sulfonamido)butan-2-yl)carbamate (30b).

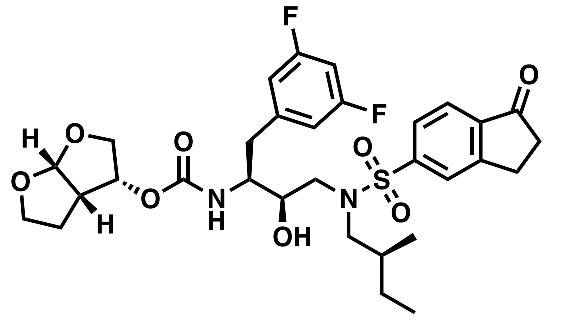

The same procedure was used as described above for compound **29a**. A solution of compound **28b** (0.34 g, 0.59 mmol) in anhydrous CH_2Cl_2 (5 mL) was treated with TFA (5 mL). The resulting amine salt was dissolved in anhydrous CH_3CN (10 mL) and treated with diisopropylethylamine (0.29 mL, 1.77 mmol) and *bis*-THF activated carbonate **25** (0.19 g, 0.70 mmol) to provide compound **30b** (0.29 g, 77%) as a white solid. ^{1}H NMR (500 MHz, $CDCl_3$) δ 7.93 (br s, 1H), 7.89 (d, J = 8.0 Hz, 1H), 7.77 (d, J = 8.0 Hz, 1H), 6.80–6.74 (m, 2H), 6.67 (t, J = 9.0 Hz, 1H), 5.67 (d, J = 5.0 Hz, 1H), 5.05 (q, J = 6.0 Hz, 1H), 5.00 (d, J = 8.5 Hz, 1H), 3.96 (dd, J = 9.5, 6.0 Hz, 1H), 3.93–3.82 (m, 3H), 3.79–3.69 (m, 2H), 3.63 (br s, 1H), 3.26 (t, J = 6.0 Hz, 2H), 3.18 (dd, J = 15.0, 8.0 Hz, 1H), 3.14–3.03 (m, 3H), 2.99–2.92 (m, 1H), 2.89 (dd, J = 13.5, 7.0 Hz, 1H), 2.86–2.77 (m, 3H), 1.80–1.69 (m, 1H), 1.67–1.56 (m, 2H), 1.54–1.44 (m, 1H), 1.16–1.05 (m, 1H), 0.87 (t, J = 7.5 Hz, 3H), 0.86 (d, J = 6.5 Hz, 3H, overlapping) ppm; ^{13}C NMR (126 MHz, $CDCl_3$) δ 205.38, 164.15 (d, J = 12.9 Hz), 162.17 (d, J = 13.1 Hz), 155.68, 155.58, 143.76, 141.79 (t, J = 9.0 Hz), 140.43, 126.39, 126.07, 124.85, 112.48 (d, J = 5.7 Hz), 112.33 (d, J = 5.2 Hz), 109.42, 102.40 (t,

J = 25.1 Hz), 73.89, 72.75, 70.90, 69.63, 57.52, 54.98, 53.67, 45.53, 36.58, 35.43, 33.67, 26.52, 26.11, 25.89, 17.02, 11.15 ppm; ^{19}F NMR (470 MHz, $CDCl_3$) δ −109.59 ppm; MS (APCI) *m/z*: calcd for $C_{31}H_{39}F_2N_2O_8S$ $[M + H]^+$: 637.24; found 637.42.

(3*R*,3a*S*,6a*R*)-Hexahydrofuro[2,3-*b*]furan-3-yl ((2*S*,3*R*)-1-(3,5-difluorophenyl)-4-((*N*-(2-ethylbutyl)-1-oxo-2,3-dihydro-1*H*-indene)-5-sulfonamido)-3-hydroxybutan-2-yl)carbamate (30c).

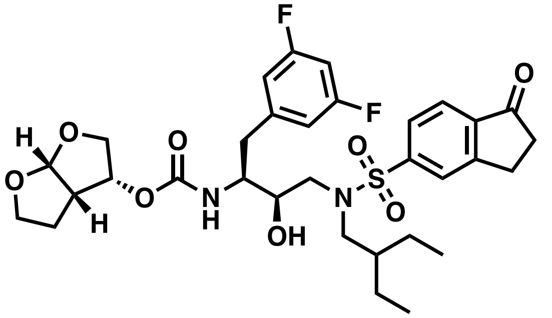

The same procedure was used as described above for compound **29a**. A solution of compound **28c** (0.43 g, 0.72 mmol) in anhydrous CH_2Cl_2 (7 mL) was treated with TFA (7 mL). The resulting amine salt was dissolved in anhydrous CH_3CN (15 mL) and treated with diisopropylethylamine (0.28 g, 2.17 mmol) and *bis*-THF activated carbonate **25** (0.21 g, 0.79 mmol) to provide compound **30c** (0.38 g, 80%) as a white solid. ^{1}H NMR (500 MHz, $CDCl_3$) δ 7.93 (br s, 1H), 7.89 (d, J = 8.0 Hz, 1H), 7.78 (d, J = 8.0 Hz, 1H), 6.81–6.74 (m, 2H), 6.67 (t, J = 9.0 Hz, 1H), 5.66 (d, J = 5.0 Hz, 1H), 5.04 (q, J = 6.0 Hz, 1H), 4.97 (d, J = 9.0 Hz, 1H), 3.95 (dd, J = 9.5, 6.0 Hz, 1H), 3.90 (td, J = 8.5, 2.0 Hz, 1H), 3.88–3.80 (m, 2H), 3.78–3.68 (m, 3H), 3.26 (t, J = 5.5 Hz, 2H), 3.17 (dd, J = 15.0, 8.0 Hz, 1H), 3.13–3.04 (m, 3H), 2.98–2.90 (m, 2H), 2.84–2.76 (m, 3H), 1.79–1.68 (m, 1H), 1.60–1.54 (m, 1H), 1.50–1.38 (m, 2H), 1.37–1.23 (m, 3H), 0.87–0.79 (m, 6H) ppm; ^{13}C NMR (126 MHz, $CDCl_3$) δ 205.38, 164.16 (d, J = 12.8 Hz), 162.18 (d, J = 12.6 Hz), 155.69, 155.57, 143.57, 141.80 (t, J = 9.0 Hz), 140.45, 126.41, 126.10, 124.87, 112.47 (d, J = 5.8 Hz), 112.32 (d, J = 5.5 Hz), 109.42, 102.39 (t, J = 25.1 Hz), 73.89, 73.11, 70.82, 69.62, 55.07, 54.95, 53.76, 45.52, 39.34, 36.58, 35.58, 26.12, 25.86, 23.13, 22.86, 10.75, 10.38 ppm; ^{19}F NMR (470 MHz, $CDCl_3$) δ −109.59 ppm; MS (APCI) *m/z*: calcd for $C_{32}H_{41}F_2N_2O_8S$ $[M + H]^+$: 651.74; found 651.40.

(3*R*,3a*S*,6a*R*)-Hexahydrofuro[2,3-*b*]furan-3-yl ((2*S*,3*R*)-3-hydroxy-4-(((*S*)-1-hydroxy-*N*-isobutyl-2,3-dihydro-1*H*-indene)-5-sulfonamido)-1-phenylbutan-2-yl)carbamate (11).

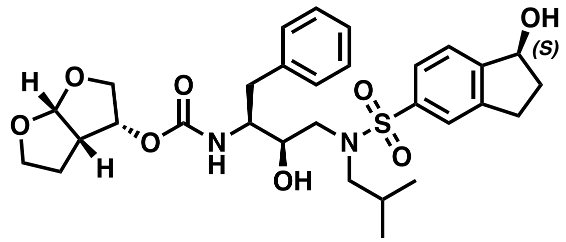

A solution of compound **29a** (0.15 g, 0.26 mmol) in anhydrous CH_2Cl_2 (5 mL) under argon was cooled to 0 °C and treated slowly with a mixture of HCO_2H and Et_3N (1:2 ratio, 15 mL) over 10 min. The reaction mixture was allowed to warm to room temperature and stirred for 30 min. Then, Noyori asymmetric transfer hydrogenation catalyst RuCl[(*S*,*S*)-Ts-DPEN](mesitylene) (5 mol%) (8.0 mg, 0.013 mmol) was added and the reaction mixture was stirred at room temperature for 72 h. The reaction was then quenched with saturated aqueous $NaHCO_3$ solution and extracted with CH_2Cl_2 (3 × 30 mL). The combined organic portions were dried (Na_2SO_4), filtered, and concentrated under reduced pressure. The residue was purified by automated flash column chromatography using a silica gel column (RediSep Gold, 24 g, gradient elution with 0–10% methanol/CH_2Cl_2) to provide the target compound **11** (0.12 g, 78%) as a white solid. ^{1}H NMR (500 MHz, $CDCl_3$) δ 7.67–7.63 (m, 2H), 7.55 (d, *J* = 8.0 Hz, 1H), 7.31–7.26 (m, 2H), 7.24–7.18 (m, 3H), 5.64 (d, *J* = 5.0 Hz, 1H), 5.29 (t, *J* = 6.5 Hz, 1H), 5.00 (q, *J* = 6.5 Hz, 1H), 4.91 (d, *J* = 8.5 Hz, 1H), 3.94 (dd, *J* = 10.0, 6.5 Hz, 1H), 3.91–3.81 (m, 3H), 3.72–3.60 (m, 2H), 3.64 (br s, 1H, overlapping), 3.20–3.05 (m, 3H), 3.04–2.96 (m, 2H), 2.93–2.86 (m, 2H), 2.86–2.76 (m, 2H), 2.62–2.54 (m, 1H), 2.07 (br s, 1H), 2.06–1.97 (m, 1H), 1.85 (sep, *J* = 6.5 Hz, 1H), 1.68–1.58 (m, 1H), 1.47 (dd, *J* = 13.0, 5.5 Hz, 1H), 0.95 (d, *J* = 6.5 Hz, 3H), 0.90 (d, *J* = 6.5 Hz, 3H) ppm; ^{13}C NMR (126 MHz, $CDCl_3$) δ 155.61, 150.42, 144.75, 138.04, 137.71, 129.48, 128.69, 126.74, 126.33, 125.12, 123.96, 109.43, 75.79, 73.57, 72.99, 70.96, 69.73, 59.11, 55.29, 53.96, 45.48, 36.19, 35.77, 29.87, 27.46, 25.95, 20.29, 20.01 ppm; HRMS (ESI) *m/z*: calcd for $C_{30}H_{41}N_2O_8S$ [M + H]$^+$: 589.2578; found 589.2573. Anal. HPLC: t_R 9.70 min, purity 99%.

(3*R*,3a*S*,6a*R*)-Hexahydrofuro[2,3-*b*]furan-3-yl ((2*S*,3*R*)-3-hydroxy-4-(((*R*)-1-hydroxy-*N*-isobutyl-2,3-dihydro-1*H*-indene)-5-sulfonamido)-1-phenylbutan-2-yl)carbamate (12).

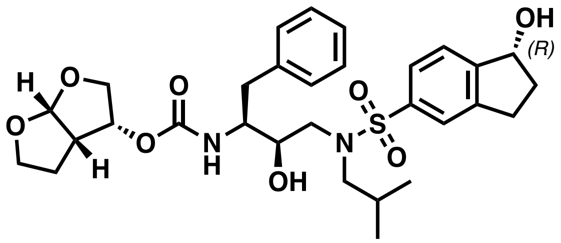

The same procedure was used as described above for compound **11**. A solution of compound **29a** (0.15 g, 0.26 mmol) in anhydrous CH_2Cl_2 (5 mL) was treated with a mixture of HCO_2H and Et_3N (1:2 ratio, 15 mL) followed by the addition of Noyori asymmetric transfer hydrogenation catalyst RuCl[(*R*,*R*)-TsDPEN](mesitylene) (5 mol%) (8.0 mg, 0.013 mmol) to provide the target compound **12** (0.11 g, 72%) as a white solid. ^{1}H NMR (500 MHz, $CDCl_3$) δ 7.68–7.62 (m, 2H), 7.54 (d, J = 7.5 Hz, 1H), 7.31–7.25 (m, 2H), 7.24–7.18 (m, 3H), 5.64 (d, J = 5.0 Hz, 1H), 5.28 (t, J = 6.5 Hz, 1H), 5.01 (q, J = 6.5 Hz, 1H), 4.94 (d, J = 8.5 Hz, 1H), 3.94 (dd, J = 9.5, 6.5 Hz, 1H), 3.91–3.80 (m, 3H), 3.71–3.62 (m, 3H), 3.17 (dd, J = 15.5, 8.5 Hz, 1H), 3.14–3.05 (m, 2H), 3.04–2.97 (m, 2H), 2.93–2.85 (m, 2H), 2.84–2.76 (m, 2H), 2.62–2.54 (m, 1H), 2.09 (br s, 1H), 2.06–1.96 (m, 1H), 1.85 (sep, J = 6.5 Hz, 1H), 1.68–1.57 (m, 1H), 1.46 (dd, J = 13.0, 5.5 Hz, 1H), 0.95 (d, J = 7.0 Hz, 3H), 0.89 (d, J = 6.5 Hz, 3H) ppm; ^{13}C NMR (126 MHz, $CDCl_3$) δ 155.62, 150.40, 144.75, 138.10, 137.70, 129.50, 128.71, 126.76, 126.36, 125.13, 123.98, 109.43, 75.83, 73.59, 72.99, 70.93, 69.73, 59.14, 55.28, 54.00, 45.47, 36.24, 35.75, 29.88, 27.50, 25.94, 20.31, 20.01 ppm; HRMS (ESI) *m/z*: calcd for $C_{30}H_{41}N_2O_8S$ $[M + H]^+$: 589.2578; found 589.2573. Anal. HPLC: t_R 9.67 min, purity 99%.

(3*R*,3a*S*,6a*R*)-Hexahydrofuro[2,3-*b*]furan-3-yl ((2*S*,3*R*)-3-hydroxy-4-(((*S*)-1-hydroxy-*N*-((*S*)-2-methylbutyl)-2,3-dihydro-1*H*-indene)-5-sulfonamido)-1-phenylbutan-2-yl)carbamate (13).

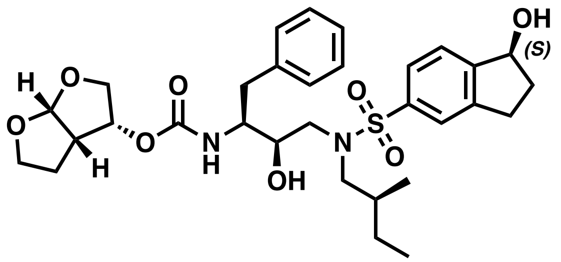

The same procedure was used as described above for compound **11**. A solution of compound **29b** (0.15 g, 0.25 mmol) in anhydrous CH_2Cl_2 (5 mL) was treated with a mixture of HCO_2H and Et_3N

(1:2 ratio, 15 mL) followed by the addition of Noyori asymmetric transfer hydrogenation catalyst RuCl[(*S*,*S*)-Ts-DPEN](mesitylene) (5 mol%) (8.0 mg, 0.013 mmol) to provide the target compound **13** (0.12 g, 79%) as a white solid. ^{1}H NMR (500 MHz, $CDCl_3$) δ 7.67–7.61 (m, 2H), 7.55 (d, *J* = 7.5 Hz, 1H), 7.30–7.26 (m, 2H), 7.24–7.19 (m, 3H), 5.64 (d, *J* = 5.0 Hz, 1H), 5.28 (t, *J* = 6.5 Hz, 1H), 5.01 (q, *J* = 6.0 Hz, 1H), 4.90 (d, *J* = 9.0 Hz, 1H), 3.94 (dd, *J* = 10.0, 6.5 Hz, 1H), 3.92–3.83 (m, 3H), 3.70–3.65 (m, 2H), 3.60 (br s, 1H), 3.18–3.03 (m, 4H), 2.99 (dd, *J* = 15.0, 1.5 Hz, 1H), 2.94–2.86 (m, 2H), 2.85–2.78 (m, 2H), 2.62–2.54 (m, 1H), 2.10 (br s, 1H), 2.05–1.96 (m, 1H), 1.69–1.57 (m, 2H), 1.56–1.49 (m, 2H), 1.15–1.05 (m, 1H), 0.87 (t, *J* = 7.5 Hz, 3H), 0.86 (d, *J* = 6.5 Hz, 3H, overlapping) ppm; ^{13}C NMR (126 MHz, $CDCl_3$) δ 155.60, 150.40, 144.76, 137.97, 137.62, 129.52, 128.73, 126.79, 126.40, 125.11, 124.00, 109.44, 75.83, 73.61, 72.87, 70.95, 69.74, 57.71, 55.21, 53.96, 45.46, 36.23, 35.80, 33.67, 29.88, 26.53, 25.97, 17.07, 11.18 ppm; HRMS (ESI) *m/z*: calcd for $C_{31}H_{43}N_2O_8S$ $[M + H]^+$: 603.2735; found 603.2727. Anal. HPLC: t_R 10.37 min, purity 98%.

(3*R*,3a*S*,6a*R*)-Hexahydrofuro[2,3-*b*]furan-3-yl ((2*S*,3*R*)-4-(((*S*)-*N*-(2-ethylbutyl)-1-hydroxy-2,3-dihydro-1*H*-indene)-5-sulfonamido)-3-hydroxy-1-phenylbutan-2-yl)carbamate (14).

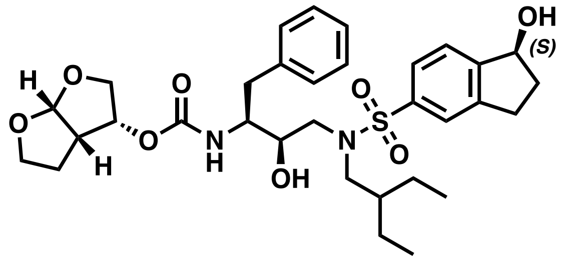

The same procedure was used as described above for compound **11**. A solution of compound **29c** (0.15 g, 0.24 mmol) in anhydrous CH_2Cl_2 (5 mL) was treated with a mixture of HCO_2H and Et_3N (1:2 ratio, 15 mL) followed by the addition of Noyori asymmetric transfer hydrogenation catalyst RuCl[(*S*,*S*)-Ts-DPEN](mesitylene) (5 mol%) (7.5 mg, 0.012 mmol) to provide the target compound **14** (0.12 g, 81%) as a white solid. ^{1}H NMR (500 MHz, $CDCl_3$) δ 7.65–7.63 (m, 2H), 7.55 (d, *J* = 7.9 Hz, 1H), 7.28–7.26 (m, 2H), 7.22–7.19 (m, 3H), 5.64 (d, *J* = 5.1 Hz, 1H), 5.29 (t, *J* = 6.5 Hz, 1H), 5.00 (q, *J* = 6.7 Hz, 1H), 4.88 (d, *J* = 9.0 Hz, 1H), 3.92 (dd, *J* = 9.5, 6.5 Hz, 1H), 3.89–3.80 (m, 3H), 3.71 (br s, 1H), 3.70–3.63 (m, 2H), 3.16–3.06 (m, 4H), 2.98 (dd, *J* = 15.5, 1.5 Hz, 1H), 2.91–2.83 (m, 3H), 2.80 (dd, *J* = 14.0, 9.0 Hz, 1H), 2.62–2.54 (m, 1H), 2.10 (br s, 1H), 2.05–1.97 (m, 1H), 1.67–1.58 (m, 1H), 1.50–1.41 (m, 3H), 1.35–1.25 (m, 3H), 0.85–0.81 (m, 6H)

ppm; ^{13}C NMR (126 MHz, $CDCl_3$) δ 155.59, 150.42, 144.77, 137.80, 137.63, 129.51, 128.73, 126.78, 126.41, 125.13, 124.03, 109.43, 75.83, 73.60, 73.24, 70.88, 69.73, 55.18, 54.00, 45.46, 39.33, 36.23, 35.93, 29.88, 25.94, 23.20, 22.89, 10.78, 10.42 ppm; HRMS (ESI) *m/z*: calcd for $C_{32}H_{45}N_2O_8S$ $[M + H]^+$: 617.2891; found 617.2892. Anal. HPLC: t_R 11.06 min, purity 99%.

(3*R*,3a*S*,6a*R*)-Hexahydrofuro[2,3-*b*]furan-3-yl ((2*S*,3*R*)-1-(3,5-difluorophenyl)-3-hydroxy-4-(((*S*)-1-hydroxy-*N*-((*S*)-2-methylbutyl)-2,3-dihydro-1*H*-indene)-5-sulfonamido)butan-2-yl)carbamate (17).

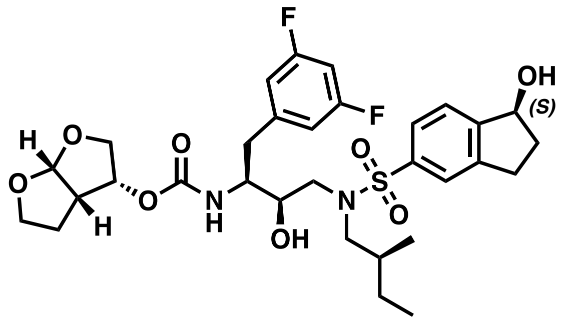

The same procedure was used as described above for compound **11**. A solution of compound **30b** (0.15 g, 0.23 mmol) in anhydrous CH_2Cl_2 (5 mL) was treated with a mixture of HCO_2H and Et_3N (1:2 ratio, 15 mL) followed by the addition of Noyori asymmetric transfer hydrogenation catalyst RuCl[(*S*,*S*)-Ts-DPEN](mesitylene) (5 mol%) (7.50 mg, 0.012 mmol) to provide the target compound **17** (0.10 g, 70%) as a white solid. ^1H NMR (500 MHz, $CDCl_3$) δ 7.69–7.62 (m, 2H), 7.56 (d, J = 7.5 Hz, 1H), 6.80–6.73 (m, 2H), 6.67 (t, J = 9.0 Hz, 1H), 5.66 (d, J = 5.5 Hz, 1H), 5.29 (t, J = 6.5 Hz, 1H), 5.07–4.95 (m, 2H), 3.96 (dd, J = 9.5, 7.0 Hz, 1H), 3.91 (td, J = 8.5, 2.0 Hz, 1H), 3.88–3.80 (m, 2H), 3.78–3.72 (m, 1H), 3.69 (dd, J = 9.5, 6.0 Hz, 1H), 3.16–2.98 (m, 5H), 2.97–2.91 (m, 1H), 2.90–2.76 (m, 3H), 2.63–2.55 (m, 1H), 2.06–1.97 (m, 1H), 1.79–1.69 (m, 1H), 1.65–1.57 (m, 2H), 1.55–1.47 (m, 1H), 1.16–1.06 (m, 1H), 0.88 (t, J = 7.5 Hz, 3H), 0.87 (d, J = 6.5 Hz, 3H, overlapping) ppm; ^{13}C NMR (126 MHz, $CDCl_3$) δ 164.12 (d, J = 12.6 Hz), 162.14 (d, J = 12.6 Hz), 155.50, 150.54, 144.86, 141.92 (t, J = 9.0 Hz), 137.80, 126.38, 125.18, 123.98, 112.49 (d, J = 5.9 Hz), 112.34 (d, J = 5.6 Hz), 109.43, 102.10 (t, J = 25.2 Hz), 75.83, 73.78, 72.85, 70.95, 69.65, 57.86, 54.91, 53.95, 45.49, 36.22, 35.61, 33.73, 29.89, 26.56, 25.89, 17.07, 11.16 ppm; ^{19}F NMR (470 MHz, $CDCl_3$) δ −109.76 ppm; HRMS (ESI) *m/z*: calcd for $C_{31}H_{41}F_2N_2O_8S$ $[M + H]^+$: 639.2546; found 639.2540. Anal. HPLC: t_R 10.95 min, purity 98%.

(3*R*,3a*S*,6a*R*)-Hexahydrofuro[2,3-*b*]furan-3-yl ((2*S*,3*R*)-1-(3,5-difluorophenyl)-4-(((*S*)-*N*-(2-ethylbutyl)-1-hydroxy-2,3-dihydro-1*H*-indene)-5-sulfonamido)-3-hydroxybutan-2-yl)carbamate (18).

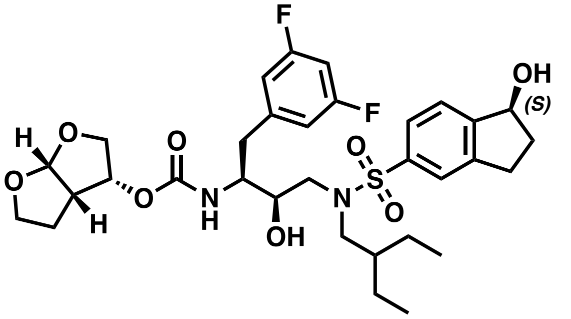

The same procedure was used as described above for compound **11**. A solution of compound **30c** (0.15 g, 0.23 mmol) in anhydrous CH_2Cl_2 (5 mL) was treated with a mixture of HCO_2H and Et_3N (1:2 ratio, 15 mL) followed by the addition of Noyori asymmetric transfer hydrogenation catalyst RuCl[(*S*,*S*)-Ts-DPEN](mesitylene) (5 mol%) (7.50 mg, 0.012 mmol) to provide the target compound **18** (0.11 g, 73%) as a white solid. ^{1}H NMR (500 MHz, $CDCl_3$) δ 7.69–7.63 (m, 2H), 7.57 (d, J = 7.5 Hz, 1H), 6.80–6.74 (m, 2H), 6.67 (t, J = 9.0 Hz, 1H), 5.66 (d, J = 5.5 Hz, 1H), 5.30 (t, J = 6.5 Hz, 1H), 5.03 (q, J = 6.0 Hz, 1H), 4.96 (d, J = 8.5 Hz, 1H), 3.94 (dd, J = 9.5, 6.0 Hz, 1H), 3.90 (td, J = 8.0, 2.0 Hz, 1H), 3.86–3.78 (m, 2H), 3.78–3.71 (m, 1H), 3.68 (dd, J = 10.0, 6.5 Hz, 1H), 3.16–3.04 (m, 4H), 3.02–2.84 (m, 4H), 2.78 (dd, J = 14.0, 9.0 Hz, 1H), 2.63–2.55 (m, 1H), 2.06–1.97 (m, 1H), 1.78–1.68 (m, 1H), 1.63–1.56 (m, 1H), 1.50–1.39 (m, 2H), 1.38–1.27 (m, 3H), 0.88–0.81 (m, 6H) ppm; ^{13}C NMR (126 MHz, $CDCl_3$) δ 164.12 (d, J = 12.6 Hz), 162.15 (d, J = 12.7 Hz), 155.48, 150.55, 144.88, 141.91 (t, J = 9.0 Hz), 137.64, 126.41, 125.20, 124.02, 112.49 (d, J = 5.2 Hz), 112.33 (d, J = 5.9 Hz), 109.43, 102.30 (t, J = 25.1 Hz), 75.84, 73.79, 73.19, 70.86, 69.64, 55.34, 54.91, 54.00, 45.48, 39.42, 36.24, 35.75, 29.89, 25.87, 23.19, 22.90, 10.77, 10.41 ppm; ^{19}F NMR (470 MHz, $CDCl_3$) δ −109.74 ppm; HRMS (ESI) *m/z*: calcd for $C_{32}H_{43}F_2N_2O_8S$ $[M + H]^+$: 653.2703; found 653.2696. Anal. HPLC: t_R 11.59 min, purity 97%.
